# Supplementary material for: Restricting Branched-Chain Amino Acids within a High-Fat Diet Prevents Obesity
Source: Metabolites. 2022 Apr 7;12(4):334. doi: 10.3390/metabo12040334 (PMC9030079; doi:10.3390/metabo12040334)
Supplement: Supplementary file 1 [file metabolites-12-00334-s001.zip › metabolites-1665439-supplementary.pdf]

Supplemental Material

This Supplemental Material has been provided by the authors to give readers additional information about their work.

Supplement to: Liu M, Huang YH, et al. Restricting branched-chain amino acids within a high-fat diet prevents obesity.

Content

List of participating investigators 4

Supplementary Methods 6

Study participants 6

Demographic and medical information collection, and body composition measurement 7

Serum lipids, glucose, insulin and homeostatic model assessment for insulin resistance (HOMA-IR) measurement 7

Metabolomics profiling 8

Experiment animals 8

Micro CT scanning 9

Isolation of stroma vascular cells (SVCs) and flow cytometric analysis 9

Serum insulin, triglycerides and free fatty acid measurement in mice 9

Statistical analysis 9

Supplementary results 11

Risk of bias assessment 11

Detection difference of the p180 kit and the TMIC assay 11

BMI/obesity associated metabolites 11

Impact of osteoarthritis status on metabolites associated with BMI/obesity 12

Relationships between BMI and obesity associated metabolites and diabetes, and levels of serum glucose, insulin and HOMA-IR 12

Relationships between BMI and obesity associated metabolites and blood lipids profile 12

Diet restriction trial in mice 13

Supplementary tables 14

Table S1. Associations between standardized BMI-associated metabolite concentrations and diabetes 14

Table S2. Associations between standardized obesity-associated metabolite concentrations and diabetes 17

Table S3. Association between standardized BMI-associated metabolite concentrations and serum glucose, insulin and HOMA-IR levels in the CODING cohort 20

Table S4. Association between standardized obesity-associated metabolite concentrations and serum glucose, insulin and HOMA-IR levels in the CODING cohort.................................... 22
Table S5. Association between standardized BMI-associated metabolite concentrations and blood lipids profile in the CODING and NFOAS cohorts 24
Table S6. Association between standardized obesity-associated metabolite concentrations and blood lipids profile in the CODING and NFOAS cohorts 35
Table S7. Association between standardized BMI-associated metabolite concentrations and visceral fat mass in the CODING cohort 46
Table S8. Association between standardized obesity-associated metabolite concentrations and visceral fat mass in the CODING cohort 48
Table S9. Biochemical characteristics of participants in the CODING and NFOAS cohorts .. 50
Table S10. List of metabolites determined using the Biocrates AbsoluteIDQ® p180 kit....... 51
Table S11. List of metabolite concentrations determined using the TMIC Prime Metabolomics Profiling Assay 53
Table S12. Formula of diets used in mouse dietary trial... 55

Supplementary figures.. 57
Figure S1. Forest plots for metabolites associated with BMI 57
Figure S2. Forest plots for metabolites associated with obesity 69
Figure S3. Phospholipid metabolic pathways associated with altered blood lipid profiles in obesity 80
Figure S4. Single whole body projection scan and lean volume of mice 81
Figure S5. Serum levels of free fatty acid and triglycerides and lipids in liver in mice 82
Figure S6. FACS gating strategy for M1 and M2 macrophages in mice adipose tissue........... 83
Figure S7. Traffic light plot for risk of bias assessment for studies included in the meta-analysis 84
Figure S8. Body weights and biochemical parameters in mice in SCD groups....................... 85

References.. 86

List of participating investigators

Investigator	Institute/city	Country
Ming Liu, MSc	Division of Biomedical Sciences (Genetics), Faculty of Medicine, Memorial University of Newfoundland, St. John’s	Canada
Yiheng Huang, MSc	College of Pharmacy, University of Manitoba, Winnipeg	Canada
Hongwei Zhang, MSc	Discipline of Medicine, Faculty of Medicine, Memorial University of Newfoundland, St. John’s	Canada
Dawn Aitken, PhD	Menzies Institute for Medical Research, University of Tasmania, Hobart	Australia
Michael C. Nevitt, PhD, MPH	Epidemiology and Biostatistics, University of California, San Francisco	The United States
Jason S. Rockel, PhD	Osteoarthritis Research Program, Division of Orthopaedics, Schroeder Arthritis Institute, University Health Network, Toronto	Canada
Jean-Pierre Pelletier, MD	Osteoarthritis Research Unit, University of Montreal Hospital Research Centre (CRCHUM), Montreal	Canada
Cora E. Lewis, MD	Department of Epidemiology, University of Alabama, Birmingham	The United States
James Torner, PhD	Department of Epidemiology, University of Iowa, Iowa City, Iowa	The United States
Y. Raja Rampersaud, MD	Osteoarthritis Research Program, Division of Orthopaedics, Schroeder Arthritis Institute, University Health Network, Toronto	Canada
Anthony V. Perruccio, PhD	Osteoarthritis Research Program, Division of Orthopaedics, Schroeder Arthritis Institute, University Health Network, Toronto; Institute of Health Policy, Management and Evaluation, Dalla Lana School of Public Health, University of Toronto, Toronto	Canada
Nizar N. Mahomed, ScD, MPH, MD	Osteoarthritis Research Program, Division of Orthopaedics, Schroeder Arthritis Institute, University Health Network, Toronto; Department of Surgery, University of Toronto, Toronto	Canada
Andrey Furey, MD	Discipline of Surgery, Faculty of Medicine, Memorial University of Newfoundland, St. John’s; Office of the Premier, Government of Newfoundland and Labrador, St. John’s	Canada

Edward W. Randell, PhD	Discipline of Laboratory Medicine, Faculty of Medicine, Memorial University of Newfoundland, St. John’s	Canada
Proton Rahman, MD	Discipline of Medicine, Faculty of Medicine, Memorial University of Newfoundland, St. John’s	Canada
Guang Sun, PhD	Discipline of Medicine, Faculty of Medicine, Memorial University of Newfoundland, St. John’s	Canada
Johanne Martel-Pelletier, PhD	Osteoarthritis Research Unit, University of Montreal Hospital Research Centre (CRCHUM), Montreal	Canada
Mohit Kapoor, PhD	Osteoarthritis Research Program, Division of Orthopaedics, Schroeder Arthritis Institute, University Health Network, Toronto	Canada
Graeme Jones, MD	Menzies Institute for Medical Research, University of Tasmania, Hobart	Australia
David Felson, MD, MPH	Department of Rheumatology, Boston University School of Medicine, Boston; NIHR Manchester Biomedical Research Centre, Manchester Academic Health Science Centre, Manchester University NHS Foundation Trust, Manchester	The United States, The United Kingdom
Dake Qi, MD, PhD	College of Pharmacy, University of Manitoba, Winnipeg	Canada
Guangju Zhai, PhD	Division of Biomedical Sciences (Genetics), Faculty of Medicine, Memorial University of Newfoundland, St. John’s	Canada

*Ming Liu and Yiheng Huang are shared first author.

*Dake Qi and Guangju Zhai are shared senior author.

Supplementary Methods

Study participants

The Complex Diseases in the Newfoundland population: Environment and Genetics (CODING) study is an ongoing large-scale nutritional genetic study of human complex diseases which recruited 3214 healthy individuals aged at least 20 years old that were at least third-generation Newfoundlanders from the province of Newfoundland and Labrador, Canada[1]. Two hundred twenty-six (226) study participants with metabolomics data available were included for the current study. The metabolomic profiling was done previously for other studies[1, 2]. The CODING study was approved by the Health Research Ethics Authority (HREA), Memorial University, St. John's, NL, Canada, with Project Identification Code #10.33. All participants provided written and informed consent.

The Longitudinal Evaluation in the Arthritis Program: Osteoarthritis Study (LEAP-OA) originally aimed to identify prognostic factors in the surgical management of end-stage primary osteoarthritis (OA)[3]. Eight hundred forty-three (843) patients with end-stage OA of the knee or hip scheduled for primary total joint replacement (TJR) surgery were recruited from the Toronto Western Hospital in Toronto, Ontario, Canada between 2013 - 2016. Four hundred ninety-five knee OA patients with metabolomics data available were included in the present study. The LEAP-OA study was approved by the University Health Network Research Ethics Board. Written informed consent was obtained from all patients.

The licofelone/naproxen clinical trial is a multicentre, randomized, double-blind and parallel group study comparing the effects of licofelone and naproxen in 355 patients with knee OA that were recruited from outpatient rheumatology clinics throughout Canada[4, 5]. One hundred eighty-five (185) patients with metabolomics data available[5, 6] were selected for the present study. The original study protocol was approved by a central review board (IRB Institutional Review Board Services, Toronto, ON, Canada) and the institutional review board of the Centre hospitalier de l'Université de Sherbrooke (Sherbrooke, QC, Canada), and all patients gave their oral and written informed consent.

The Multicenter Osteoarthritis Study (MOST) is a longitudinal, prospective, observational study which focus on evaluating factors that affect the course of knee OA[7]. Three thousand twenty-six (3026) men and women aged 50-79 years with pre-existing knee OA or being at high risk for knee OA were recruited and examined at clinical centres at the University of Alabama at Birmingham and University of Iowa between 2003-2005. One thousand two hundred forty-eight (1248) patients with metabolomics data on baseline plasma samples available[8] were included in the current study. The MOST study was approved by local Institutional Review Board (IRB; reference number H-22670) and informed consent was given by all study participants.

The Newfoundland Osteoarthritis Study (NFOAS) aims at identifying novel genetic, epigenetic, and biochemical markers for OA and other musculoskeletal conditions[9, 10]. The study

recruited 1086 patients who underwent total knee or hip replacement surgery largely due to OA with a small number of patients due to other joint diseases between 2011-2017 in St. Clare's Mercy Hospital and Health Science Centre General Hospital in St. John's, Newfoundland and Labrador, Canada. Seven hundred four (704) patients with metabolomic data available[2, 10] were included in the current study. The NFOAS was approved by the Health Research Ethics Authority of Newfoundland and Labrador (HREB # 2011.311) and written consent was obtained from all participants.

The Tasmanian Older Adult Cohort study (TASOAC) is a prospective, population-based cohort study that recruited 1100 participants aged 50-80 years who were randomly selected from the electoral roll in southern Tasmania (population 229,000), Australia, between 2002-2004[11]. Participants were subsequently followed up at 2.6-, 5- and 10-year. Five hundred sixty-six (566) participants with metabolomics data available[2, 11] were included in the current study. The TASOAC study was approved by the Southern Tasmanian Health and Medical Human Research Ethics Committee (Ref. no: H0006488) and written informed consent was obtained from all participants.

Demographic and medical information collection, and body composition measurement

With the exception of TASOAC, data on the date of birth and sex were self-reported in all cohorts and age at recruitment was calculated; height and weight were measured in CODING cohort, retrieved from Eastern Health Meditech Health Care Information System for NFOAS cohort, and self-reported in all other cohorts at the time of recruitment, and body mass index (BMI) was calculated as weight in kg/squared height in meters. All above information for TASOAC was collected at 2.6-year follow-up. Diabetes status at baseline in CODING, LEAP-OA, licofelone/naproxen clinical trial and NFOAS, and that at 2.6-year follow-up in TASOAC were self-reported. Visceral fat mass of participants in CODING cohort was evaluated using CoreScan[1].

Serum lipids, glucose, insulin and homeostatic model assessment for insulin resistance (HOMA-IR) measurement

Blood samples were collected at the time of recruitment after at least 8 hours fasting for all cohorts except for TASOAC, of which fasting blood samples were collected at 2.6-year follow-up. In NFOAS, data on conventional blood lipid profiles including total cholesterol (TC), high-density lipoprotein (HDL) cholesterol, low-density lipoprotein (LDL) cholesterol, non-HDL cholesterol, TC to HDL ratio, and triglycerides (TG) were retrieved from Eastern Health Meditech Health Care Information System. In CODING cohort, serum concentrations of TC, HDL, and TG were detected on an Architech C16000 clinical chemistry analyzer (Abbott Laboratories, IL, USA), LDL was calculated by Friedewald's formula [TC - HDL - TG/2.2][12] and non-HDL was calculated as [TC - HDL]. Concentrations of very-low-density lipoprotein (VLDL) was calculated as [TG/2.2][12] for both cohorts. All concentrations were reported in

mmol/L. Additionally, in CODING cohort, fasting glucose level was measured on an Lx20 analyzer (Beckman Coulter Inc., Fullerton, CA, USA) using Synchron reagents and reported in mmol/L, and fasting insulin level was measured on an Immulite Immunoassay analyzer (Siemans Healthcare GmbH, Erlangen, Germany) and reported in pmol/L. HOMA-IR was calculated as [(Fasting Insulin (mU/L) × Fasting Glucose (mmol/L))/22.5][13].

Metabolomics profiling

The p180 kit quantifies 186 metabolites including acylcarnitines, amino acids, biogenic amines, hexoses and phospho-and sphingolipids (full list of the 186 metabolites is provided in **Table S10**), whereas the TMIC assay measures 143 compounds including acylcarnitines, amino acids and derivatives, amine oxides, biogenic amines, carboxylic acids, monosaccharides, organic acids, phospho-and sphingolipids and vitamins and cofactors (full list of the 143 metabolites is provided in **Table S11**).

The metabolomics profiling with the p180 kit was done either in-house using Waters XEVO TQ MS system (Waters, Mississauga, Ontario, Canada) or at the Metabolomic Innovation Center (TMIC) in Edmonton, Canada, using an API4000 Qtrap® tandem mass spectrometry instrument (Applied Biosystems/MDS Analytical Technologies, Foster City, CA) equipped with an Agilent 1100 HPLC system. The complete analytical process was performed using the MetIQ software package, which is an integral part of the p180 kit. The metabolite concentration was reported in μM. More details of the metabolic profiling method using this kit were described previously[14].

The profiling with the TMIC Prime Metabolomics Profiling Assay[11] was done at the TMIC using an AB SCIEX QTRAP®4000 mass spectrometer (Sciex Canada, Concord, ON, Canada) equipped with an Agilent 1260 series ultra high-performance liquid chromatography (UHPLC) system (Agilent Technologies, Palo Alto, CA, USA) as described previously[15]. The Analyst software 1.6.2 (Concord, ON, Canada) was used to control the entire assay's workflow and the metabolite concentrations were reported in μM.

Experiment animals and dietary regimens

Male C57BL/6J mice were housed in a filter-top cage with free access to food and water under a 12-hour light/12-hour dark cycle at the animal facility in University of Manitoba. All experiments involving mice were conducted in accordance with the Guide for the Care and Use of Laboratory Animals of the National Institutes of Health and were approved by the Internal Animal Committee Review Board of University of Manitoba. The 2/3 reduction of amino acids was based on previous dietary restriction trials in mice and was physiologically relevant [16, 17].

Micro CT scanning

Skyscan 1176 micro-CT system was used to measure mouse whole-body composition. Mice were scanned from cervical vertebrae to mid-tibia in a lying position (50kV, 500 μA source voltage and current, 35 um resolution with 0.5mm Al filter). Reconstruction was performed using NRecon with a ring artefact correction factor of 4 and beam hardening correction factor of 30%. Body fat and lean volume percentages were calculated using CTAn. All software were provided by Skyscan/Bruker, Kontich, Belgium.

Isolation of stroma vascular cells (SVCs) and flow cytometric analysis

Fat pads of mice were collected and minced in Krebs-Ringer bicarbonate (KRB) buffer containing 1% BSA. Following digestion with 300 U/ml type I collagenase (Sigma) at 37°C for 40 minutes, suspension was centrifuged at 500g for 10 minutes. The pellets (containing SVCs) were subsequently subjected to red blood cell lysis in 1× Red Blood Cell Lysis Buffer and then filtered through a 40-μm cell strainer. SVCs were stained with the following antibodies: CD45.2-PerCP (eBioscience; catalog 45-0454-80); F4/80-PE (eBioscience; catalog 12-4801-80); CD11b-APC eFluor 780 (eBioscience; catalog 47-0112-80); CD11c-PE-Cy7 (eBioscience; catalog 25-0114-81); CD301–Alexa Fluor 647 (AbD Serotec; catalog MCA2392A647T). Stained SVCs were analyzed using a Beckman Coulter Cytoflex LX (Brea, CA, USA).

Serum insulin, triglycerides and free fatty acid measurement in mice

Fasting blood was collected at end of the experiment and serum was separated from the whole blood according to the standard protocol. The serum levels of insulin, TG and free fatty acid in mice were quantified with Insulin Mouse Elisa Kit (Thermo Fisher, USA), L-Type Triglyceride M Assay Kit (FUJIFILM, USA), and HR series NEFA-HR Assay Kit (FUJIFILM, USA), respectively, following the manufacturer's instruction.

Statistical analysis

Risk of bias assessment for studies included in the meta-analysis were perfomed with the Joanna Briggs Institute (JBI) Critical Appraisal Checklist for analytical cross-sectional study[18] (last amended in 2017) and visualization of the results was done with R metaconfoundr package. Quality control (QC) procedures for metabolomics data was applied as the following: metabolites were removed from analysis if more than 10% of the samples had values below the limit of detection to minimize the false positive results as a standard practice in metabolomics studies[9]; missing values of the remaining metabolites were imputed by the mean of the given metabolites. All metabolite concentrations were then stanaderdized using the Z-score. The standardized concentrations of the metabolites detected by both p180 kit and the TMIC assay in the TASOAC were compared with the Mann-Whitney U test to examine the detection

differences of the two assays. One hundred twenty-nine (129) metabolites passed QC for each profiling assay and 67 metabolites overlapped between the two assays. For these 67 metabolites, all 3397 individuals were included for the analyses. For the 62 metabolites included in the p180 assay only, the 409 TASOAC samples profiled with TMIC assay were excluded from the analyses. In linear and logistic regression analysis, blood lipids, HOMA-IR, and visceral fat were not adjusted as data on blood lipids were only available for the CODING and NFOAS cohorts, and those on HOMA-IR and visceral fat were only available for the CODING cohort. Adjusting for these variables in selective cohorts might cause potential biases and influence the precision of the summary effect size in the meta-analysis [19]. To shed lights on the impact of osteoarthritis on metabolomics profile, BMI and obesity were also regressed on each standardized metabolite concentrations without adjustment for OA, and then meta-analysis was performed.

Supplementary results

Risk of bias assessment

The traffic light plot in **Figure S7** presents the results of risk of bias assessment.

Detection difference of the p180 kit and the TMIC assay

Among the 114 metabolites that overlap between the p180 kit and the TMIC assay, 91 passed QC, and the standardized concentrations of these metabolites measured with two different assays in the TOSOAC were not significantly different (all $p \geq 0.1$).

BMI/obesity associated metabolites

Out of the 66 metabolites significantly associated with BMI, 11 were amino acids, 2 were acylcarnitines, and 53 were phospholipids. Valine, isoleucine, alanine, glutamate, phenylalanine, tyrosine, kynurenine, and C0 and C3 acylcarnitines were positively associated with BMI, while glycine, serine, asparagine, and glutamine were negatively associated. One phospholipid species, acyl-alkyl-phosphatidylcholine with 38 carbons and 3 double bonds (PC aa C38:3), was positively associated with BMI while the other 52 species were negatively associated.

Out of the 63 metabolites significantly associated with obesity, 12 were amino acids, 2 were acylcarnitines, and 49 were phospholipids. Valine, isoleucine, leucine, alanine, glutamate, phenylalanine, tyrosine, kynurenine, and C0 and C3 acylcarnitines were positively associated with obesity, while glycine, serine, asparagine, and citrulline were negatively associated. Three phospholipid species, PC.aa.C32.1, PC aa C38:3, and PC aa C38:4, were positively associated with obesity while the other 46 species were negatively associated.

The forest plots of **Figure S1** present the results of all significant metabolites for BMI. The forest plots of **Figure S2** show the results of all other significant metabolites for obesity.

Valine, isoleucine, alanine, glutamate, phenylalanine, tyrosine, kynurenine, and C0 and C3 acylcarnitines were positively associated with both BMI and obesity, while glycine, serine, and asparagine were negatively associated. One phospholipid species, PC aa C38:3 was positively associated with BMI and obesity while 45 were negatively associated.

Figure S3 presents the PC metabolic pathway. We found the concentrations of several lysophosphatidylcholines (lysoPCs) were reduced in obesity or negatively associated with BMI, suggesting hydrolysis of PCs to lysoPCs by phospholipase A2 (PLA_2) was unlikely to be the

potential degradative pathway to explain our findings, because lysoPC levels would have been increased if this pathway was overactivated. However, based on data from the CODING and NFOAS cohorts, we found the majority of PCs were negatively correlated with TG (**Table S5, S6**), suggesting the degradative pathway of PCs by phospholipases C (PLC) may be overactivated in obesity, leading to an increased TG level in obesity (**Figure S3**). Whether the biosynthesis of PCs is reduced in obesity needs to be studied.

Impact of osteoarthritis status on metabolites associated with BMI/obesity

Without adjustment for OA status, citrulline and PC aa C34:2 were found to be significantly associated with BMI in addition to the 66 metabolites identified with adjustment for OA; two phospholipid species associated with obesity with adjustment for OA, PC aa C32:1 and PC ae C40:3, became non-significant, while PC aa C40:4 became significantly associated with obesity.

Relationships between BMI and obesity associated metabolites and diabetes, and levels of serum glucose, insulin and HOMA-IR

TASOAC-p180 was not included in this analysis as only 2 participants in this group had diabetes, hence, we had no statistical power to analyze this group.

In CODING cohort, data on serum glucose was available for 224 participants and those on insulin and HOMA-IR levels were available for 163. All three levels were significantly higher in obese group (all $p<0.001$, **Table S9**). Among the 58 metabolites significantly associated with both BMI and obesity, 25 PCs were negatively associated with diabetes in CODING, LEAP-OA, and NFOAS cohorts, out of which 17 were also negatively associated with HOMA-IR level ($p \leq 0.04$, **Table S1~S4**), suggesting that phospholipid metabolic pathways was associated with not only obesity, but also diabetes and insulin resistance. Although not associated with diabetes (**Table S1, S2**), valine, glutamate and C3 were positively associated with glucose, insulin and HOMA-IR levels, and glycine was negatively associated (all $p \leq 0.04$, **Table S3, S4**).

Relationships between BMI and obesity associated metabolites and blood lipids profile

Two hundred twenty-four (224) participants in CODING cohort and 382 in NFOAS cohort had data on conventional blood lipids profile. The levels in obese and non-obese groups are presented in **Table S9**. The majority of the phospholipid species associated with BMI and obesity were positively correlated with TC, HDL and LDL while negatively associated with VLDL and TG (all $p<0.04$, **Table S5, S6**).

Diet restriction trial in mice

All diets (Research Diets, New Brunswick, USA) had the same caloric density. Details of the diet formula are provided in **Table S12**. One mouse was ill and withdrawn from the trial.

Restriction of BCAAs or both BCAAs and phenylalanine within a standard chow diet (SCD) did not affect mouse body weight following 16-week feeding. However, the restriction of BCAAs, phenylalanine, and tryptophan together within a SCD significantly limited the mice's body weight gain compared to the SCD (**Figure S8A**) but had no effects on serum glucose or insulin levels, or insulin sensitivity (**Figure S8B-E**).

Supplementary tables

Table S1. Associations between standardized BMI-associated metabolite concentrations and diabetes

	CODING (n=226)		LEAP-OA (n=488)		Licofelone /naproxen clinical trial (n=158)		NFOAS (n=662)		TASOAC-TMIC (n=409)	
	**OR (95% CI)**	***p* value**	**OR (95% CI)**	***p* value**	**OR (95% CI)**	***p* value**	**OR (95% CI)**	***p* value**	**OR (95% CI)**	***p* value**
Alanine	1.13 (0.68-1.84)	0.62	2.03 (1.55-2.69)	**<0.001**	2.36 (1.36-4.33)	**0.003**	1.29 (1.04-1.59)	**0.02**	1.10 (0.52-2.22)	0.79
Asparagine	0.97 (0.40-2.01)	0.95	1.28 (0.98-1.65)	0.07	0.81 (0.38-1.51)	0.54	0.93 (0.75-1.15)	0.49	0.82 (0.34-1.75)	0.64
Glutamate	1.01 (0.60-1.62)	0.97	1.63 (1.24-2.15)	**<0.001**	1.05 (0.37-2.52)	0.91	1.14 (0.91-1.43)	0.24	1.93 (0.93-3.94)	0.07
Glutamine	0.96 (0.58-1.55)	0.87	0.82 (0.62-1.08)	0.17	1.19 (0.63-2.36)	0.60	0.90 (0.73-1.12)	0.36	1.08 (0.51-2.17)	0.84
Glycine	0.65 (0.37-1.05)	0.09	0.90 (0.65-1.23)	0.53	0.80 (0.37-1.59)	0.55	0.88 (0.67-1.14)	0.35	0.38 (0.11-1.04)	0.09
Isoleucine	1.81 (1.07-3.09)	**0.03**	1.58 (1.19-2.11)	**0.002**	0.97 (0.47-1.77)	0.92	1.71 (1.37-2.14)	**<0.001**	2.13 (1.04-4.45)	**0.04**
Kynurenine	0.84 (0.45-1.43)	0.55	1.16 (0.89-1.51)	0.26	1.71 (0.89-3.27)	0.10	0.84 (0.66-1.05)	0.13	1.11 (0.49-2.29)	0.79
Phenylalanine	0.71 (0.36-1.27)	0.28	0.99 (0.74-1.32)	0.97	1.02 (0.53-1.89)	0.95	1.09 (0.88-1.35)	0.43	2.36 (1.14-4.91)	**0.02**
Serine	0.82 (0.44-1.35)	0.47	0.93 (0.69-1.24)	0.63	0.67 (0.32-1.29)	0.26	1.09 (0.87-1.35)	0.44	0.50 (0.20-1.11)	0.11
Tyrosine	0.83 (0.48-1.38)	0.49	0.92 (0.68-1.23)	0.60	0.74 (0.37-1.35)	0.36	0.85 (0.67-1.06)	0.16	1.37 (0.68-2.62)	0.35
Valine	1.56 (0.99-2.51)	0.06	1.38 (1.04-1.83)	**0.03**	1.28 (0.67-2.32)	0.43	1.72 (1.38-2.15)	**<0.001**	2.60 (1.30-5.32)	**0.01**
C0	0.58 (0.35-0.92)	**0.02**	0.70 (0.52-0.93)	**0.02**	1.09 (0.57-2.10)	0.79	0.75 (0.61-0.92)	**0.006**	2.33 (1.15-4.84)	**0.02**
C3	1.18 (0.68-2.00)	0.54	1.28 (0.97-1.68)	0.07	1.62 (0.87-3.02)	0.12	1.20 (0.94-1.52)	0.14	1.38 (0.64-2.79)	0.39
LysoPC a C16:0	1.11 (0.75-1.62)	0.58	0.49 (0.35-0.66)	**<0.001**	0.98 (0.50-1.84)	0.95	0.71 (0.54-0.91)	**0.01**	0.63 (0.26-1.38)	0.28
LysoPC a C17:0	0.97 (0.62-1.45)	0.88	0.57 (0.41-0.78)	**<0.001**	0.85 (0.44-1.55)	0.61	0.74 (0.56-0.95)	**0.02**	0.50 (0.17-1.22)	0.16
LysoPC a C18:0	0.93 (0.60-1.41)	0.75	0.44 (0.31-0.61)	**<0.001**	0.90 (0.48-1.60)	0.72	0.66 (0.49-0.86)	**0.004**	0.78 (0.33-1.62)	0.53
LysoPC a C18:1	1.12 (0.75-1.65)	0.56	0.64 (0.46-0.86)	**0.004**	0.76 (0.35-1.57)	0.48	0.64 (0.49-0.82)	**<0.001**	0.65 (0.23-1.53)	0.37
LysoPC a C18:2	0.92 (0.59-1.38)	0.71	0.60 (0.43-0.82)	**0.002**	0.96 (0.50-1.71)	0.88	0.60 (0.46-0.78)	**<0.001**	0.37 (0.11-0.98)	0.07
LysoPC a C20:3	0.90 (0.58-1.34)	0.64	0.62 (0.45-0.82)	**0.002**	1.65 (0.82-3.41)	0.16	0.71 (0.55-0.91)	**0.007**	1.00 (0.41-2.13)	0.99
LysoPC a C20:4	1.36 (0.87-2.12)	0.17	1.01 (0.76-1.32)	0.97	0.92 (0.46-1.66)	0.80	0.91 (0.72-1.14)	0.42	1.48 (0.70-2.94)	0.27
PC aa C30:0	0.40 (0.21-0.72)	**0.004**	0.46 (0.31-0.66)	**<0.001**	1.00 (0.48-1.91)	0.99	0.75 (0.59-0.95)	**0.02**	-	-
PC aa C32:0	0.38 (0.21-0.66)	**0.001**	0.41 (0.28-0.58)	**<0.001**	0.84 (0.39-1.68)	0.64	0.64 (0.48-0.82)	**<0.001**	-	-
PC aa C36:6	0.47 (0.26-0.79)	**0.007**	0.56 (0.37-0.82)	**0.004**	0.84 (0.38-1.61)	0.64	0.54 (0.41-0.69)	**<0.001**	0.64 (0.22-1.56)	0.37
PC aa C38:0	0.66 (0.42-1.00)	0.06	0.46 (0.31-0.66)	**<0.001**	0.98 (0.47-1.95)	0.96	0.71 (0.55-0.90)	**0.005**	1.13 (0.48-2.33)	0.76
PC aa C38:3	0.43 (0.23-0.76)	**0.006**	0.42 (0.28-0.60)	**<0.001**	1.44 (0.79-2.60)	0.23	0.66 (0.51-0.83)	**<0.001**	-	-
PC aa C38:5	0.76 (0.49-1.16)	0.22	0.70 (0.50-0.96)	**0.03**	0.84 (0.45-1.51)	0.58	0.70 (0.55-0.86)	**0.001**	-	-
PC aa C38:6	0.62 (0.39-0.96)	**0.04**	0.63 (0.44-0.87)	**0.007**	0.94 (0.47-1.76)	0.85	0.70 (0.55-0.88)	**0.003**	0.79 (0.30-1.76)	0.59

PC aa C40:1	0.46 (0.22-0.85)	**0.02**	0.71 (0.52-0.95)	**0.03**	1.51 (0.68-3.10)	0.28	0.83 (0.66-1.04)	0.11	0.81 (0.31-1.79)	0.62
PC aa C40:2	0.29 (0.11-0.66)	**0.006**	0.57 (0.42-0.77)	**<0.001**	1.52 (0.67-2.97)	0.25	1.01 (0.80-1.25)	0.96	0.55 (0.20-1.32)	0.21
PC aa C40:3	0.30 (0.13-0.63)	**0.003**	0.46 (0.33-0.63)	**<0.001**	1.50 (0.64-3.07)	0.29	0.66 (0.51-0.83)	**<0.001**	-	-
PC aa C42:0	0.72 (0.46-1.11)	0.15	0.49 (0.33-0.71)	**<0.001**	1.13 (0.57-2.15)	0.72	0.75 (0.59-0.95)	**0.02**	-	-
PC aa C42:1	0.56 (0.34-0.88)	**0.02**	0.51 (0.36-0.72)	**<0.001**	1.22 (0.59-2.44)	0.57	0.73 (0.57-0.93)	**0.01**	-	-
PC aa C42:2	0.64 (0.38-1.00)	0.06	0.54 (0.38-0.74)	**<0.001**	1.10 (0.49-2.18)	0.79	0.63 (0.47-0.83)	**0.001**	-	-
PC aa C42:4	0.61 (0.34-1.04)	0.08	0.53 (0.39-0.71)	**<0.001**	1.43 (0.67-2.68)	0.29	0.88 (0.70-1.09)	0.24	-	-
PC aa C42:5	0.60 (0.35-0.98)	0.05	0.52 (0.35-0.73)	**<0.001**	1.24 (0.65-2.21)	0.48	0.78 (0.61-0.97)	**0.03**	-	-
PC aa C42:6	0.57 (0.33-0.95)	**0.04**	0.63 (0.45-0.86)	**0.005**	0.99 (0.49-1.87)	0.96	0.73 (0.57-0.91)	**0.007**	-	-
PC ae C30:0	0.46 (0.26-0.78)	**0.006**	0.41 (0.27-0.59)	**<0.001**	0.67 (0.34-1.25)	0.23	0.70 (0.54-0.90)	**0.006**	-	-
PC ae C32:1	0.20 (0.09-0.41)	**<0.001**	0.22 (0.14-0.32)	**<0.001**	1.05 (0.54-1.97)	0.89	0.48 (0.37-0.62)	**<0.001**	-	-
PC ae C32:2	0.24 (0.10-0.53)	**0.001**	0.23 (0.15-0.34)	**<0.001**	0.76 (0.34-1.57)	0.48	0.33 (0.23-0.45)	**<0.001**	-	-
PC ae C34:0	0.48 (0.27-0.81)	**0.009**	0.47 (0.33-0.65)	**<0.001**	0.94 (0.47-1.77)	0.86	0.68 (0.52-0.88)	**0.004**	-	-
PC ae C34:1	0.40 (0.21-0.70)	**0.002**	0.40 (0.28-0.55)	**<0.001**	0.66 (0.32-1.29)	0.23	0.53 (0.40-0.68)	**<0.001**	-	-
PC ae C34:2	0.48 (0.27-0.81)	**0.008**	0.25 (0.16-0.38)	**<0.001**	0.86 (0.43-1.64)	0.65	0.46 (0.34-0.60)	**<0.001**	-	-
PC ae C34:3	0.39 (0.21-0.68)	**0.002**	0.34 (0.23-0.50)	**<0.001**	0.87 (0.43-1.63)	0.67	0.46 (0.34-0.61)	**<0.001**	-	-
PC ae C36:0	0.57 (0.34-0.89)	**0.02**	0.63 (0.45-0.86)	**0.006**	0.96 (0.41-1.96)	0.91	0.75 (0.57-0.98)	**0.04**	1.13 (0.52-2.30)	0.74
PC ae C36:1	0.59 (0.34-0.96)	0.05	0.54 (0.39-0.72)	**<0.001**	1.36 (0.66-2.66)	0.38	0.88 (0.71-1.09)	0.24	-	-
PC ae C36:2	0.58 (0.34-0.93)	**0.03**	0.41 (0.29-0.58)	**<0.001**	1.15 (0.61-2.09)	0.66	0.60 (0.47-0.76)	**<0.001**	-	-
PC ae C36:3	0.49 (0.28-0.81)	**0.008**	0.28 (0.18-0.41)	**<0.001**	0.93 (0.48-1.72)	0.82	0.45 (0.34-0.59)	**<0.001**	-	-
PC ae C38:0	0.62 (0.36-1.03)	0.07	0.45 (0.30-0.65)	**<0.001**	1.05 (0.43-2.18)	0.91	0.52 (0.41-0.66)	**<0.001**	-	-
PC ae C38:4	0.78 (0.48-1.23)	0.30	0.59 (0.43-0.79)	**<0.001**	1.11 (0.58-2.11)	0.74	0.67 (0.53-0.83)	**<0.001**	-	-
PC ae C38:5	0.66 (0.40-1.05)	0.09	0.43 (0.30-0.60)	**<0.001**	0.91 (0.47-1.66)	0.76	0.55 (0.43-0.71)	**<0.001**	-	-
PC ae C38:6	0.66 (0.42-1.00)	0.06	0.32 (0.20-0.48)	**<0.001**	0.80 (0.37-1.56)	0.54	0.54 (0.42-0.70)	**<0.001**	-	-
PC ae C40:1	0.49 (0.29-0.78)	**0.005**	0.54 (0.38-0.73)	**<0.001**	1.06 (0.51-2.10)	0.87	0.56 (0.43-0.72)	**<0.001**	-	-
PC ae C40:2	0.37 (0.18-0.68)	**0.003**	0.42 (0.29-0.58)	**<0.001**	1.26 (0.57-2.60)	0.55	0.60 (0.46-0.76)	**<0.001**	-	-
PC ae C40:4	0.68 (0.41-1.13)	0.14	0.49 (0.36-0.66)	**<0.001**	1.35 (0.63-2.56)	0.39	0.74 (0.59-0.92)	**0.008**	-	-
PC ae C40:5	0.82 (0.52-1.25)	0.36	0.53 (0.38-0.72)	**<0.001**	1.25 (0.54-2.53)	0.56	0.62 (0.49-0.78)	**<0.001**	-	-
PC ae C40:6	0.61 (0.38-0.95)	**0.03**	0.47 (0.32-0.67)	**<0.001**	0.94 (0.44-1.92)	0.87	0.61 (0.48-0.77)	**<0.001**	0.86 (0.34-1.86)	0.72
PC ae C42:0	0.92 (0.57-1.48)	0.74	0.90 (0.66-1.20)	0.47	1.03 (0.52-1.92)	0.94	1.11 (0.90-1.36)	0.31	-	-
PC ae C42:1	0.35 (0.18-0.62)	**<0.001**	0.48 (0.35-0.66)	**<0.001**	1.11 (0.41-2.53)	0.81	0.57 (0.44-0.74)	**<0.001**	-	-
PC ae C42:2	0.36 (0.18-0.65)	**0.001**	0.40 (0.28-0.56)	**<0.001**	1.26 (0.62-2.39)	0.49	0.54 (0.42-0.70)	**<0.001**	-	-

PC ae C42:3	0.39 (0.22-0.66)	**<0.001**	0.45 (0.32-0.63)	**<0.001**	1.15 (0.56-2.24)	0.68	0.56 (0.43-0.72)	**<0.001**	-	-
PC ae C42:4	0.56 (0.32-0.93)	**0.03**	0.52 (0.38-0.69)	**<0.001**	1.36 (0.73-2.40)	0.30	0.68 (0.54-0.85)	**<0.001**	-	-
PC ae C42:5	0.83 (0.53-1.29)	0.42	0.52 (0.37-0.71)	**<0.001**	1.08 (0.54-1.98)	0.81	0.67 (0.53-0.85)	**<0.001**	-	-
PC ae C44:3	0.50 (0.27-0.88)	**0.02**	0.54 (0.39-0.73)	**<0.001**	1.60 (0.75-3.13)	0.18	0.65 (0.51-0.82)	**<0.001**	-	-
PC ae C44:4	0.58 (0.33-0.96)	**0.04**	0.55 (0.40-0.74)	**<0.001**	1.31 (0.74-2.26)	0.34	0.70 (0.55-0.88)	**0.003**	-	-
PC ae C44:5	0.87 (0.55-1.33)	0.52	0.58 (0.42-0.79)	**<0.001**	0.97 (0.51-1.73)	0.91	0.70 (0.55-0.87)	**0.002**	-	-
PC ae C44:6	0.81 (0.51-1.26)	0.37	0.60 (0.43-0.83)	**0.002**	1.02 (0.52-1.91)	0.96	0.81 (0.64-1.01)	0.07	-	-

BMI: body mass index; CODING: the Complex Diseases in the Newfoundland population: Environment and Genetics; LEAP-OA: Longitudinal Evaluation in the Arthritis Program, Osteoarthritis Study; NFOAS: Newfoundland Osteoarthritis Study; TASOAC-TMIC: Tasmanian Older Adult Cohort profiled with the TMIC Prime Metabolomics Profiling Assay; a: acyl; aa: acyl-acyl; ae: acyl-alkyl; C0: carnitine; C3: propionylcarnitine; Cx:y: where x is the number of carbons in the fatty acid side chain; y is the number of double bonds in the fatty acid side chain; PC: phophatidylcholine; OR: odds ratio; CI: confidence interval.

P values were obtained by multivariable logistic regression adjusting for age and sex.

Table S2. Associations between standardized obesity-associated metabolite concentrations and diabetes

	CODING (n=226)		LEAP-OA (n=488)		Licofelone /naproxen clinical trial (n=158)		NFOAS (n=662)		TASOAC-TMIC (n=409)	
	**OR (95% CI)**	***p* value**	**OR (95% CI)**	***p* value**	**OR (95% CI)**	***p* value**	**OR (95% CI)**	***p* value**	**OR (95% CI)**	***p* value**
Alanine	1.13 (0.68-1.84)	0.62	2.03 (1.55-2.69)	**<0.001**	2.36 (1.36-4.33)	**0.003**	1.29 (1.04-1.59)	**0.02**	1.10 (0.52-2.22)	0.79
Asparagine	0.97 (0.40-2.01)	0.95	1.28 (0.98-1.65)	0.07	0.81 (0.38-1.51)	0.54	0.93 (0.75-1.15)	0.49	0.82 (0.34-1.75)	0.64
Citrulline	0.59 (0.27-1.13)	0.14	0.30 (0.20-0.42)	**<0.001**	0.48 (0.23-0.92)	0.04	0.61 (0.47-0.78)	**<0.001**	0.63 (0.23-1.59)	0.35
Glutamate	1.01 (0.60-1.62)	0.97	1.63 (1.24-2.15)	**<0.001**	1.05 (0.37-2.52)	0.91	1.14 (0.91-1.43)	0.24	1.93 (0.93-3.94)	0.07
Glycine	0.65 (0.37-1.05)	0.09	0.90 (0.65-1.23)	0.53	0.80 (0.37-1.59)	0.55	0.88 (0.67-1.14)	0.35	0.38 (0.11-1.04)	0.09
Isoleucine	1.81 (1.07-3.09)	**0.03**	1.58 (1.19-2.11)	**0.002**	0.97 (0.47-1.77)	0.92	1.71 (1.37-2.14)	**<0.001**	2.13 (1.04-4.45)	**0.04**
Kynurenine	0.84 (0.45-1.43)	0.55	1.16 (0.89-1.51)	0.26	1.71 (0.89-3.27)	0.10	0.84 (0.66-1.05)	0.13	1.11 (0.49-2.29)	0.79
Leucine	0.74 (0.35-1.33)	0.37	1.40 (1.03-1.88)	**0.03**	0.95 (0.47-1.72)	0.88	1.46 (1.18-1.80)	**<0.001**	2.13 (1.00-4.54)	0.05
Phenylalanine	0.71 (0.36-1.27)	0.28	0.99 (0.74-1.32)	0.97	1.02 (0.53-1.89)	0.95	1.09 (0.88-1.35)	0.43	2.36 (1.14-4.91)	**0.02**
Serine	0.82 (0.44-1.35)	0.47	0.93 (0.69-1.24)	0.63	0.67 (0.32-1.29)	0.26	1.09 (0.87-1.35)	0.44	0.50 (0.20-1.11)	0.11
Tyrosine	0.83 (0.48-1.38)	0.49	0.92 (0.68-1.23)	0.60	0.74 (0.37-1.35)	0.36	0.85 (0.67-1.06)	0.16	1.37 (0.68-2.62)	0.35
Valine	1.56 (0.99-2.51)	0.06	1.38 (1.04-1.83)	**0.03**	1.28 (0.67-2.32)	0.43	1.72 (1.38-2.15)	**<0.001**	2.60 (1.30-5.32)	**0.01**
C0	0.58 (0.35-0.92)	**0.02**	0.70 (0.52-0.93)	**0.02**	1.09 (0.57-2.10)	0.79	0.75 (0.61-0.92)	**0.006**	2.33 (1.15-4.84)	**0.02**
C3	1.18 (0.68-2.00)	0.54	1.28 (0.97-1.68)	0.07	1.62 (0.87-3.02)	0.12	1.20 (0.94-1.52)	0.14	1.38 (0.64-2.79)	0.39
LysoPC a C16:0	1.11 (0.75-1.62)	0.58	0.49 (0.35-0.66)	**<0.001**	0.98 (0.50-1.84)	0.95	0.71 (0.54-0.91)	**0.01**	0.63 (0.26-1.38)	0.28
LysoPC a C17:0	0.97 (0.62-1.45)	0.88	0.57 (0.41-0.78)	**<0.001**	0.85 (0.44-1.55)	0.61	0.74 (0.56-0.95)	**0.02**	0.50 (0.17-1.22)	0.16
LysoPC a C18:0	0.93 (0.60-1.41)	0.75	0.44 (0.31-0.61)	**<0.001**	0.90 (0.48-1.60)	0.72	0.66 (0.49-0.86)	**0.004**	0.78 (0.33-1.62)	0.53
LysoPC a C18:1	1.12 (0.75-1.65)	0.56	0.64 (0.46-0.86)	**0.004**	0.76 (0.35-1.57)	0.48	0.64 (0.49-0.82)	**<0.001**	0.65 (0.23-1.53)	0.37
LysoPC a C18:2	0.92 (0.59-1.38)	0.71	0.60 (0.43-0.82)	**0.002**	0.96 (0.50-1.71)	0.88	0.60 (0.46-0.78)	**<0.001**	0.37 (0.11-0.98)	0.07
LysoPC a C20:4	1.36 (0.87-2.12)	0.17	1.01 (0.76-1.32)	0.97	0.92 (0.46-1.66)	0.80	0.91 (0.72-1.14)	0.42	1.48 (0.70-2.94)	0.27
PC aa C32:0	0.38 (0.21-0.66)	**0.001**	0.41 (0.28-0.58)	**<0.001**	0.84 (0.39-1.68)	0.64	0.64 (0.48-0.82)	**<0.001**	-	-
PC aa C32:1	0.62 (0.35-1.04)	0.09	0.59 (0.39-0.87)	**0.01**	1.10 (0.46-2.28)	0.81	0.71 (0.54-0.91)	**0.008**	-	-
PC aa C38:0	0.66 (0.42-1.00)	0.06	0.46 (0.31-0.66)	**<0.001**	0.98 (0.47-1.95)	0.96	0.71 (0.55-0.90)	**0.005**	1.13 (0.48-2.33)	0.76
PC aa C38:3	0.43 (0.23-0.76)	**0.006**	0.42 (0.28-0.60)	**<0.001**	1.44 (0.79-2.60)	0.23	0.66 (0.51-0.83)	**<0.001**	-	-
PC aa C38:4	0.88 (0.56-1.34)	0.56	0.93 (0.71-1.21)	0.60	1.25 (0.68-2.30)	0.46	0.93 (0.74-1.15)	0.50	-	-
PC aa C38:6	0.62 (0.39-0.96)	**0.04**	0.63 (0.44-0.87)	**0.007**	0.94 (0.47-1.76)	0.85	0.70 (0.55-0.88)	**0.003**	0.79 (0.30-1.76)	0.59
PC aa C40:1	0.46 (0.22-0.85)	**0.02**	0.71 (0.52-0.95)	**0.03**	1.51 (0.68-3.10)	0.28	0.83 (0.66-1.04)	0.11	0.81 (0.31-1.79)	0.62
PC aa C40:2	0.29 (0.11-0.66)	**0.006**	0.57 (0.42-0.77)	**<0.001**	1.52 (0.67-2.97)	0.25	1.01 (0.80-1.25)	0.96	0.55 (0.20-1.32)	0.21

PC aa C40:3	0.30 (0.13-0.63)	**0.003**	0.46 (0.33-0.63)	**<0.001**	1.50 (0.64-3.07)	0.29	0.66 (0.51-0.83)	**<0.001**	-	-
PC aa C42:0	0.72 (0.46-1.11)	0.15	0.49 (0.33-0.71)	**<0.001**	1.13 (0.57-2.15)	0.72	0.75 (0.59-0.95)	**0.02**	-	-
PC aa C42:1	0.56 (0.34-0.88)	**0.02**	0.51 (0.36-0.72)	**<0.001**	1.22 (0.59-2.44)	0.57	0.73 (0.57-0.93)	**0.01**	-	-
PC aa C42:2	0.64 (0.38-1.00)	0.06	0.54 (0.38-0.74)	**<0.001**	1.10 (0.49-2.18)	0.79	0.63 (0.47-0.83)	**0.001**	-	-
PC aa C42:4	0.61 (0.34-1.04)	0.08	0.53 (0.39-0.71)	**<0.001**	1.43 (0.67-2.68)	0.29	0.88 (0.70-1.09)	0.24	-	-
PC aa C42:5	0.60 (0.35-0.98)	0.05	0.52 (0.35-0.73)	**<0.001**	1.24 (0.65-2.21)	0.48	0.78 (0.61-0.97)	**0.03**	-	-
PC aa C42:6	0.57 (0.33-0.95)	**0.04**	0.63 (0.45-0.86)	**0.005**	0.99 (0.49-1.87)	0.96	0.73 (0.57-0.91)	**0.007**	-	-
PC ae C30:0	0.46 (0.26-0.78)	**0.006**	0.41 (0.27-0.59)	**<0.001**	0.67 (0.34-1.25)	0.23	0.70 (0.54-0.90)	**0.006**	-	-
PC ae C32:1	0.20 (0.09-0.41)	**<0.001**	0.22 (0.14-0.32)	**<0.001**	1.05 (0.54-1.97)	0.89	0.48 (0.37-0.62)	**<0.001**	-	-
PC ae C32:2	0.24 (0.10-0.53)	**0.001**	0.23 (0.15-0.34)	**<0.001**	0.76 (0.34-1.57)	0.48	0.33 (0.23-0.45)	**<0.001**	-	-
PC ae C34:0	0.48 (0.27-0.81)	**0.009**	0.47 (0.33-0.65)	**<0.001**	0.94 (0.47-1.77)	0.86	0.68 (0.52-0.88)	**0.004**	-	-
PC ae C34:1	0.40 (0.21-0.70)	**0.002**	0.40 (0.28-0.55)	**<0.001**	0.66 (0.32-1.29)	0.23	0.53 (0.40-0.68)	**<0.001**	-	-
PC ae C34:2	0.48 (0.27-0.81)	**0.008**	0.25 (0.16-0.38)	**<0.001**	0.86 (0.43-1.64)	0.65	0.46 (0.34-0.60)	**<0.001**	-	-
PC ae C34:3	0.39 (0.21-0.68)	**0.002**	0.34 (0.23-0.50)	**<0.001**	0.87 (0.43-1.63)	0.67	0.46 (0.34-0.61)	**<0.001**	-	-
PC ae C36:0	0.57 (0.34-0.89)	**0.02**	0.63 (0.45-0.86)	**0.006**	0.96 (0.41-1.96)	0.91	0.75 (0.57-0.98)	**0.04**	1.13 (0.52-2.30)	0.74
PC ae C36:1	0.59 (0.34-0.96)	0.05	0.54 (0.39-0.72)	**<0.001**	1.36 (0.66-2.66)	0.38	0.88 (0.71-1.09)	0.24	-	-
PC ae C36:2	0.58 (0.34-0.93)	**0.03**	0.41 (0.29-0.58)	**<0.001**	1.15 (0.61-2.09)	0.66	0.60 (0.47-0.76)	**<0.001**	-	-
PC ae C36:3	0.49 (0.28-0.81)	**0.008**	0.28 (0.18-0.41)	**<0.001**	0.93 (0.48-1.72)	0.82	0.45 (0.34-0.59)	**<0.001**	-	-
PC ae C38:0	0.62 (0.36-1.03)	0.07	0.45 (0.30-0.65)	**<0.001**	1.05 (0.43-2.18)	0.91	0.52 (0.41-0.66)	**<0.001**	-	-
PC ae C40:1	0.49 (0.29-0.78)	**0.005**	0.54 (0.38-0.73)	**<0.001**	1.06 (0.51-2.10)	0.87	0.56 (0.43-0.72)	**<0.001**	-	-
PC ae C40:2	0.37 (0.18-0.68)	**0.003**	0.42 (0.29-0.58)	**<0.001**	1.26 (0.57-2.60)	0.55	0.60 (0.46-0.76)	**<0.001**	-	-
PC ae C40:3	0.28 (0.13-0.56)	**<0.001**	0.44 (0.31-0.61)	**<0.001**	1.41 (0.68-2.58)	0.30	0.77 (0.61-0.96)	**0.03**	-	-
PC ae C40:4	0.68 (0.41-1.13)	0.14	0.49 (0.36-0.66)	**<0.001**	1.35 (0.63-2.56)	0.39	0.74 (0.59-0.92)	**0.008**	-	-
PC ae C40:5	0.82 (0.52-1.25)	0.36	0.53 (0.38-0.72)	**<0.001**	1.25 (0.54-2.53)	0.56	0.62 (0.49-0.78)	**<0.001**	-	-
PC ae C40:6	0.61 (0.38-0.95)	**0.03**	0.47 (0.32-0.67)	**<0.001**	0.94 (0.44-1.92)	0.87	0.61 (0.48-0.77)	**<0.001**	0.86 (0.34-1.86)	0.72
PC ae C42:0	0.92 (0.57-1.48)	0.74	0.90 (0.66-1.20)	0.47	1.03 (0.52-1.92)	0.94	1.11 (0.90-1.36)	0.31	-	-
PC ae C42:1	0.35 (0.18-0.62)	**<0.001**	0.48 (0.35-0.66)	**<0.001**	1.11 (0.41-2.53)	0.81	0.57 (0.44-0.74)	**<0.001**	-	-
PC ae C42:2	0.36 (0.18-0.65)	**0.001**	0.40 (0.28-0.56)	**<0.001**	1.26 (0.62-2.39)	0.49	0.54 (0.42-0.70)	**<0.001**	-	-
PC ae C42:3	0.39 (0.22-0.66)	**<0.001**	0.45 (0.32-0.63)	**<0.001**	1.15 (0.56-2.24)	0.68	0.56 (0.43-0.72)	**<0.001**	-	-
PC ae C42:4	0.56 (0.32-0.93)	**0.03**	0.52 (0.38-0.69)	**<0.001**	1.36 (0.73-2.40)	0.30	0.68 (0.54-0.85)	**<0.001**	-	-
PC ae C42:5	0.83 (0.53-1.29)	0.42	0.52 (0.37-0.71)	**<0.001**	1.08 (0.54-1.98)	0.81	0.67 (0.53-0.85)	**<0.001**	-	-
PC ae C44:3	0.50 (0.27-0.88)	**0.02**	0.54 (0.39-0.73)	**<0.001**	1.60 (0.75-3.13)	0.18	0.65 (0.51-0.82)	**<0.001**	-	-

PC ae C44:4	0.58 (0.33-0.96)	**0.04**	0.55 (0.40-0.74)	**<0.001**	1.31 (0.74-2.26)	0.34	0.70 (0.55-0.88)	**0.003**	-	-
PC ae C44:5	0.87 (0.55-1.33)	0.52	0.58 (0.42-0.79)	**<0.001**	0.97 (0.51-1.73)	0.91	0.70 (0.55-0.87)	**0.002**	-	-
PC ae C44:6	0.81 (0.51-1.26)	0.37	0.60 (0.43-0.83)	**0.002**	1.02 (0.52-1.91)	0.96	0.81 (0.64-1.01)	0.07	-	-

CODING: the Complex Diseases in the Newfoundland population: Environment and Genetics; LEAP-OA: Longitudinal Evaluation in the Arthritis Program, Osteoarthritis Study; NFOAS: Newfoundland Osteoarthritis Study; TASOAC-TMIC: Tasmanian Older Adult Cohort profiled with the TMIC Prime Metabolomics Profiling Assay; a: acyl; aa: acyl-acyl; ae: acyl-alkyl; C0: carnitine; C3: propionylcarnitine; Cx:y: where x is the number of carbons in the fatty acid side chain; y is the number of double bonds in the fatty acid side chain; PC: phophatidylcholine; OR: odds ratio; CI: confidence interval.

P values were obtained by multivariable logistic regression adjusting for age and sex.

Table S3. Association between standardized BMI-associated metabolite concentrations and serum glucose, insulin and HOMA-IR levels in the CODING cohort

Metabolite	Glucose (n=224)		Insulin (n=163)		HOMA-IR (n=163)	
	**Rho**	***p* value**	**Rho**	***p* value**	**Rho**	***p* value**
Alanine	0.19	**0.005**	0.15	0.06	0.16	0.05
Asparagine	0.01	0.91	0.00	0.96	0.01	0.90
Glutamate	0.33	**<0.001**	0.20	**0.01**	0.22	**0.005**
Glutamine	0.04	0.60	0.03	0.70	0.00	0.98
Glycine	-0.14	**0.04**	-0.22	**0.006**	-0.27	**<0.001**
Isoleucine	0.17	**0.01**	0.22	**0.005**	0.22	**0.005**
Kynurenine	0.14	0.05	0.16	**0.04**	0.10	0.19
Phenylalanine	0.08	0.22	0.10	0.20	0.05	0.56
Serine	-0.22	**0.001**	-0.08	0.30	-0.13	0.11
Tyrosine	0.14	0.05	0.12	0.13	0.07	0.42
Valine	0.25	**<0.001**	0.25	**0.001**	0.24	**0.003**
C0	0.24	**<0.001**	0.29	**<0.001**	0.27	**<0.001**
C3	0.33	**<0.001**	0.35	**<0.001**	0.36	**<0.001**
LysoPC a C16:0	0.16	**0.02**	0.07	0.38	0.09	0.28
LysoPC a C17:0	0.01	0.90	-0.10	0.21	-0.08	0.29
LysoPC a C18:0	0.11	0.11	0.04	0.62	0.05	0.51
LysoPC a C18:1	0.09	0.18	-0.04	0.64	0.01	0.94
LysoPC a C18:2	0.01	0.87	-0.04	0.61	-0.02	0.82
LysoPC a C20:3	0.11	0.09	0.17	**0.04**	0.19	**0.01**
LysoPC a C20:4	0.16	**0.02**	-0.04	0.66	0.02	0.81
PC aa C30:0	-0.11	0.10	-0.03	0.73	-0.02	0.83
PC aa C32:0	-0.15	**0.03**	-0.03	0.72	-0.05	0.55
PC aa C36:6	0.08	0.22	0.06	0.44	0.09	0.24
PC aa C38:0	-0.06	0.36	-0.16	**0.04**	-0.16	0.05
PC aa C38:3	0.04	0.60	0.22	**0.005**	0.21	**0.007**
PC aa C38:5	0.11	0.09	0.00	0.99	0.05	0.57
PC aa C38:6	0.03	0.66	-0.02	0.81	-0.01	0.86
PC aa C40:1	-0.27	**<0.001**	-0.27	**<0.001**	-0.28	**<0.001**
PC aa C40:2	-0.32	**<0.001**	-0.21	**0.008**	-0.25	**0.002**
PC aa C40:3	-0.21	**0.002**	-0.14	0.08	-0.15	0.06
PC aa C42:0	-0.23	**<0.001**	-0.31	**<0.001**	-0.32	**<0.001**
PC aa C42:1	-0.28	**<0.001**	-0.37	**<0.001**	-0.38	**<0.001**
PC aa C42:2	-0.13	0.05	-0.17	**0.03**	-0.15	0.07
PC aa C42:4	-0.29	**<0.001**	-0.18	**0.02**	-0.19	**0.02**
PC aa C42:5	-0.12	0.09	-0.05	0.54	-0.04	0.58
PC aa C42:6	-0.13	0.06	-0.13	0.12	-0.09	0.27
PC ae C30:0	-0.20	**0.003**	-0.19	**0.02**	-0.19	**0.02**
PC ae C32:1	-0.37	**<0.001**	-0.20	**0.01**	-0.28	**<0.001**
PC ae C32:2	-0.34	**<0.001**	-0.22	**0.006**	-0.28	**<0.001**
PC ae C34:0	-0.09	0.18	-0.10	0.19	-0.12	0.12

PC ae C34:1	-0.29	**<0.001**	-0.16	0.05	-0.20	**0.01**
PC ae C34:2	-0.26	**<0.001**	-0.27	**<0.001**	-0.28	**<0.001**
PC ae C34:3	-0.33	**<0.001**	-0.29	**<0.001**	-0.33	**<0.001**
PC ae C36:0	-0.14	**0.04**	-0.09	0.28	-0.10	0.22
PC ae C36:1	-0.18	**0.007**	-0.11	0.18	-0.12	0.14
PC ae C36:2	-0.26	**<0.001**	-0.21	**0.008**	-0.24	**0.002**
PC ae C36:3	-0.27	**<0.001**	-0.24	**0.002**	-0.25	**0.002**
PC ae C38:0	0.14	0.05	-0.04	0.60	-0.01	0.92
PC ae C38:4	-0.25	**<0.001**	-0.20	**0.01**	-0.23	**0.003**
PC ae C38:5	-0.15	**0.03**	-0.17	**0.04**	-0.20	**0.01**
PC ae C38:6	-0.05	0.48	-0.17	**0.03**	-0.16	**0.04**
PC ae C40:1	-0.12	0.08	-0.13	0.11	-0.15	0.06
PC ae C40:2	-0.26	**<0.001**	-0.18	**0.03**	-0.17	**0.04**
PC ae C40:4	-0.32	**<0.001**	-0.29	**<0.001**	-0.31	**<0.001**
PC ae C40:5	-0.16	**0.02**	-0.21	**0.008**	-0.21	**0.007**
PC ae C40:6	-0.17	**0.01**	-0.31	**<0.001**	-0.29	**<0.001**
PC ae C42:0	0.09	0.18	-0.05	0.50	-0.05	0.55
PC ae C42:1	-0.23	**<0.001**	-0.14	0.09	-0.18	**0.02**
PC ae C42:2	-0.23	**<0.001**	-0.21	**0.009**	-0.21	**0.008**
PC ae C42:3	-0.32	**<0.001**	-0.30	**<0.001**	-0.33	**<0.001**
PC ae C42:4	-0.40	**<0.001**	-0.34	**<0.001**	-0.37	**<0.001**
PC ae C42:5	-0.28	**<0.001**	-0.32	**<0.001**	-0.33	**<0.001**
PC ae C44:3	-0.30	**<0.001**	-0.25	**0.001**	-0.26	**0.001**
PC ae C44:4	-0.35	**<0.001**	-0.26	**0.001**	-0.27	**<0.001**
PC ae C44:5	-0.32	**<0.001**	-0.38	**<0.001**	-0.40	**<0.001**
PC ae C44:6	-0.35	**<0.001**	-0.42	**<0.001**	-0.42	**<0.001**

BMI: body mass index; HOMA-IR: homeostatic model assessment for insulin resistance; CODING: the Complex Diseases in the Newfoundland population: Environment and Genetics; a: acyl; aa: acyl-acyl; ae: acyl-alkyl; C0: carnitine; C3: propionylcarnitine; Cx:y: where x is the number of carbons in the fatty acid side chain; y is the number of double bonds in the fatty acid side chain; PC: phophatidylcholine.

P values were obtained by Spearman's rank correlation.

Table S4. Association between standardized obesity-associated metabolite concentrations and serum glucose, insulin and HOMA-IR levels in the CODING cohort

Metabolite	Glucose (n=224)		Insulin (n=163)		HOMA-IR (n=163)	
	**Rho**	***p* value**	**Rho**	***p* value**	**Rho**	***p* value**
Alanine	0.19	**0.005**	0.15	0.06	0.16	0.05
Asparagine	0.01	0.91	0.00	0.96	0.01	0.90
Citrulline	0.01	0.91	-0.02	0.84	-0.02	0.76
Glutamate	0.33	**<0.001**	0.20	**0.01**	0.22	**0.005**
Glycine	-0.14	**0.04**	-0.22	**0.006**	-0.27	**<0.001**
Isoleucine	0.17	**0.01**	0.22	**0.005**	0.22	**0.005**
Kynurenine	0.14	0.05	0.16	**0.04**	0.10	0.19
Leucine	0.07	0.28	0.16	**0.04**	0.12	0.13
Phenylalanine	0.08	0.22	0.10	0.20	0.05	0.56
Serine	-0.22	**0.001**	-0.08	0.30	-0.13	0.11
Tyrosine	0.14	0.05	0.12	0.13	0.07	0.42
Valine	0.25	**<0.001**	0.25	**0.001**	0.24	**0.003**
C0	0.24	**<0.001**	0.29	**<0.001**	0.27	**<0.001**
C3	0.33	**<0.001**	0.35	**<0.001**	0.36	**<0.001**
LysoPC a C16:0	0.16	**0.02**	0.07	0.38	0.09	0.28
LysoPC a C17:0	0.01	0.90	-0.10	0.21	-0.08	0.29
LysoPC a C18:0	0.11	0.11	0.04	0.62	0.05	0.51
LysoPC a C18:1	0.09	0.18	-0.04	0.64	0.01	0.94
LysoPC a C18:2	0.01	0.87	-0.04	0.61	-0.02	0.82
LysoPC a C20:4	0.16	**0.02**	-0.04	0.66	0.02	0.81
PC aa C32:0	-0.15	**0.03**	-0.03	0.72	-0.05	0.55
PC aa C32:1	0.04	0.57	0.22	**0.004**	0.20	**0.01**
PC aa C38:0	-0.06	0.36	-0.16	**0.04**	-0.16	0.05
PC aa C38:3	0.04	0.60	0.22	**0.005**	0.21	**0.007**
PC aa C38:4	0.06	0.38	0.09	0.24	0.09	0.24
PC aa C38:6	0.03	0.66	-0.02	0.81	-0.01	0.86
PC aa C40:1	-0.27	**<0.001**	-0.27	**<0.001**	-0.28	**<0.001**
PC aa C40:2	-0.32	**<0.001**	-0.21	**0.008**	-0.25	**0.002**
PC aa C40:3	-0.21	**0.002**	-0.14	0.08	-0.15	0.06
PC aa C42:0	-0.23	**<0.001**	-0.31	**<0.001**	-0.32	**<0.001**
PC aa C42:1	-0.28	**<0.001**	-0.37	**<0.001**	-0.38	**<0.001**
PC aa C42:2	-0.13	0.05	-0.17	**0.03**	-0.15	0.07
PC aa C42:4	-0.29	**<0.001**	-0.18	**0.02**	-0.19	**0.02**
PC aa C42:5	-0.12	0.09	-0.05	0.54	-0.04	0.58
PC aa C42:6	-0.13	0.06	-0.13	0.12	-0.09	0.27
PC ae C30:0	-0.20	**0.003**	-0.19	**0.02**	-0.19	**0.02**
PC ae C32:1	-0.37	**<0.001**	-0.20	**0.01**	-0.28	**<0.001**
PC ae C32:2	-0.34	**<0.001**	-0.22	**0.006**	-0.28	**<0.001**
PC ae C34:0	-0.09	0.18	-0.10	0.19	-0.12	0.12
PC ae C34:1	-0.29	**<0.001**	-0.16	0.05	-0.20	**0.01**

PC ae C34:2	-0.26	**<0.001**	-0.27	**<0.001**	-0.28	**<0.001**
PC ae C34:3	-0.33	**<0.001**	-0.29	**<0.001**	-0.33	**<0.001**
PC ae C36:0	-0.14	**0.04**	-0.09	0.28	-0.10	0.22
PC ae C36:1	-0.18	**0.007**	-0.11	0.18	-0.12	0.14
PC ae C36:2	-0.26	**<0.001**	-0.21	**0.008**	-0.24	**0.002**
PC ae C36:3	-0.27	**<0.001**	-0.24	**0.002**	-0.25	**0.002**
PC ae C38:0	0.14	0.05	-0.04	0.60	-0.01	0.92
PC ae C40:1	-0.12	0.08	-0.13	0.11	-0.15	0.06
PC ae C40:2	-0.26	**<0.001**	-0.18	**0.03**	-0.17	**0.04**
PC ae C40:3	-0.39	**<0.001**	-0.34	**<0.001**	-0.37	**<0.001**
PC ae C40:4	-0.32	**<0.001**	-0.29	**<0.001**	-0.31	**<0.001**
PC ae C40:5	-0.16	**0.02**	-0.21	**0.008**	-0.21	**0.007**
PC ae C40:6	-0.17	**0.01**	-0.31	**<0.001**	-0.29	**<0.001**
PC ae C42:0	0.09	0.18	-0.05	0.50	-0.05	0.55
PC ae C42:1	-0.23	**<0.001**	-0.14	0.09	-0.18	**0.02**
PC ae C42:2	-0.23	**<0.001**	-0.21	**0.009**	-0.21	**0.008**
PC ae C42:3	-0.32	**<0.001**	-0.30	**<0.001**	-0.33	**<0.001**
PC ae C42:4	-0.40	**<0.001**	-0.34	**<0.001**	-0.37	**<0.001**
PC ae C42:5	-0.28	**<0.001**	-0.32	**<0.001**	-0.33	**<0.001**
PC ae C44:3	-0.30	**<0.001**	-0.25	**0.001**	-0.26	**0.001**
PC ae C44:4	-0.35	**<0.001**	-0.26	**0.001**	-0.27	**<0.001**
PC ae C44:5	-0.32	**<0.001**	-0.38	**<0.001**	-0.40	**<0.001**
PC ae C44:6	-0.35	**<0.001**	-0.42	**<0.001**	-0.42	**<0.001**

HOMA-IR: Homeostatic model assessment for insulin resistance; CODING: the Complex Diseases in the Newfoundland population: Environment and Genetics; a: acyl; aa: acyl-acyl; ae: acyl-alkyl; C0: carnitine; C3: propionylcarnitine; Cx:y: where x is the number of carbons in the fatty acid side chain; y is the number of double bonds in the fatty acid side chain; PC: phophatidylcholine.

P values were obtained by Spearman's rank correlation.

Table S5. Association between standardized BMI-associated metabolite concentrations and blood lipids profile in the CODING and NFOAS cohorts

Metabolites	Lipid (Ratio)	CODING (n=224)		NFOAS (n=382)	
		Rho	*p* value	Rho	*p* value
Alanine	TC	0.11	0.10	-0.03	0.51
	HDL	-0.16	**0.02**	-0.19	**<0.001**
	LDL	0.10	0.14	-0.08	0.14
	Non-HDL	0.12	0.07	0.00	0.97
	VLDL	0.28	**<0.001**	0.27	**<0.001**
	TG	0.28	**<0.001**	0.27	**<0.001**
	TC/HDL	0.17	**0.01**	0.14	**0.008**
Asparagine	TC	-0.02	0.76	-0.09	0.07
	HDL	0.03	0.61	-0.10	0.05
	LDL	-0.01	0.88	-0.06	0.23
	Non-HDL	-0.07	0.32	-0.08	0.11
	VLDL	0.01	0.88	-0.07	0.18
	TG	0.01	0.88	-0.07	0.18
	TC/HDL	-0.09	0.20	0.00	1.00
Glutamate	TC	0.07	0.29	-0.08	0.14
	HDL	-0.30	**<0.001**	-0.28	**<0.001**
	LDL	0.05	0.50	-0.12	**0.02**
	Non-HDL	0.19	**0.006**	-0.02	0.65
	VLDL	0.33	**<0.001**	0.30	**<0.001**
	TG	0.33	**<0.001**	0.30	**<0.001**
	TC/HDL	0.34	**<0.001**	0.14	**0.006**
Glutamine	TC	0.00	0.96	-0.03	0.59
	HDL	-0.05	0.49	-0.07	0.16
	LDL	0.08	0.24	0.00	0.99
	Non-HDL	0.02	0.80	-0.01	0.83
	VLDL	0.01	0.87	-0.04	0.41
	TG	0.01	0.88	-0.04	0.41
	TC/HDL	0.08	0.23	0.03	0.54
Glycine	TC	0.05	0.43	0.13	**0.01**
	HDL	0.26	**<0.001**	0.09	0.10
	LDL	0.09	0.17	0.14	**0.009**
	Non-HDL	-0.05	0.46	0.10	0.05
	VLDL	-0.16	**0.02**	-0.06	0.25
	TG	-0.16	**0.02**	-0.06	0.24
	TC/HDL	-0.18	**0.006**	0.00	0.98
Isoleucine	TC	0.01	0.91	-0.17	**<0.001**
	HDL	-0.35	**<0.001**	-0.38	**<0.001**
	LDL	0.01	0.85	-0.14	**0.007**
	Non-HDL	0.08	0.22	-0.05	0.37
	VLDL	0.31	**<0.001**	0.25	**<0.001**

	TG	0.31	**<0.001**	0.25	**<0.001**
	TC/HDL	0.26	**<0.001**	0.25	**<0.001**
Kynurenine	TC	0.04	0.56	-0.07	0.20
	HDL	-0.16	**0.02**	-0.04	0.40
	LDL	0.03	0.65	-0.08	0.13
	Non-HDL	0.11	0.11	-0.04	0.47
	VLDL	0.16	**0.02**	0.09	0.07
	TG	0.16	**0.02**	0.09	0.07
	TC/HDL	0.18	**0.007**	0.02	0.74
Phenylalanine	TC	0.12	0.06	-0.04	0.43
	HDL	-0.17	**0.01**	-0.20	**<0.001**
	LDL	0.09	0.20	-0.02	0.73
	Non-HDL	0.16	**0.02**	0.02	0.65
	VLDL	0.21	**0.002**	0.13	**0.01**
	TG	0.21	**0.002**	0.13	**0.01**
	TC/HDL	0.22	**<0.001**	0.15	**0.003**
Serine	TC	-0.03	0.64	0.03	0.55
	HDL	0.06	0.38	-0.10	0.06
	LDL	-0.01	0.85	0.08	0.12
	Non-HDL	-0.09	0.20	0.06	0.27
	VLDL	-0.10	0.14	-0.07	0.20
	TG	-0.10	0.13	-0.07	0.20
	TC/HDL	-0.08	0.24	0.11	**0.03**
Tyrosine	TC	0.14	**0.04**	-0.04	0.38
	HDL	-0.10	0.14	-0.14	**0.006**
	LDL	0.15	**0.03**	0.01	0.92
	Non-HDL	0.15	**0.02**	0.01	0.83
	VLDL	0.21	**0.002**	0.00	0.98
	TG	0.21	**0.002**	0.00	0.97
	TC/HDL	0.19	**0.004**	0.12	**0.02**
Valine	TC	0.17	**0.01**	-0.13	**0.01**
	HDL	-0.30	**<0.001**	-0.34	**<0.001**
	LDL	0.10	0.13	-0.09	0.07
	Non-HDL	0.26	**<0.001**	-0.01	0.77
	VLDL	0.38	**<0.001**	0.23	**<0.001**
	TG	0.38	**<0.001**	0.23	**<0.001**
	TC/HDL	0.35	**<0.001**	0.26	**<0.001**
C0	TC	0.20	**0.002**	-0.06	0.23
	HDL	-0.28	**<0.001**	-0.15	**0.005**
	LDL	0.18	**0.006**	-0.06	0.21
	Non-HDL	0.29	**<0.001**	-0.03	0.57
	VLDL	0.30	**<0.001**	0.08	0.10
	TG	0.30	**<0.001**	0.08	0.10
	TC/HDL	0.37	**<0.001**	0.05	0.30
C3	TC	0.12	0.09	-0.10	0.05

	HDL	-0.39	**<0.001**	-0.21	**<0.001**
	LDL	0.13	0.05	-0.11	**0.04**
	Non-HDL	0.23	**<0.001**	-0.05	0.35
	VLDL	0.39	**<0.001**	0.14	**0.005**
	TG	0.39	**<0.001**	0.15	**0.005**
	TC/HDL	0.40	**<0.001**	0.08	0.14
LysoPC a C16:0	TC	0.25	**<0.001**	0.25	**<0.001**
	HDL	-0.07	0.31	0.07	0.18
	LDL	0.19	**0.005**	0.21	**<0.001**
	Non-HDL	0.26	**<0.001**	0.25	**<0.001**
	VLDL	0.29	**<0.001**	0.16	**0.002**
	TG	0.29	**<0.001**	0.16	**0.002**
	TC/HDL	0.25	**<0.001**	0.15	**0.005**
LysoPC a C17:0	TC	0.17	**0.01**	0.16	**0.002**
	HDL	0.07	0.28	0.05	0.39
	LDL	0.16	**0.02**	0.13	**0.009**
	Non-HDL	0.14	**0.03**	0.15	**0.004**
	VLDL	0.03	0.68	0.05	0.35
	TG	0.03	0.69	0.05	0.36
	TC/HDL	0.07	0.33	0.07	0.19
LysoPC a C18:0	TC	0.29	**<0.001**	0.27	**<0.001**
	HDL	0.01	0.84	0.10	0.06
	LDL	0.26	**<0.001**	0.25	**<0.001**
	Non-HDL	0.28	**<0.001**	0.27	**<0.001**
	VLDL	0.17	**0.01**	0.11	**0.04**
	TG	0.16	**0.02**	0.11	**0.04**
	TC/HDL	0.21	**0.001**	0.13	**0.01**
LysoPC a C18:1	TC	0.17	**0.01**	0.21	**<0.001**
	HDL	0.06	0.39	0.16	**0.002**
	LDL	0.17	**0.01**	0.17	**<0.001**
	Non-HDL	0.14	**0.04**	0.18	**<0.001**
	VLDL	0.06	0.40	0.02	0.68
	TG	0.06	0.41	0.02	0.68
	TC/HDL	0.06	0.35	0.02	0.72
LysoPC a C18:2	TC	0.16	**0.02**	0.25	**<0.001**
	HDL	0.14	**0.04**	0.19	**<0.001**
	LDL	0.18	**0.008**	0.24	**<0.001**
	Non-HDL	0.10	0.16	0.21	**<0.001**
	VLDL	-0.04	0.51	-0.09	0.07
	TG	-0.05	0.50	-0.09	0.07
	TC/HDL	-0.02	0.82	0.03	0.56
LysoPC a C20:3	TC	0.26	**<0.001**	0.26	**<0.001**
	HDL	-0.11	0.10	0.16	**0.002**
	LDL	0.20	**0.002**	0.19	**<0.001**
	Non-HDL	0.26	**<0.001**	0.24	**<0.001**

	VLDL	0.33	**<0.001**	0.15	**0.005**
	TG	0.33	**<0.001**	0.15	**0.005**
	TC/HDL	0.24	**<0.001**	0.08	0.12
LysoPC a C20:4	TC	0.07	0.33	0.02	0.66
	HDL	-0.07	0.33	0.06	0.29
	LDL	0.05	0.42	-0.04	0.43
	Non-HDL	0.08	0.23	0.00	0.93
	VLDL	0.14	**0.04**	0.08	0.11
	TG	0.14	**0.04**	0.08	0.11
	TC/HDL	0.11	0.12	-0.05	0.32
PC aa C30:0	TC	0.31	**<0.001**	0.48	**<0.001**
	HDL	0.18	**0.006**	0.37	**<0.001**
	LDL	0.22	**0.001**	0.41	**<0.001**
	Non-HDL	0.21	**0.001**	0.41	**<0.001**
	VLDL	0.11	0.10	0.02	0.71
	TG	0.11	0.11	0.02	0.71
	TC/HDL	0.04	0.58	0.07	0.18
PC aa C32:0	TC	0.41	**<0.001**	0.48	**<0.001**
	HDL	0.15	**0.02**	0.26	**<0.001**
	LDL	0.30	**<0.001**	0.43	**<0.001**
	Non-HDL	0.32	**<0.001**	0.44	**<0.001**
	VLDL	0.11	0.09	0.10	0.06
	TG	0.11	0.10	0.10	0.06
	TC/HDL	0.13	0.05	0.19	**<0.001**
PC aa C36:6	TC	0.38	**<0.001**	0.37	**<0.001**
	HDL	0.14	**0.04**	0.30	**<0.001**
	LDL	0.25	**<0.001**	0.30	**<0.001**
	Non-HDL	0.32	**<0.001**	0.31	**<0.001**
	VLDL	0.29	**<0.001**	0.05	0.37
	TG	0.29	**<0.001**	0.05	0.36
	TC/HDL	0.12	0.09	0.03	0.52
PC aa C38:0	TC	0.34	**<0.001**	0.36	**<0.001**
	HDL	0.25	**<0.001**	0.26	**<0.001**
	LDL	0.29	**<0.001**	0.34	**<0.001**
	Non-HDL	0.25	**<0.001**	0.31	**<0.001**
	VLDL	-0.02	0.82	-0.07	0.21
	TG	-0.02	0.81	-0.07	0.21
	TC/HDL	0.05	0.48	0.07	0.17
PC aa C38:3	TC	0.39	**<0.001**	0.50	**<0.001**
	HDL	-0.08	0.22	0.24	**<0.001**
	LDL	0.28	**<0.001**	0.43	**<0.001**
	Non-HDL	0.40	**<0.001**	0.49	**<0.001**
	VLDL	0.43	**<0.001**	0.22	**<0.001**
	TG	0.43	**<0.001**	0.22	**<0.001**
	TC/HDL	0.32	**<0.001**	0.24	**<0.001**

PC aa C38:5	TC	0.42	**<0.001**	0.35	**<0.001**
	HDL	0.02	0.77	0.29	**<0.001**
	LDL	0.30	**<0.001**	0.24	**<0.001**
	Non-HDL	0.40	**<0.001**	0.29	**<0.001**
	VLDL	0.35	**<0.001**	0.16	**0.002**
	TG	0.35	**<0.001**	0.16	**0.002**
	TC/HDL	0.25	**<0.001**	0.03	0.60
PC aa C38:6	TC	0.33	**<0.001**	0.35	**<0.001**
	HDL	0.08	0.26	0.23	**<0.001**
	LDL	0.21	**0.002**	0.28	**<0.001**
	Non-HDL	0.31	**<0.001**	0.30	**<0.001**
	VLDL	0.29	**<0.001**	0.12	**0.02**
	TG	0.29	**<0.001**	0.12	**0.02**
	TC/HDL	0.18	**0.008**	0.08	0.12
PC aa C40:1	TC	0.16	**0.02**	0.27	**<0.001**
	HDL	0.25	**<0.001**	0.17	**<0.001**
	LDL	0.16	**0.02**	0.27	**<0.001**
	Non-HDL	0.07	0.28	0.24	**<0.001**
	VLDL	-0.15	**0.03**	-0.06	0.25
	TG	-0.15	**0.03**	-0.06	0.25
	TC/HDL	-0.07	0.31	0.09	0.08
PC aa C40:2	TC	0.12	0.07	0.23	**<0.001**
	HDL	0.25	**<0.001**	0.15	**0.004**
	LDL	0.13	0.05	0.22	**<0.001**
	Non-HDL	0.03	0.65	0.21	**<0.001**
	VLDL	-0.17	**0.01**	-0.02	0.66
	TG	-0.17	**0.01**	-0.02	0.66
	TC/HDL	-0.11	0.09	0.09	0.07
PC aa C40:3	TC	0.16	**0.02**	0.33	**<0.001**
	HDL	0.19	**0.004**	0.20	**<0.001**
	LDL	0.17	**0.01**	0.32	**<0.001**
	Non-HDL	0.09	0.20	0.30	**<0.001**
	VLDL	-0.09	0.19	-0.03	0.60
	TG	-0.09	0.19	-0.03	0.61
	TC/HDL	-0.04	0.54	0.11	**0.03**
PC aa C42:0	TC	0.17	**0.01**	0.26	**<0.001**
	HDL	0.24	**<0.001**	0.22	**<0.001**
	LDL	0.14	**0.03**	0.31	**<0.001**
	Non-HDL	0.07	0.29	0.23	**<0.001**
	VLDL	-0.18	**0.007**	-0.19	**<0.001**
	TG	-0.18	**0.006**	-0.19	**<0.001**
	TC/HDL	-0.09	0.20	0.03	0.56
PC aa C42:1	TC	0.20	**0.003**	0.27	**<0.001**
	HDL	0.32	**<0.001**	0.23	**<0.001**
	LDL	0.17	**0.01**	0.31	**<0.001**

	Non-HDL	0.07	0.31	0.23	**<0.001**
	VLDL	-0.21	**0.002**	-0.16	**0.002**
	TG	-0.21	**0.002**	-0.16	**0.002**
	TC/HDL	-0.14	**0.04**	0.05	0.30
PC aa C42:2	TC	0.24	**<0.001**	0.26	**<0.001**
	HDL	0.26	**<0.001**	0.19	**<0.001**
	LDL	0.18	**0.009**	0.28	**<0.001**
	Non-HDL	0.13	0.06	0.22	**<0.001**
	VLDL	-0.04	0.52	-0.17	**0.001**
	TG	-0.04	0.51	-0.17	**0.001**
	TC/HDL	-0.06	0.39	0.03	0.52
PC aa C42:4	TC	0.22	**0.001**	0.26	**<0.001**
	HDL	0.22	**0.001**	0.16	**0.001**
	LDL	0.18	**0.008**	0.25	**<0.001**
	Non-HDL	0.14	**0.04**	0.23	**<0.001**
	VLDL	-0.03	0.68	-0.02	0.68
	TG	-0.03	0.67	-0.02	0.68
	TC/HDL	-0.03	0.67	0.06	0.23
PC aa C42:5	TC	0.27	**<0.001**	0.31	**<0.001**
	HDL	0.07	0.27	0.16	**0.002**
	LDL	0.20	**0.003**	0.28	**<0.001**
	Non-HDL	0.23	**<0.001**	0.29	**<0.001**
	VLDL	0.12	0.09	0.08	0.14
	TG	0.12	0.09	0.08	0.13
	TC/HDL	0.09	0.19	0.12	**0.02**
PC aa C42:6	TC	0.22	**0.001**	0.24	**<0.001**
	HDL	0.22	**0.001**	0.19	**<0.001**
	LDL	0.16	**0.02**	0.19	**<0.001**
	Non-HDL	0.14	**0.04**	0.21	**<0.001**
	VLDL	0.07	0.28	0.05	0.35
	TG	0.07	0.29	0.05	0.34
	TC/HDL	-0.02	0.75	0.02	0.74
PC ae C30:0	TC	0.24	**<0.001**	0.38	**<0.001**
	HDL	0.30	**<0.001**	0.26	**<0.001**
	LDL	0.20	**0.003**	0.38	**<0.001**
	Non-HDL	0.13	0.05	0.33	**<0.001**
	VLDL	-0.10	0.13	-0.12	**0.02**
	TG	-0.11	0.12	-0.12	**0.02**
	TC/HDL	-0.09	0.21	0.08	0.10
PC ae C32:1	TC	0.16	**0.02**	0.43	**<0.001**
	HDL	0.32	**<0.001**	0.38	**<0.001**
	LDL	0.14	**0.04**	0.43	**<0.001**
	Non-HDL	0.03	0.66	0.37	**<0.001**
	VLDL	-0.26	**<0.001**	-0.16	**0.001**
	TG	-0.26	**<0.001**	-0.16	**0.001**

	TC/HDL	-0.15	**0.02**	0.07	0.19
PC ae C32:2	TC	0.16	**0.02**	0.43	**<0.001**
	HDL	0.32	**<0.001**	0.41	**<0.001**
	LDL	0.17	**0.01**	0.40	**<0.001**
	Non-HDL	0.03	0.66	0.35	**<0.001**
	VLDL	-0.21	**0.002**	-0.15	**0.003**
	TG	-0.21	**0.002**	-0.15	**0.003**
	TC/HDL	-0.16	**0.02**	0.02	0.74
PC ae C34:0	TC	0.37	**<0.001**	0.39	**<0.001**
	HDL	0.18	**0.007**	0.23	**<0.001**
	LDL	0.27	**<0.001**	0.37	**<0.001**
	Non-HDL	0.30	**<0.001**	0.36	**<0.001**
	VLDL	0.11	0.11	-0.02	0.73
	TG	0.11	0.12	-0.02	0.73
	TC/HDL	0.11	0.09	0.14	**0.006**
PC ae C34:1	TC	0.33	**<0.001**	0.45	**<0.001**
	HDL	0.27	**<0.001**	0.34	**<0.001**
	LDL	0.25	**<0.001**	0.40	**<0.001**
	Non-HDL	0.22	**0.001**	0.39	**<0.001**
	VLDL	-0.02	0.74	-0.03	0.56
	TG	-0.03	0.71	-0.03	0.56
	TC/HDL	-0.01	0.94	0.10	0.07
PC ae C34:2	TC	0.40	**<0.001**	0.44	**<0.001**
	HDL	0.46	**<0.001**	0.36	**<0.001**
	LDL	0.35	**<0.001**	0.43	**<0.001**
	Non-HDL	0.24	**<0.001**	0.37	**<0.001**
	VLDL	-0.13	0.06	-0.18	**<0.001**
	TG	-0.13	0.06	-0.18	**<0.001**
	TC/HDL	-0.09	0.17	0.05	0.32
PC ae C34:3	TC	0.28	**<0.001**	0.44	**<0.001**
	HDL	0.50	**<0.001**	0.37	**<0.001**
	LDL	0.23	**<0.001**	0.43	**<0.001**
	Non-HDL	0.11	0.11	0.36	**<0.001**
	VLDL	-0.27	**<0.001**	-0.20	**<0.001**
	TG	-0.27	**<0.001**	-0.20	**<0.001**
	TC/HDL	-0.22	**<0.001**	0.02	0.66
PC ae C36:0	TC	0.36	**<0.001**	0.31	**<0.001**
	HDL	0.27	**<0.001**	0.21	**<0.001**
	LDL	0.29	**<0.001**	0.29	**<0.001**
	Non-HDL	0.24	**<0.001**	0.27	**<0.001**
	VLDL	-0.02	0.75	-0.01	0.83
	TG	-0.02	0.74	-0.01	0.83
	TC/HDL	0.02	0.73	0.09	0.08
PC ae C36:1	TC	0.40	**<0.001**	0.38	**<0.001**
	HDL	0.26	**<0.001**	0.25	**<0.001**

	LDL	0.30	**<0.001**	0.32	**<0.001**
	Non-HDL	0.29	**<0.001**	0.34	**<0.001**
	VLDL	0.08	0.24	0.04	0.43
	TG	0.08	0.25	0.04	0.43
	TC/HDL	0.06	0.35	0.10	0.05
PC ae C36:2	TC	0.35	**<0.001**	0.42	**<0.001**
	HDL	0.35	**<0.001**	0.29	**<0.001**
	LDL	0.26	**<0.001**	0.40	**<0.001**
	Non-HDL	0.22	**0.001**	0.36	**<0.001**
	VLDL	-0.04	0.53	-0.10	0.06
	TG	-0.04	0.51	-0.10	0.06
	TC/HDL	-0.02	0.78	0.10	0.07
PC ae C36:3	TC	0.36	**<0.001**	0.47	**<0.001**
	HDL	0.39	**<0.001**	0.35	**<0.001**
	LDL	0.32	**<0.001**	0.46	**<0.001**
	Non-HDL	0.22	**0.001**	0.41	**<0.001**
	VLDL	-0.08	0.22	-0.15	**0.005**
	TG	-0.09	0.21	-0.15	**0.005**
	TC/HDL	-0.07	0.29	0.09	0.10
PC ae C38:0	TC	0.42	**<0.001**	0.42	**<0.001**
	HDL	0.13	0.05	0.30	**<0.001**
	LDL	0.30	**<0.001**	0.36	**<0.001**
	Non-HDL	0.37	**<0.001**	0.35	**<0.001**
	VLDL	0.27	**<0.001**	0.03	0.54
	TG	0.28	**<0.001**	0.03	0.53
	TC/HDL	0.17	**0.01**	0.07	0.16
PC ae C38:4	TC	0.26	**<0.001**	0.29	**<0.001**
	HDL	0.16	**0.02**	0.24	**<0.001**
	LDL	0.18	**0.007**	0.25	**<0.001**
	Non-HDL	0.18	**0.007**	0.25	**<0.001**
	VLDL	0.01	0.87	-0.01	0.79
	TG	0.01	0.89	-0.01	0.79
	TC/HDL	0.04	0.53	0.04	0.48
PC ae C38:5	TC	0.30	**<0.001**	0.39	**<0.001**
	HDL	0.13	0.05	0.29	**<0.001**
	LDL	0.26	**<0.001**	0.36	**<0.001**
	Non-HDL	0.24	**<0.001**	0.35	**<0.001**
	VLDL	-0.01	0.86	-0.05	0.37
	TG	-0.01	0.84	-0.05	0.37
	TC/HDL	0.11	0.10	0.08	0.14
PC ae C38:6	TC	0.41	**<0.001**	0.47	**<0.001**
	HDL	0.24	**<0.001**	0.35	**<0.001**
	LDL	0.34	**<0.001**	0.44	**<0.001**
	Non-HDL	0.32	**<0.001**	0.41	**<0.001**
	VLDL	0.02	0.81	-0.05	0.38

	TG	0.02	0.82	-0.05	0.39
	TC/HDL	0.09	0.17	0.10	0.06
PC ae C40:1	TC	0.43	**<0.001**	0.33	**<0.001**
	HDL	0.26	**<0.001**	0.26	**<0.001**
	LDL	0.33	**<0.001**	0.26	**<0.001**
	Non-HDL	0.32	**<0.001**	0.27	**<0.001**
	VLDL	0.11	0.10	0.03	0.53
	TG	0.11	0.10	0.03	0.52
	TC/HDL	0.07	0.27	0.03	0.51
PC ae C40:2	TC	0.18	**0.008**	0.36	**<0.001**
	HDL	0.27	**<0.001**	0.23	**<0.001**
	LDL	0.19	**0.005**	0.36	**<0.001**
	Non-HDL	0.09	0.20	0.32	**<0.001**
	VLDL	-0.16	**0.02**	-0.06	0.25
	TG	-0.16	**0.02**	-0.06	0.25
	TC/HDL	-0.08	0.24	0.10	**0.04**
PC ae C40:4	TC	0.24	**<0.001**	0.31	**<0.001**
	HDL	0.23	**<0.001**	0.21	**<0.001**
	LDL	0.22	**0.001**	0.30	**<0.001**
	Non-HDL	0.13	0.05	0.27	**<0.001**
	VLDL	-0.09	0.19	-0.07	0.19
	TG	-0.09	0.18	-0.07	0.19
	TC/HDL	-0.03	0.69	0.07	0.18
PC ae C40:5	TC	0.27	**<0.001**	0.37	**<0.001**
	HDL	0.13	0.05	0.27	**<0.001**
	LDL	0.23	**<0.001**	0.36	**<0.001**
	Non-HDL	0.21	**0.001**	0.33	**<0.001**
	VLDL	-0.03	0.70	-0.08	0.14
	TG	-0.03	0.69	-0.08	0.14
	TC/HDL	0.08	0.22	0.07	0.17
PC ae C40:6	TC	0.28	**<0.001**	0.37	**<0.001**
	HDL	0.26	**<0.001**	0.26	**<0.001**
	LDL	0.21	**0.001**	0.36	**<0.001**
	Non-HDL	0.19	**0.005**	0.32	**<0.001**
	VLDL	-0.11	0.12	-0.07	0.19
	TG	-0.11	0.11	-0.07	0.19
	TC/HDL	-0.01	0.90	0.09	0.10
PC ae C42:0	TC	0.12	0.09	0.03	0.57
	HDL	0.03	0.64	0.02	0.72
	LDL	0.05	0.50	0.02	0.66
	Non-HDL	0.11	0.09	0.00	0.96
	VLDL	0.18	**0.006**	-0.03	0.56
	TG	0.18	**0.006**	-0.03	0.56
	TC/HDL	0.06	0.35	-0.05	0.30
PC ae C42:1	TC	0.37	**<0.001**	0.38	**<0.001**

	HDL	0.22	**0.001**	0.26	**<0.001**
	LDL	0.26	**<0.001**	0.34	**<0.001**
	Non-HDL	0.29	**<0.001**	0.33	**<0.001**
	VLDL	0.07	0.28	0.03	0.57
	TG	0.07	0.29	0.03	0.57
	TC/HDL	0.08	0.24	0.07	0.21
PC ae C42:2	TC	0.40	**<0.001**	0.47	**<0.001**
	HDL	0.32	**<0.001**	0.27	**<0.001**
	LDL	0.34	**<0.001**	0.45	**<0.001**
	Non-HDL	0.29	**<0.001**	0.41	**<0.001**
	VLDL	-0.07	0.31	-0.05	0.32
	TG	-0.07	0.30	-0.05	0.31
	TC/HDL	0.01	0.86	0.11	**0.03**
PC ae C42:3	TC	0.33	**<0.001**	0.42	**<0.001**
	HDL	0.37	**<0.001**	0.28	**<0.001**
	LDL	0.29	**<0.001**	0.41	**<0.001**
	Non-HDL	0.19	**0.004**	0.35	**<0.001**
	VLDL	-0.17	**0.01**	-0.11	**0.04**
	TG	-0.18	**0.009**	-0.11	**0.04**
	TC/HDL	-0.07	0.29	0.09	0.10
PC ae C42:4	TC	0.13	0.06	0.35	**<0.001**
	HDL	0.28	**<0.001**	0.27	**<0.001**
	LDL	0.13	0.05	0.36	**<0.001**
	Non-HDL	0.01	0.93	0.30	**<0.001**
	VLDL	-0.27	**<0.001**	-0.15	**0.005**
	TG	-0.27	**<0.001**	-0.15	**0.005**
	TC/HDL	-0.16	**0.02**	0.06	0.28
PC ae C42:5	TC	0.14	**0.03**	0.27	**<0.001**
	HDL	0.18	**0.007**	0.22	**<0.001**
	LDL	0.14	**0.03**	0.30	**<0.001**
	Non-HDL	0.06	0.35	0.24	**<0.001**
	VLDL	-0.22	**0.001**	-0.15	**0.003**
	TG	-0.22	**<0.001**	-0.15	**0.003**
	TC/HDL	-0.05	0.44	0.05	0.37
PC ae C44:3	TC	0.18	**0.008**	0.27	**<0.001**
	HDL	0.24	**<0.001**	0.22	**<0.001**
	LDL	0.19	**0.005**	0.25	**<0.001**
	Non-HDL	0.10	0.13	0.22	**<0.001**
	VLDL	-0.13	0.06	-0.11	**0.03**
	TG	-0.13	0.06	-0.11	**0.03**
	TC/HDL	-0.04	0.58	0.04	0.46
PC ae C44:4	TC	0.14	**0.03**	0.30	**<0.001**
	HDL	0.20	**0.003**	0.27	**<0.001**
	LDL	0.13	0.05	0.31	**<0.001**
	Non-HDL	0.05	0.50	0.24	**<0.001**

	VLDL	-0.21	**0.002**	-0.14	**0.006**
	TG	-0.21	**0.002**	-0.14	**0.006**
	TC/HDL	-0.08	0.22	0.01	0.92
PC ae C44:5	TC	0.07	0.32	0.22	**<0.001**
	HDL	0.20	**0.003**	0.24	**<0.001**
	LDL	0.08	0.21	0.24	**<0.001**
	Non-HDL	-0.01	0.86	0.17	**<0.001**
	VLDL	-0.28	**<0.001**	-0.16	**0.003**
	TG	-0.28	**<0.001**	-0.16	**0.003**
	TC/HDL	-0.12	0.08	-0.02	0.75
PC ae C44:6	TC	0.09	0.17	0.21	**<0.001**
	HDL	0.24	**<0.001**	0.21	**<0.001**
	LDL	0.10	0.13	0.26	**<0.001**
	Non-HDL	0.00	0.99	0.17	**<0.001**
	VLDL	-0.30	**<0.001**	-0.22	**<0.001**
	TG	-0.30	**<0.001**	-0.22	**<0.001**
	TC/HDL	-0.14	**0.03**	-0.01	0.92

BMI: body mass index; CODING: the Complex Diseases in the Newfoundland population: Environment and Genetics; NFOAS: the Newfoundland Osteoarthritis Study; a: acyl; aa: acyl-acyl; ae: acyl-alkyl; C0: carnitine; C3: propionylcarnitine; Cx:y: where x is the number of carbons in the fatty acid side chain; y is the number of double bonds in the fatty acid side chain; PC: phophatidylcholine; TC: total cholesterol; HDL: high-density lipoprotein cholesterol; LDL: low-density lipoprotein cholesterol; VLDL: very low-density lipoprotein cholesterol; TG: triglycerides.

P values were obtained by Spearman's rank correlation.

Table S6. Association between standardized obesity-associated metabolite concentrations and blood lipids profile in the CODING and NFOAS cohorts

Metabolites	Lipid (Ratio)	CODING (n=224)		NFOAS (n=382)	
		Rho	*p* value	Rho	*p* value
Alanine	TC	0.11	0.10	-0.03	0.51
	HDL	-0.16	**0.02**	-0.19	**<0.001**
	LDL	0.10	0.14	-0.08	0.14
	Non-HDL	0.12	0.07	0.00	0.97
	VLDL	0.28	**<0.001**	0.27	**<0.001**
	TG	0.28	**<0.001**	0.27	**<0.001**
	TC/HDL	0.17	**0.01**	0.14	**0.008**
Asparagine	TC	-0.02	0.76	-0.09	0.07
	HDL	0.03	0.61	-0.10	0.05
	LDL	-0.01	0.88	-0.06	0.23
	Non-HDL	-0.07	0.32	-0.08	0.11
	VLDL	0.01	0.88	-0.07	0.18
	TG	0.01	0.88	-0.07	0.18
	TC/HDL	-0.09	0.20	0.00	1.00
Citrulline	TC	0.07	0.32	0.08	0.13
	HDL	0.14	**0.03**	0.05	0.33
	LDL	0.11	0.10	0.09	0.08
	Non-HDL	0.00	0.95	0.07	0.18
	VLDL	-0.05	0.45	-0.08	0.11
	TG	-0.05	0.45	-0.08	0.10
	TC/HDL	-0.06	0.38	0.00	0.93
Glutamate	TC	0.07	0.29	-0.08	0.14
	HDL	-0.30	**<0.001**	-0.28	**<0.001**
	LDL	0.05	0.50	-0.12	**0.02**
	Non-HDL	0.19	**0.006**	-0.02	0.65
	VLDL	0.33	**<0.001**	0.30	**<0.001**
	TG	0.33	**<0.001**	0.30	**<0.001**
	TC/HDL	0.34	**<0.001**	0.14	**0.006**
Glycine	TC	0.05	0.43	0.13	**0.01**
	HDL	0.26	**<0.001**	0.09	0.10
	LDL	0.09	0.17	0.14	**0.009**
	Non-HDL	-0.05	0.46	0.10	0.05
	VLDL	-0.16	**0.02**	-0.06	0.25
	TG	-0.16	**0.02**	-0.06	0.24
	TC/HDL	-0.18	**0.006**	0.00	0.98
Isoleucine	TC	0.01	0.91	-0.17	**<0.001**
	HDL	-0.35	**<0.001**	-0.38	**<0.001**
	LDL	0.01	0.85	-0.14	**0.007**
	Non-HDL	0.08	0.22	-0.05	0.37
	VLDL	0.31	**<0.001**	0.25	**<0.001**

	TG	0.31	**<0.001**	0.25	**<0.001**
	TC/HDL	0.26	**<0.001**	0.25	**<0.001**
Kynurenine	TC	0.04	0.56	-0.07	0.20
	HDL	-0.16	**0.02**	-0.04	0.40
	LDL	0.03	0.65	-0.08	0.13
	Non-HDL	0.11	0.11	-0.04	0.47
	VLDL	0.16	**0.02**	0.09	0.07
	TG	0.16	**0.02**	0.09	0.07
	TC/HDL	0.18	**0.007**	0.02	0.74
Leucine	TC	0.11	0.10	-0.09	0.07
	HDL	-0.22	**0.001**	-0.31	**<0.001**
	LDL	0.08	0.24	-0.07	0.19
	Non-HDL	0.17	**0.01**	0.01	0.89
	VLDL	0.24	**<0.001**	0.21	**<0.001**
	TG	0.24	**<0.001**	0.21	**<0.001**
	TC/HDL	0.26	**<0.001**	0.25	**<0.001**
Phenylalanine	TC	0.12	0.06	-0.04	0.43
	HDL	-0.17	**0.01**	-0.20	**<0.001**
	LDL	0.09	0.20	-0.02	0.73
	Non-HDL	0.16	**0.02**	0.02	0.65
	VLDL	0.21	**0.002**	0.13	**0.01**
	TG	0.21	**0.002**	0.13	**0.01**
	TC/HDL	0.22	**<0.001**	0.15	**0.003**
Serine	TC	-0.03	0.64	0.03	0.55
	HDL	0.06	0.38	-0.10	0.06
	LDL	-0.01	0.85	0.08	0.12
	Non-HDL	-0.09	0.20	0.06	0.27
	VLDL	-0.10	0.14	-0.07	0.20
	TG	-0.10	0.13	-0.07	0.20
	TC/HDL	-0.08	0.24	0.11	**0.03**
Tyrosine	TC	0.14	**0.04**	-0.04	0.38
	HDL	-0.10	0.14	-0.14	**0.006**
	LDL	0.15	**0.03**	0.01	0.92
	Non-HDL	0.15	**0.02**	0.01	0.83
	VLDL	0.21	**0.002**	0.00	0.98
	TG	0.21	**0.002**	0.00	0.97
	TC/HDL	0.19	**0.004**	0.12	**0.02**
Valine	TC	0.17	**0.01**	-0.13	**0.01**
	HDL	-0.30	**<0.001**	-0.34	**<0.001**
	LDL	0.10	0.13	-0.09	0.07
	Non-HDL	0.26	**<0.001**	-0.01	0.77
	VLDL	0.38	**<0.001**	0.23	**<0.001**
	TG	0.38	**<0.001**	0.23	**<0.001**
	TC/HDL	0.35	**<0.001**	0.26	**<0.001**
C0	TC	0.20	**0.002**	-0.06	0.23

	HDL	-0.28	**<0.001**	-0.15	**0.005**
	LDL	0.18	**0.006**	-0.06	0.21
	Non-HDL	0.29	**<0.001**	-0.03	0.57
	VLDL	0.30	**<0.001**	0.08	0.10
	TG	0.30	**<0.001**	0.08	0.10
	TC/HDL	0.37	**<0.001**	0.05	0.30
C3	TC	0.12	0.09	-0.10	0.05
	HDL	-0.39	**<0.001**	-0.21	**<0.001**
	LDL	0.13	0.05	-0.11	**0.04**
	Non-HDL	0.23	**<0.001**	-0.05	0.35
	VLDL	0.39	**<0.001**	0.14	**0.005**
	TG	0.39	**<0.001**	0.15	**0.005**
	TC/HDL	0.40	**<0.001**	0.08	0.14
LysoPC a C16:0	TC	0.25	**<0.001**	0.25	**<0.001**
	HDL	-0.07	0.31	0.07	0.18
	LDL	0.19	**0.005**	0.21	**<0.001**
	Non-HDL	0.26	**<0.001**	0.25	**<0.001**
	VLDL	0.29	**<0.001**	0.16	**0.002**
	TG	0.29	**<0.001**	0.16	**0.002**
	TC/HDL	0.25	**<0.001**	0.15	**0.005**
LysoPC a C17:0	TC	0.17	**0.01**	0.16	**0.002**
	HDL	0.07	0.28	0.05	0.39
	LDL	0.16	**0.02**	0.13	**0.009**
	Non-HDL	0.14	**0.03**	0.15	**0.004**
	VLDL	0.03	0.68	0.05	0.35
	TG	0.03	0.69	0.05	0.36
	TC/HDL	0.07	0.33	0.07	0.19
LysoPC a C18:0	TC	0.29	**<0.001**	0.27	**<0.001**
	HDL	0.01	0.84	0.10	0.06
	LDL	0.26	**<0.001**	0.25	**<0.001**
	Non-HDL	0.28	**<0.001**	0.27	**<0.001**
	VLDL	0.17	**0.01**	0.11	**0.04**
	TG	0.16	**0.02**	0.11	**0.04**
	TC/HDL	0.21	**0.001**	0.13	**0.01**
LysoPC a C18:1	TC	0.17	**0.01**	0.21	**<0.001**
	HDL	0.06	0.39	0.16	**0.002**
	LDL	0.17	**0.01**	0.17	**<0.001**
	Non-HDL	0.14	**0.04**	0.18	**<0.001**
	VLDL	0.06	0.40	0.02	0.68
	TG	0.06	0.41	0.02	0.68
	TC/HDL	0.06	0.35	0.02	0.72
LysoPC a C18:2	TC	0.16	**0.02**	0.25	**<0.001**
	HDL	0.14	**0.04**	0.19	**<0.001**
	LDL	0.18	**0.008**	0.24	**<0.001**
	Non-HDL	0.10	0.16	0.21	**<0.001**

	VLDL	-0.04	0.51	-0.09	0.07
	TG	-0.05	0.50	-0.09	0.07
	TC/HDL	-0.02	0.82	0.03	0.56
LysoPC a C20:4	TC	0.07	0.33	0.02	0.66
	HDL	-0.07	0.33	0.06	0.29
	LDL	0.05	0.42	-0.04	0.43
	Non-HDL	0.08	0.23	0.00	0.93
	VLDL	0.14	**0.04**	0.08	0.11
	TG	0.14	**0.04**	0.08	0.11
	TC/HDL	0.11	0.12	-0.05	0.32
PC aa C32:0	TC	0.41	**<0.001**	0.48	**<0.001**
	HDL	0.15	**0.02**	0.26	**<0.001**
	LDL	0.30	**<0.001**	0.43	**<0.001**
	Non-HDL	0.32	**<0.001**	0.44	**<0.001**
	VLDL	0.11	0.09	0.10	0.06
	TG	0.11	0.10	0.10	0.06
	TC/HDL	0.13	0.05	0.19	**<0.001**
PC aa C32:1	TC	0.29	**<0.001**	0.35	**<0.001**
	HDL	-0.02	0.74	0.30	**<0.001**
	LDL	0.17	**0.01**	0.27	**<0.001**
	Non-HDL	0.25	**<0.001**	0.32	**<0.001**
	VLDL	0.37	**<0.001**	0.10	**0.04**
	TG	0.37	**<0.001**	0.10	**0.04**
	TC/HDL	0.18	**0.009**	0.06	0.22
PC aa C38:0	TC	0.34	**<0.001**	0.36	**<0.001**
	HDL	0.25	**<0.001**	0.26	**<0.001**
	LDL	0.29	**<0.001**	0.34	**<0.001**
	Non-HDL	0.25	**<0.001**	0.31	**<0.001**
	VLDL	-0.02	0.82	-0.07	0.21
	TG	-0.02	0.81	-0.07	0.21
	TC/HDL	0.05	0.48	0.07	0.17
PC aa C38:3	TC	0.39	**<0.001**	0.50	**<0.001**
	HDL	-0.08	0.22	0.24	**<0.001**
	LDL	0.28	**<0.001**	0.43	**<0.001**
	Non-HDL	0.40	**<0.001**	0.49	**<0.001**
	VLDL	0.43	**<0.001**	0.22	**<0.001**
	TG	0.43	**<0.001**	0.22	**<0.001**
	TC/HDL	0.32	**<0.001**	0.24	**<0.001**
PC aa C38:4	TC	0.31	**<0.001**	0.21	**<0.001**
	HDL	-0.08	0.25	0.15	**0.005**
	LDL	0.19	**0.005**	0.12	**0.02**
	Non-HDL	0.31	**<0.001**	0.19	**<0.001**
	VLDL	0.42	**<0.001**	0.19	**<0.001**
	TG	0.42	**<0.001**	0.19	**<0.001**
	TC/HDL	0.26	**<0.001**	0.05	0.32

PC aa C38:6	TC	0.33	**<0.001**	0.35	**<0.001**
	HDL	0.08	0.26	0.23	**<0.001**
	LDL	0.21	**0.002**	0.28	**<0.001**
	Non-HDL	0.31	**<0.001**	0.30	**<0.001**
	VLDL	0.29	**<0.001**	0.12	**0.02**
	TG	0.29	**<0.001**	0.12	**0.02**
	TC/HDL	0.18	**0.008**	0.08	0.12
PC aa C40:1	TC	0.16	**0.02**	0.27	**<0.001**
	HDL	0.25	**<0.001**	0.17	**<0.001**
	LDL	0.16	**0.02**	0.27	**<0.001**
	Non-HDL	0.07	0.28	0.24	**<0.001**
	VLDL	-0.15	**0.03**	-0.06	0.25
	TG	-0.15	**0.03**	-0.06	0.25
	TC/HDL	-0.07	0.31	0.09	0.08
PC aa C40:2	TC	0.12	0.07	0.23	**<0.001**
	HDL	0.25	**<0.001**	0.15	**0.004**
	LDL	0.13	0.05	0.22	**<0.001**
	Non-HDL	0.03	0.65	0.21	**<0.001**
	VLDL	-0.17	**0.01**	-0.02	0.66
	TG	-0.17	**0.01**	-0.02	0.66
	TC/HDL	-0.11	0.09	0.09	0.07
PC aa C40:3	TC	0.16	**0.02**	0.33	**<0.001**
	HDL	0.19	**0.004**	0.20	**<0.001**
	LDL	0.17	**0.01**	0.32	**<0.001**
	Non-HDL	0.09	0.20	0.30	**<0.001**
	VLDL	-0.09	0.19	-0.03	0.60
	TG	-0.09	0.19	-0.03	0.61
	TC/HDL	-0.04	0.54	0.11	**0.03**
PC aa C42:0	TC	0.17	**0.01**	0.26	**<0.001**
	HDL	0.24	**<0.001**	0.22	**<0.001**
	LDL	0.14	**0.03**	0.31	**<0.001**
	Non-HDL	0.07	0.29	0.23	**<0.001**
	VLDL	-0.18	**0.007**	-0.19	**<0.001**
	TG	-0.18	**0.006**	-0.19	**<0.001**
	TC/HDL	-0.09	0.20	0.03	0.56
PC aa C42:1	TC	0.20	**0.003**	0.27	**<0.001**
	HDL	0.32	**<0.001**	0.23	**<0.001**
	LDL	0.17	**0.01**	0.31	**<0.001**
	Non-HDL	0.07	0.31	0.23	**<0.001**
	VLDL	-0.21	**0.002**	-0.16	**0.002**
	TG	-0.21	**0.002**	-0.16	**0.002**
	TC/HDL	-0.14	**0.04**	0.05	0.30
PC aa C42:2	TC	0.24	**<0.001**	0.26	**<0.001**
	HDL	0.26	**<0.001**	0.19	**<0.001**
	LDL	0.18	**0.009**	0.28	**<0.001**

	Non-HDL	0.13	0.06	0.22	**<0.001**
	VLDL	-0.04	0.52	-0.17	**0.001**
	TG	-0.04	0.51	-0.17	**0.001**
	TC/HDL	-0.06	0.39	0.03	0.52
PC aa C42:4	TC	0.22	**0.001**	0.26	**<0.001**
	HDL	0.22	**0.001**	0.16	**0.001**
	LDL	0.18	**0.008**	0.25	**<0.001**
	Non-HDL	0.14	**0.04**	0.23	**<0.001**
	VLDL	-0.03	0.68	-0.02	0.68
	TG	-0.03	0.67	-0.02	0.68
	TC/HDL	-0.03	0.67	0.06	0.23
PC aa C42:5	TC	0.27	**<0.001**	0.31	**<0.001**
	HDL	0.07	0.27	0.16	**0.002**
	LDL	0.20	**0.003**	0.28	**<0.001**
	Non-HDL	0.23	**<0.001**	0.29	**<0.001**
	VLDL	0.12	0.09	0.08	0.14
	TG	0.12	0.09	0.08	0.13
	TC/HDL	0.09	0.19	0.12	**0.02**
PC aa C42:6	TC	0.22	**0.001**	0.24	**<0.001**
	HDL	0.22	**0.001**	0.19	**<0.001**
	LDL	0.16	**0.02**	0.19	**<0.001**
	Non-HDL	0.14	**0.04**	0.21	**<0.001**
	VLDL	0.07	0.28	0.05	0.35
	TG	0.07	0.29	0.05	0.34
	TC/HDL	-0.02	0.75	0.02	0.74
PC ae C30:0	TC	0.24	**<0.001**	0.38	**<0.001**
	HDL	0.30	**<0.001**	0.26	**<0.001**
	LDL	0.20	**0.003**	0.38	**<0.001**
	Non-HDL	0.13	0.05	0.33	**<0.001**
	VLDL	-0.10	0.13	-0.12	**0.02**
	TG	-0.11	0.12	-0.12	**0.02**
	TC/HDL	-0.09	0.21	0.08	0.10
PC ae C32:1	TC	0.16	**0.02**	0.43	**<0.001**
	HDL	0.32	**<0.001**	0.38	**<0.001**
	LDL	0.14	**0.04**	0.43	**<0.001**
	Non-HDL	0.03	0.66	0.37	**<0.001**
	VLDL	-0.26	**<0.001**	-0.16	**0.001**
	TG	-0.26	**<0.001**	-0.16	**0.001**
	TC/HDL	-0.15	**0.02**	0.07	0.19
PC ae C32:2	TC	0.16	**0.02**	0.43	**<0.001**
	HDL	0.32	**<0.001**	0.41	**<0.001**
	LDL	0.17	**0.01**	0.40	**<0.001**
	Non-HDL	0.03	0.66	0.35	**<0.001**
	VLDL	-0.21	**0.002**	-0.15	**0.003**
	TG	-0.21	**0.002**	-0.15	**0.003**

	TC/HDL	-0.16	**0.02**	0.02	0.74
PC ae C34:0	TC	0.37	**<0.001**	0.39	**<0.001**
	HDL	0.18	**0.007**	0.23	**<0.001**
	LDL	0.27	**<0.001**	0.37	**<0.001**
	Non-HDL	0.30	**<0.001**	0.36	**<0.001**
	VLDL	0.11	0.11	-0.02	0.73
	TG	0.11	0.12	-0.02	0.73
	TC/HDL	0.11	0.09	0.14	**0.006**
PC ae C34:1	TC	0.33	**<0.001**	0.45	**<0.001**
	HDL	0.27	**<0.001**	0.34	**<0.001**
	LDL	0.25	**<0.001**	0.40	**<0.001**
	Non-HDL	0.22	**0.001**	0.39	**<0.001**
	VLDL	-0.02	0.74	-0.03	0.56
	TG	-0.03	0.71	-0.03	0.56
	TC/HDL	-0.01	0.94	0.10	0.07
PC ae C34:2	TC	0.40	**<0.001**	0.44	**<0.001**
	HDL	0.46	**<0.001**	0.36	**<0.001**
	LDL	0.35	**<0.001**	0.43	**<0.001**
	Non-HDL	0.24	**<0.001**	0.37	**<0.001**
	VLDL	-0.13	0.06	-0.18	**<0.001**
	TG	-0.13	0.06	-0.18	**<0.001**
	TC/HDL	-0.09	0.17	0.05	0.32
PC ae C34:3	TC	0.28	**<0.001**	0.44	**<0.001**
	HDL	0.50	**<0.001**	0.37	**<0.001**
	LDL	0.23	**<0.001**	0.43	**<0.001**
	Non-HDL	0.11	0.11	0.36	**<0.001**
	VLDL	-0.27	**<0.001**	-0.20	**<0.001**
	TG	-0.27	**<0.001**	-0.20	**<0.001**
	TC/HDL	-0.22	**<0.001**	0.02	0.66
PC ae C36:0	TC	0.36	**<0.001**	0.31	**<0.001**
	HDL	0.27	**<0.001**	0.21	**<0.001**
	LDL	0.29	**<0.001**	0.29	**<0.001**
	Non-HDL	0.24	**<0.001**	0.27	**<0.001**
	VLDL	-0.02	0.75	-0.01	0.83
	TG	-0.02	0.74	-0.01	0.83
	TC/HDL	0.02	0.73	0.09	0.08
PC ae C36:1	TC	0.40	**<0.001**	0.38	**<0.001**
	HDL	0.26	**<0.001**	0.25	**<0.001**
	LDL	0.30	**<0.001**	0.32	**<0.001**
	Non-HDL	0.29	**<0.001**	0.34	**<0.001**
	VLDL	0.08	0.24	0.04	0.43
	TG	0.08	0.25	0.04	0.43
	TC/HDL	0.06	0.35	0.10	0.05
PC ae C36:2	TC	0.35	**<0.001**	0.42	**<0.001**
	HDL	0.35	**<0.001**	0.29	**<0.001**

	LDL	0.26	**<0.001**	0.40	**<0.001**
	Non-HDL	0.22	**0.001**	0.36	**<0.001**
	VLDL	-0.04	0.53	-0.10	0.06
	TG	-0.04	0.51	-0.10	0.06
	TC/HDL	-0.02	0.78	0.10	0.07
PC ae C36:3	TC	0.36	**<0.001**	0.47	**<0.001**
	HDL	0.39	**<0.001**	0.35	**<0.001**
	LDL	0.32	**<0.001**	0.46	**<0.001**
	Non-HDL	0.22	**0.001**	0.41	**<0.001**
	VLDL	-0.08	0.22	-0.15	**0.005**
	TG	-0.09	0.21	-0.15	**0.005**
	TC/HDL	-0.07	0.29	0.09	0.10
PC ae C38:0	TC	0.42	**<0.001**	0.42	**<0.001**
	HDL	0.13	0.05	0.30	**<0.001**
	LDL	0.30	**<0.001**	0.36	**<0.001**
	Non-HDL	0.37	**<0.001**	0.35	**<0.001**
	VLDL	0.27	**<0.001**	0.03	0.54
	TG	0.28	**<0.001**	0.03	0.53
	TC/HDL	0.17	**0.01**	0.07	0.16
PC ae C40:1	TC	0.43	**<0.001**	0.33	**<0.001**
	HDL	0.26	**<0.001**	0.26	**<0.001**
	LDL	0.33	**<0.001**	0.26	**<0.001**
	Non-HDL	0.32	**<0.001**	0.27	**<0.001**
	VLDL	0.11	0.10	0.03	0.53
	TG	0.11	0.10	0.03	0.52
	TC/HDL	0.07	0.27	0.03	0.51
PC ae C40:2	TC	0.18	**0.008**	0.36	**<0.001**
	HDL	0.27	**<0.001**	0.23	**<0.001**
	LDL	0.19	**0.005**	0.36	**<0.001**
	Non-HDL	0.09	0.20	0.32	**<0.001**
	VLDL	-0.16	**0.02**	-0.06	0.25
	TG	-0.16	**0.02**	-0.06	0.25
	TC/HDL	-0.08	0.24	0.10	**0.04**
PC ae C40:3	TC	0.21	**0.002**	0.32	**<0.001**
	HDL	0.35	**<0.001**	0.21	**<0.001**
	LDL	0.19	**0.004**	0.31	**<0.001**
	Non-HDL	0.09	0.20	0.28	**<0.001**
	VLDL	-0.22	**<0.001**	-0.06	0.26
	TG	-0.22	**<0.001**	-0.06	0.26
	TC/HDL	-0.13	0.05	0.09	0.10
PC ae C40:4	TC	0.24	**<0.001**	0.31	**<0.001**
	HDL	0.23	**<0.001**	0.21	**<0.001**
	LDL	0.22	**0.001**	0.30	**<0.001**
	Non-HDL	0.13	0.05	0.27	**<0.001**
	VLDL	-0.09	0.19	-0.07	0.19

	TG	-0.09	0.18	-0.07	0.19
	TC/HDL	-0.03	0.69	0.07	0.18
PC ae C40:5	TC	0.27	**<0.001**	0.37	**<0.001**
	HDL	0.13	0.05	0.27	**<0.001**
	LDL	0.23	**<0.001**	0.36	**<0.001**
	Non-HDL	0.21	**0.001**	0.33	**<0.001**
	VLDL	-0.03	0.70	-0.08	0.14
	TG	-0.03	0.69	-0.08	0.14
	TC/HDL	0.08	0.22	0.07	0.17
PC ae C40:6	TC	0.28	**<0.001**	0.37	**<0.001**
	HDL	0.26	**<0.001**	0.26	**<0.001**
	LDL	0.21	**0.001**	0.36	**<0.001**
	Non-HDL	0.19	**0.005**	0.32	**<0.001**
	VLDL	-0.11	0.12	-0.07	0.19
	TG	-0.11	0.11	-0.07	0.19
	TC/HDL	-0.01	0.90	0.09	0.10
PC ae C42:0	TC	0.12	0.09	0.03	0.57
	HDL	0.03	0.64	0.02	0.72
	LDL	0.05	0.50	0.02	0.66
	Non-HDL	0.11	0.09	0.00	0.96
	VLDL	0.18	**0.006**	-0.03	0.56
	TG	0.18	**0.006**	-0.03	0.56
	TC/HDL	0.06	0.35	-0.05	0.30
PC ae C42:1	TC	0.37	**<0.001**	0.38	**<0.001**
	HDL	0.22	**0.001**	0.26	**<0.001**
	LDL	0.26	**<0.001**	0.34	**<0.001**
	Non-HDL	0.29	**<0.001**	0.33	**<0.001**
	VLDL	0.07	0.28	0.03	0.57
	TG	0.07	0.29	0.03	0.57
	TC/HDL	0.08	0.24	0.07	0.21
PC ae C42:2	TC	0.40	**<0.001**	0.47	**<0.001**
	HDL	0.32	**<0.001**	0.27	**<0.001**
	LDL	0.34	**<0.001**	0.45	**<0.001**
	Non-HDL	0.29	**<0.001**	0.41	**<0.001**
	VLDL	-0.07	0.31	-0.05	0.32
	TG	-0.07	0.30	-0.05	0.31
	TC/HDL	0.01	0.86	0.11	**0.03**
PC ae C42:3	TC	0.33	**<0.001**	0.42	**<0.001**
	HDL	0.37	**<0.001**	0.28	**<0.001**
	LDL	0.29	**<0.001**	0.41	**<0.001**
	Non-HDL	0.19	**0.004**	0.35	**<0.001**
	VLDL	-0.17	**0.01**	-0.11	**0.04**
	TG	-0.18	**0.009**	-0.11	**0.04**
	TC/HDL	-0.07	0.29	0.09	0.10
PC ae C42:4	TC	0.13	0.06	0.35	**<0.001**

	HDL	0.28	**<0.001**	0.27	**<0.001**
	LDL	0.13	0.05	0.36	**<0.001**
	Non-HDL	0.01	0.93	0.30	**<0.001**
	VLDL	-0.27	**<0.001**	-0.15	**0.005**
	TG	-0.27	**<0.001**	-0.15	**0.005**
	TC/HDL	-0.16	**0.02**	0.06	0.28
PC ae C42:5	TC	0.14	**0.03**	0.27	**<0.001**
	HDL	0.18	**0.007**	0.22	**<0.001**
	LDL	0.14	**0.03**	0.30	**<0.001**
	Non-HDL	0.06	0.35	0.24	**<0.001**
	VLDL	-0.22	**0.001**	-0.15	**0.003**
	TG	-0.22	**<0.001**	-0.15	**0.003**
	TC/HDL	-0.05	0.44	0.05	0.37
PC ae C44:3	TC	0.18	**0.008**	0.27	**<0.001**
	HDL	0.24	**<0.001**	0.22	**<0.001**
	LDL	0.19	**0.005**	0.25	**<0.001**
	Non-HDL	0.10	0.13	0.22	**<0.001**
	VLDL	-0.13	0.06	-0.11	**0.03**
	TG	-0.13	0.06	-0.11	**0.03**
	TC/HDL	-0.04	0.58	0.04	0.46
PC ae C44:4	TC	0.14	**0.03**	0.30	**<0.001**
	HDL	0.20	**0.003**	0.27	**<0.001**
	LDL	0.13	0.05	0.31	**<0.001**
	Non-HDL	0.05	0.50	0.24	**<0.001**
	VLDL	-0.21	**0.002**	-0.14	**0.006**
	TG	-0.21	**0.002**	-0.14	**0.006**
	TC/HDL	-0.08	0.22	0.01	0.92
PC ae C44:5	TC	0.07	0.32	0.22	**<0.001**
	HDL	0.20	**0.003**	0.24	**<0.001**
	LDL	0.08	0.21	0.24	**<0.001**
	Non-HDL	-0.01	0.86	0.17	**<0.001**
	VLDL	-0.28	**<0.001**	-0.16	**0.003**
	TG	-0.28	**<0.001**	-0.16	**0.003**
	TC/HDL	-0.12	0.08	-0.02	0.75
PC ae C44:6	TC	0.09	0.17	0.21	**<0.001**
	HDL	0.24	**<0.001**	0.21	**<0.001**
	LDL	0.10	0.13	0.26	**<0.001**
	Non-HDL	0.00	0.99	0.17	**<0.001**
	VLDL	-0.30	**<0.001**	-0.22	**<0.001**
	TG	-0.30	**<0.001**	-0.22	**<0.001**
	TC/HDL	-0.14	**0.03**	-0.01	0.92

CODING: the Complex Diseases in the Newfoundland population: Environment and Genetics; NFOAS: the Newfoundland Osteoarthritis Study; a: acyl; aa: acyl-acyl; ae: acyl-alkyl; C0: carnitine; C3: propionylcarnitine; Cx:y: where x is the number of carbons in the fatty acid side chain; y is the number of double bonds in the fatty acid side chain; PC: phophatidylcholine; TC:

total cholesterol; HDL: high-density lipoprotein cholesterol; LDL: low-density lipoprotein cholesterol; VLDL: very low-density lipoprotein cholesterol; TG: triglycerides.

P values were obtained by Spearman's rank correlation.

Table S7. Association between standardized BMI-associated metabolite concentrations and visceral fat mass in the CODING cohort

Metabolite	Visceral fat mass (n=226)	
	Rho	*p* value
Alanine	0.23	**<0.001**
Asparagine	-0.13	0.05
Glutamate	0.43	**<0.001**
Glutamine	0.04	0.52
Glycine	-0.29	**<0.001**
Isoleucine	0.43	**<0.001**
Kynurenine	0.17	**0.01**
Phenylalanine	0.12	0.08
Serine	-0.18	**0.007**
Tyrosine	0.18	**0.007**
Valine	0.36	**<0.001**
C0	0.28	**<0.001**
C3	0.49	**<0.001**
LysoPC a C16:0	0.11	0.10
LysoPC a C17:0	-0.09	0.20
LysoPC a C18:0	0.04	0.56
LysoPC a C18:1	-0.03	0.64
LysoPC a C18:2	-0.13	0.05
LysoPC a C20:3	0.16	**0.02**
LysoPC a C20:4	0.13	0.06
PC aa C30:0	-0.12	0.08
PC aa C32:0	-0.15	**0.03**
PC aa C36:6	-0.03	0.67
PC aa C38:0	-0.16	**0.02**
PC aa C38:3	0.14	**0.04**
PC aa C38:5	0.07	0.34
PC aa C38:6	-0.01	0.88
PC aa C40:1	-0.38	**<0.001**
PC aa C40:2	-0.40	**<0.001**
PC aa C40:3	-0.29	**<0.001**
PC aa C42:0	-0.28	**<0.001**
PC aa C42:1	-0.38	**<0.001**
PC aa C42:2	-0.23	**<0.001**
PC aa C42:4	-0.31	**<0.001**
PC aa C42:5	-0.11	0.11
PC aa C42:6	-0.21	**0.002**
PC ae C30:0	-0.30	**<0.001**
PC ae C32:1	-0.33	**<0.001**
PC ae C32:2	-0.37	**<0.001**
PC ae C34:0	-0.11	0.11

PC ae C34:1	-0.30	**<0.001**
PC ae C34:2	-0.42	**<0.001**
PC ae C34:3	-0.45	**<0.001**
PC ae C36:0	-0.16	**0.02**
PC ae C36:1	-0.20	**0.003**
PC ae C36:2	-0.35	**<0.001**
PC ae C36:3	-0.37	**<0.001**
PC ae C38:0	-0.01	0.91
PC ae C38:4	-0.16	**0.02**
PC ae C38:5	-0.09	0.17
PC ae C38:6	-0.10	0.14
PC ae C40:1	-0.20	**0.003**
PC ae C40:2	-0.29	**<0.001**
PC ae C40:4	-0.31	**<0.001**
PC ae C40:5	-0.14	**0.04**
PC ae C40:6	-0.26	**<0.001**
PC ae C42:0	-0.01	0.87
PC ae C42:1	-0.24	**<0.001**
PC ae C42:2	-0.33	**<0.001**
PC ae C42:3	-0.39	**<0.001**
PC ae C42:4	-0.38	**<0.001**
PC ae C42:5	-0.24	**<0.001**
PC ae C44:3	-0.38	**<0.001**
PC ae C44:4	-0.29	**<0.001**
PC ae C44:5	-0.23	**<0.001**
PC ae C44:6	-0.29	**<0.001**

BMI: body mass index; CODING: the Complex Diseases in the Newfoundland population: Environment and Genetics; a: acyl; aa: acyl-acyl; ae: acyl-alkyl; C0: carnitine; C3: propionylcarnitine; Cx:y: where x is the number of carbons in the fatty acid side chain; y is the number of double bonds in the fatty acid side chain; PC: phophatidylcholine.

P values were obtained by Spearman's rank correlation.

Table S8. Association between standardized obesity-associated metabolite concentrations and visceral fat mass in the CODING cohort

Metabolite	Visceral fat mass (n=226)	
	**Rho**	***p* value**
Alanine	0.23	**<0.001**
Asparagine	-0.13	0.05
Citrulline	-0.02	0.80
Glutamate	0.43	**<0.001**
Glycine	-0.29	**<0.001**
Isoleucine	0.43	**<0.001**
Kynurenine	0.17	**0.01**
Leucine	0.18	**0.009**
Phenylalanine	0.12	0.08
Serine	-0.18	**0.007**
Tyrosine	0.18	**0.007**
Valine	0.36	**<0.001**
C0	0.28	**<0.001**
C3	0.49	**<0.001**
LysoPC a C16:0	0.11	0.10
LysoPC a C17:0	-0.09	0.20
LysoPC a C18:0	0.04	0.56
LysoPC a C18:1	-0.03	0.64
LysoPC a C18:2	-0.13	0.05
LysoPC a C20:4	0.13	0.06
PC aa C32:0	-0.15	**0.03**
PC aa C32:1	0.10	0.15
PC aa C38:0	-0.16	**0.02**
PC aa C38:3	0.14	**0.04**
PC aa C38:4	0.19	**0.005**
PC aa C38:6	-0.01	0.88
PC aa C40:1	-0.38	**<0.001**
PC aa C40:2	-0.40	**<0.001**
PC aa C40:3	-0.29	**<0.001**
PC aa C42:0	-0.28	**<0.001**
PC aa C42:1	-0.38	**<0.001**
PC aa C42:2	-0.23	**<0.001**
PC aa C42:4	-0.31	**<0.001**
PC aa C42:5	-0.11	0.11
PC aa C42:6	-0.21	**0.002**
PC ae C30:0	-0.30	**<0.001**
PC ae C32:1	-0.33	**<0.001**
PC ae C32:2	-0.37	**<0.001**
PC ae C34:0	-0.11	0.11
PC ae C34:1	-0.30	**<0.001**

PC ae C34:2	-0.42	**<0.001**
PC ae C34:3	-0.45	**<0.001**
PC ae C36:0	-0.16	**0.02**
PC ae C36:1	-0.20	**0.003**
PC ae C36:2	-0.35	**<0.001**
PC ae C36:3	-0.37	**<0.001**
PC ae C38:0	-0.01	0.91
PC ae C40:1	-0.20	**0.003**
PC ae C40:2	-0.29	**<0.001**
PC ae C40:3	-0.44	**<0.001**
PC ae C40:4	-0.31	**<0.001**
PC ae C40:5	-0.14	**0.04**
PC ae C40:6	-0.26	**<0.001**
PC ae C42:0	-0.01	0.87
PC ae C42:1	-0.24	**<0.001**
PC ae C42:2	-0.33	**<0.001**
PC ae C42:3	-0.39	**<0.001**
PC ae C42:4	-0.38	**<0.001**
PC ae C42:5	-0.24	**<0.001**
PC ae C44:3	-0.38	**<0.001**
PC ae C44:4	-0.29	**<0.001**
PC ae C44:5	-0.23	**<0.001**
PC ae C44:6	-0.29	**<0.001**

CODING: the Complex Diseases in the Newfoundland population: Environment and Genetics; a: acyl; aa: acyl-acyl; ae: acyl-alkyl; C0: carnitine; C3: propionylcarnitine; Cx:y: where x is the number of carbons in the fatty acid side chain; y is the number of double bonds in the fatty acid side chain; PC: phophatidylcholine.

P values were obtained by Spearman's rank correlation.

Table S9. Biochemical characteristics of participants in the CODING and NFOAS cohorts

	CODING			NFOAS		
	**Obese (n=101)**	**Non-obese (n=59)**	***p* value**	**Obese (n=432)**	**Non-obese (n=56)**	***p* value**
TC (mmol/L)	5.18±1.18	5.09±1.07	0.74	4.61±1.05	5.03±0.93	**0.03**
HDL (mmol/L)	1.23±0.26	1.59±0.34	**<0.001**	1.19±0.29	1.44±0.30	**<0.001**
LDL (mmol/L)	3.18±1.03	3.04±0.91	0.36	2.69±0.89	3.03±0.80	0.05
VLDL (mmol/L)	0.77±0.51	0.44±0.25	**<0.001**	0.74±0.32	0.57±0.27	**0.003**
TG (mmol/L)	1.70±1.11	0.96±0.56	**<0.001**	1.63±0.70	1.24±0.60	**0.003**
TC/HDL	4.36±1.24	3.30±0.84	**<0.001**	4.02±1.00	3.59±0.87	**0.02**
Glucose (mmol/L)	5.96±2.01	5.19±0.83	**<0.001**	-	-	
Insulin (pmol/L)	126.00±139.00	60.50±41.50	**<0.001**	-	-	
HOMA-IR	4.95±5.48	2.28±2.10	**<0.001**	-	-	
Visceral fat mass (g)	1664.22±875.33	365.30±368.88	**<0.001**			

CODING: the Complex Diseases in the Newfoundland population: Environment and Genetics; NFOAS: the Newfoundland Osteoarthritis Study; TC: total cholesterol; HDL: high-density lipoprotein cholesterol; LDL: low-density lipoprotein cholesterol; VLDL: very low-density lipoprotein cholesterol; TG: triglycerides; HOMA-IR: homeostatic model assessment for insulin resistance.

In CODING cohort, for insulin and HOMA-IR levels, n_{obese}=90, $n_{Non\text{-}obese}$=24; in NFOAS cohort, for blood lipid profiles, n_{obese}=264, $n_{Non\text{-}obese}$=26.

P values were obtained by the Wilcoxon test or Chi-square test wherever appropriate.

Table S10. List of metabolites determined using the Biocrates AbsoluteIDQ® p180 kit

Metabolite class	Number	Metabolite name or abbreviation
Amino acids	21	Alanine, arginine, asparagine, aspartate, citrulline, glutamine, glutamate, glycine, histidine, isoleucine, leucine, lysine, methionine, ornithine, phenylalanine, proline, serine, threonine, tryptophan, tyrosine, valine
Carnitine	1	C0
Acylcarnitine	25	C2, C3, C3:1, C4, C4:1, C5, C5:1, C6(or C4:1-DC), C6:1, C8, C9, C10, C10:1, C10:2, C12, C12:1, C14, C14:1, C14:2, C16, C16:1, C16:2, C18, C18:1, C18:2
Hydroxy- and dicarboxyacylcarnitines	14	C3-OH, C4-OH(or C3-DC), C5:1-DC, C5-DC(or C6-OH), C5-M-DC, C5-OH(or C3-DC-M), C7-DC, C12-DC, C14:1-OH, C14:2-OH, C16:1-OH, C16:2-OH, C16-OH, C18:1-OH
Biogenic amines	19	acetylornithine, asymmetric dimethylarginine, total dimethylarginine, alpha-Aminoadipic acid, carnosine, creatinine, histamine, kynurenine, methioninesulfoxide, nitrotyrosine,hydroxyproline, phenylethylamine, putrescine, sarcosine, serotonin, spermidine, spermine, taurine
Lyso-phosphatidylcholines	14	lysoPC a C14:0/C16:0/C16:1/C17:0/C18:0/C18:1/C18:2/C20:3/C20:4/C26:0/C26:1/C28:0/C28:1
Diacyl-phosphatidylcholines	38	PC aa C24:0/C26:0/C28:1/C30:0/C30:2/C32:0/C32:1/C32:2/C32:3/C34:1/C34:2/C34:3/C34:4/C36:0/C36:1/C36:2/C36:3/C36:4/C36:5/C36:6/C38:0/C38:1/C38:3/C38:4/C38:5/C38:6/C40:1/C40:2/C40:3/C40:4/C40:5/C40:6/C42:0/C42:1/C42:2/C42:4/C42:5/C42:6
Acyl-alkyl-phosphatidylcholines	38	PC ae C30:0/C30:2/C32:1/C32:2/C34:0/C34:1/C34:2/C34:3/C36:0/C36:1/C36:2/C36:3/C36:4/C36:5/C38:0/C38:1/C38:2/C38:3/C38:4/C38:5/C38:6/C40:1/C40:2/C40:3/C40:4/C40:5/C40:6/C42:0/C42:1/C42:2/C42:3/C42:4/C42:5/C44:3/C44:4/C44:5/C44:6
Sphingomyelines	10	SM C16:0, SM C16:1, SM C18:0, SM C18:1, SM C20:2, SM C22:3, SM C24:0, SM 24:1, SM C26:0, SM C26:1
Hydroxysphingomyelines	5	SM (OH) C14:1, SM (OH) C16:1, SM (OH) C22:1, SM (OH) C22:2, SM (OH) C24:1
Hexose	1	H1
Total	186	

a: acyl; aa: acyl-acyl; ae: acyl-alkyl; Cx:y: where x is the number of carbons in the fatty acid side chain; y is the number of double bonds in the fatty acid side chain; DC: decarboxyl; M: methyl; OH: hydroxyl; PC: phophatidylcholine; SM: sphingomyelin.

Table S11. List of metabolite concentrations determined using the TMIC Prime Metabolomics Profiling Assay

Metabolite class	Number	Metabolite name or abbreviation
Carnitine	1	C0
Acylcarnitines	25	C2, C3, C3:1, C4, C4:1, C5, C5:1, C6(or C4:1-DC), C6:1, C8, C9, C10, C10:1, C10:2, C12, C12:1, C14, C14:1, C14:2, C16, C16:1, C16:2, C18, C18:1, C18:2
Hydroxy- and dicarboxyacylcarnitines	14	C3-OH, C4-OH(or C3-DC), C5:1-DC, C5-DC(or C6-OH), C5-M-DC, C5-OH(or C3-DC-M), C7-DC, C12-DC, C14:1-OH, C14:2-OH, C16:1-OH, C16:2-OH, C16-OH, C18:1-OH
Amino acids and derivatives	25	Alanine, arginine, asparagine, aspartate, citrulline, glutamine, glutamate, glycine, histidine, isoleucine, leucine, lysine, methionine, ornithine, phenylalanine, proline, serine, threonine, tryptophan, tyrosine, valine, betaine, creatine, phosphocreatine, methylhistidine
Amine oxides	1	Trimethylamine N-oxide (TMAO)
Biogenic amines	23	Acetylornithine, asymmetric dimethylarginine, total dimethylarginine, alpha-aminoadipic acid, carnosine, creatinine, dopa, dopamine, histamine, kynurenine, methionine sulfoxide, hydroxyproline (t4-OH-Pro), hydroxyproline (c4-OH-Pro), phenylethylamine, putrescine, sarcosine, serotonin, spermidine, spermine, diacetylspermine, taurine, tyramine, nitrotyrosine
Carboxylic acids	1	Homocysteine
Monosaccharides	1	Glucose
Organic acids	17	Lactic acid, beta-hydroxybutyric acid, alpha-ketoglutaric acid, citric acid, butyric acid, propionic acid, 3-(3-hydroxyphenyl)-3-hydroxypropionic acid (HPHPA), para-hydroxyhippuric acid, succinic acid, fumaric acid, pyruvic acid, isobutyric acid, hippuric acid, methylmalonic acid, homovanillic acid, indole acetic acid, uric acid
Lyso-phosphatidylcholines	14	LysoPC a C14:0/C16:0/C16:1/C17:0/C18:0/C18:1/C18:2/C20:3/C20:4/C24:0/C26:0/C26:1/C28:0/C28:1
Diacyl-phosphatidylcholines	8	PC aa C32:2/C36:0/C36:6/C38:0/C38:6/C40:1/C40:2/C40:6
Acyl-alkyl-phosphatidylcholines	2	PC ae C36:0/C40:6
Sphingomyelines	5	SM C16:0, SM C16:1, SM C18:0, SM C18:1, SM C20:2

Hydroxysphingomyelines	5	SM (OH) C14:1, SM (OH) C16:1, SM (OH) C22:1, SM (OH) C22:2, SM (OH) C24:1
Vitamins and cofactors	1	Choline
Total	143	

a: acyl; aa: acyl-acyl; ae: acyl-alkyl; Cx:y: where x is the number of carbons in the fatty acid side chain; y is the number of double bonds in the fatty acid side chain; DC: decarboxyl; M: methyl; OH: hydroxyl; PC: phophatidylcholine; SM: sphingomyelin.

Table S12. Formula of diets used in mouse dietary trial

	High Fat Diet (HFD)	HFD -2/3 BCAAs	HFD -2/3 BCAAs -2/3 Phe	HFD -2/3 BCAAs -2/3 Phe -2/3 Typ	Standard Chow diet (SCD)	SCD -2/3 BCAAs	SCD -2/3 BCAAs -2/3 Phe	SCD -2/3 BCAAs -2/3 Phe -2/3 Typ
Product #	A20101501	A20120801	A20120802	A20120803	A20101503	A20120804	A20120805	A20120806
Casein, Lactic	0	0	0	0	0	0	0	0
L-Cystine	4.2	4.4	4.4	4.5	4.2	4.4	4.4	4.5
**L-Isoleucine**	**7.5**	**2.5**	**2.5**	**2.5**	**7.5**	**2.5**	**2.5**	**2.5**
**L-Leucine**	**15.7**	**5.2**	**5.2**	**5.2**	**15.7**	**5.2**	**5.2**	**5.2**
L-Lysine	13.0	15.0	15.6	15.8	13.0	15.0	15.6	15.8
L-Methionine	5.0	5.8	6.0	6.1	5.0	5.8	6.0	6.1
**L-Phenylalanine**	**8.3**	**9.6**	**2.8**	**2.8**	**8.3**	**9.6**	**2.8**	**2.8**
L-Threonine	7.1	8.2	8.5	8.6	7.1	8.2	8.5	8.6
**L-Tryptophan**	**2.1**	**2.4**	**2.5**	**0.7**	**2.1**	**2.4**	**2.5**	**0.7**
**L-Valine**	**9.2**	**3.1**	**3.1**	**3.1**	**9.2**	**3.1**	**3.1**	**3.1**
L-Histidine-HCl-H2O	4.5	5.2	5.4	5.5	4.5	5.2	5.4	5.5
L-Alanine	5.0	5.8	6.0	6.1	5.0	5.8	6.0	6.1
L-Arginine	5.9	6.8	7.1	7.2	5.9	6.8	7.1	7.2
Asparagine	7.0	8.1	8.4	8.5	7.0	8.1	8.4	8.5
L-Aspartic Acid	5.0	5.8	6.0	6.1	5.0	5.8	6.0	6.1
Glutamine	17.0	19.6	20.5	20.7	17.0	19.6	20.5	20.7
L-Glutamic Acid	20.7	23.9	24.9	25.2	20.7	23.9	24.9	25.2
Glycine	3.0	3.5	3.6	3.7	3.0	3.5	3.6	3.7
L-Proline	17.6	20.3	21.2	21.4	17.6	20.3	21.2	21.4
L-Serine	9.9	11.4	11.9	12.1	9.9	11.4	11.9	12.1
L-Tyrosine	9.0	10.4	10.8	11.0	9.0	10.4	10.8	11.0
Total L-Amino Acids	176.7	177.0	176.4	176.8	176.7	177.0	176.4	176.8
Corn Starch	0	0	0	0	506.2	506.2	506.2	506.2
Maltodextrin 10	125	125	125	125	125	125	125	125
Sucrose	68.8	68.8	68.8	68.8	68.8	68.8	68.8	68.8
Cellulose, BW200	50	50	50	50	50	50	50	50
Soybean Oil	25	25	25	25	25	25	25	25
Lard	245	245	245	245	20	20	20	20
Mineral Mix S10026	10	10	10	10	10	10	10	10
DiCalcium Phosphate	13	13	13	13	13	13	13	13
Calcium Carbonate	5.5	5.5	5.5	5.5	5.5	5.5	5.5	5.5
Potassium Citrate, 1 H2O	16.5	16.5	16.5	16.5	16.5	16.5	16.5	16.5
Sodium Bicarbonate	7.5	7.5	7.5	7.5	7.5	7.5	7.5	7.5
Vitamin Mix V10001	10	10	10	10	10	10	10	10
Choline Bitartrate	2	2	2	2	2	2	2	2
FD&C Blue Dye #1	0.025	0.05	0.025	0	0	0	0.01	0
FD&C Yellow Dye #5	0.025	0	0	0	0.05	0.025	0.04	0.04
FD&C Red Dye #40	0	0	0.025	0.05	0	0.025	0	0.01
Total	755.05	755.35	754.75	755.15	1036.25	1036.55	1035.95	1036.35

gm								
Protein	176.7	177.0	176.4	176.8	176.7	177.0	176.4	176.8
Carbohydrate	203.8	203.8	203.8	203.8	710.0	710.0	710.0	710.0
Fat	270.0	270.0	270.0	270.0	45.0	45.0	45.0	45.0
Fiber	50.0	50.0	50.0	50.0	50.0	50.0	50.0	50.0
gm%								
Protein	23.4	23.4	23.4	23.4	17.1	17.1	17.0	17.1
Carbohydrate	27.0	27.0	27.0	27.0	68.5	68.5	68.5	68.5
Fat	35.8	35.7	35.8	35.8	4.3	4.3	4.3	4.3
Fiber	6.6	6.6	6.6	6.6	4.8	4.8	4.8	4.8
kcal								
Protein	707	708	706	707	707	708	706	707
Carbohydrate	815	815	815	815	2840	2840	2840	2840
Fat	2430	2430	2430	2430	405	405	405	405
Total	3952	3953	3951	3952	3952	3953	3951	3952
kcal%								
Protein	18	18	18	18	18	18	18	18
Carbohydrate	21	21	21	21	72	72	72	72
Fat	61	61	62	61	10	10	10	10
Total	100	100	100	100	100	100	100	100
kcal / gm	5.2	5.2	5.2	5.2	3.8	3.8	3.8	3.8

Supplementary figures

Figure S1. Forest plots for metabolites associated with BMI

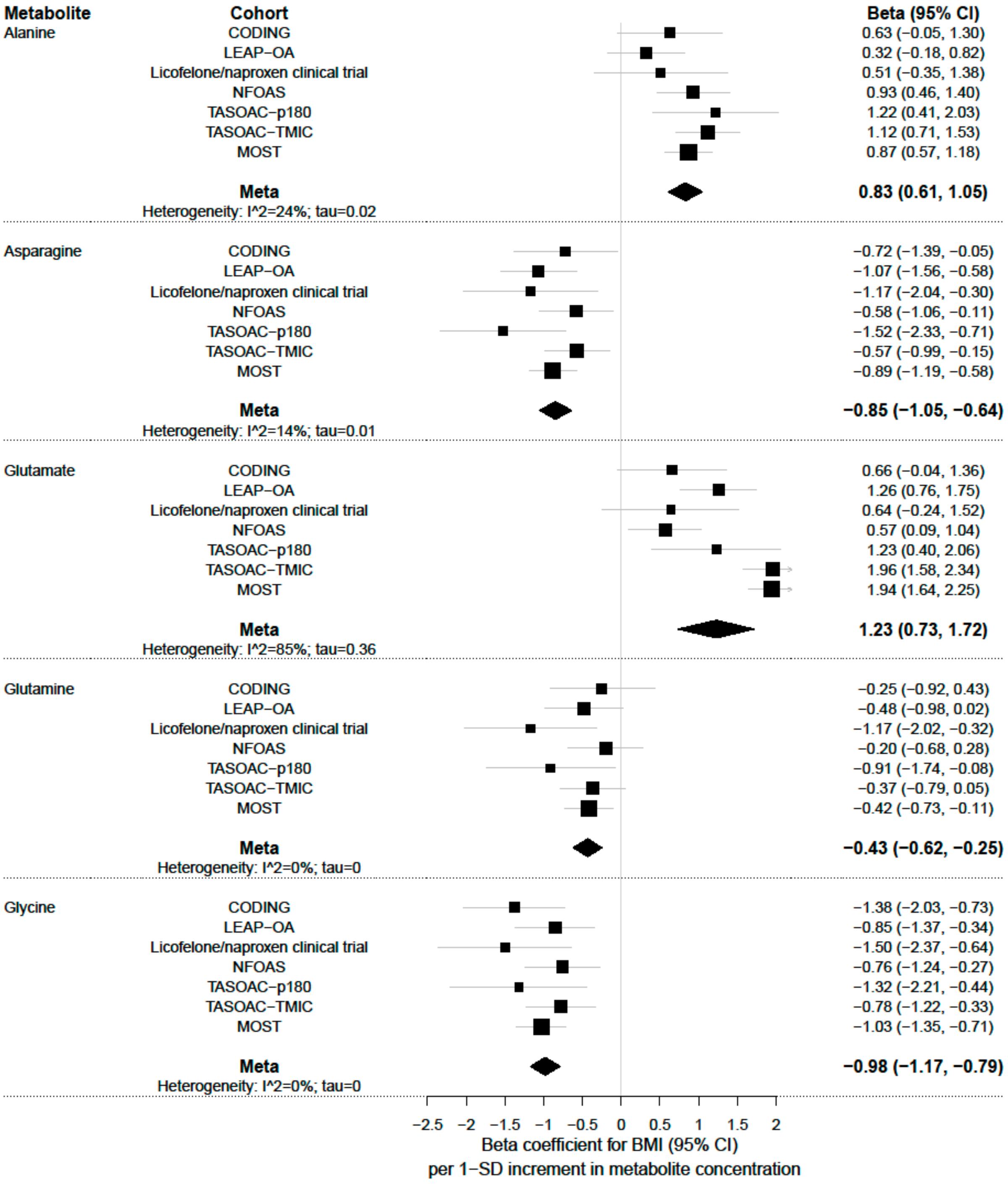

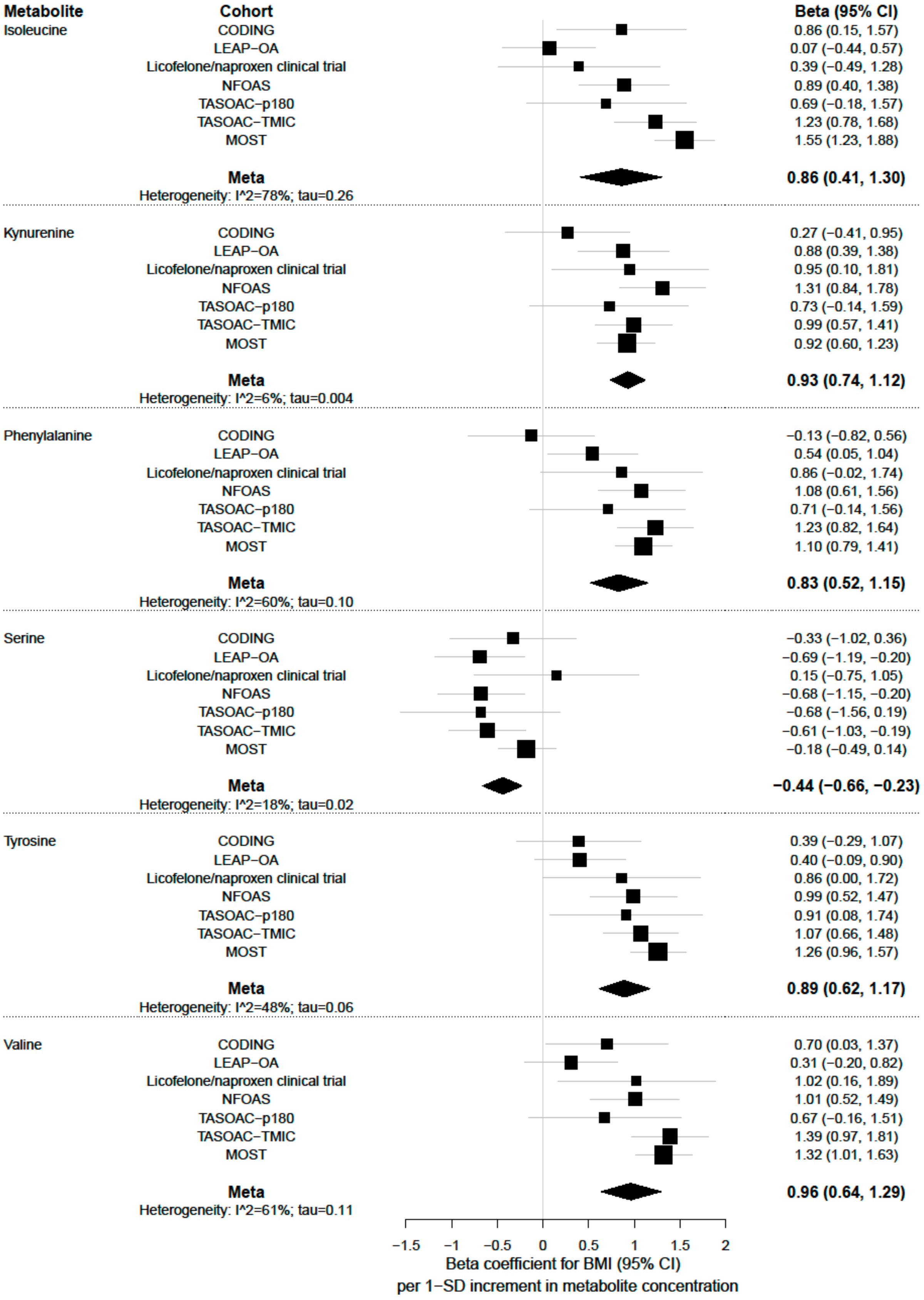
Metabolite
Cohort
Beta (95% CI)
Isoleucine
CODING
0.86 (0.15, 1.57)
LEAP−OA
0.07 (−0.44, 0.57)
Licofelone/naproxen clinical trial
0.39 (−0.49, 1.28)
NFOAS
0.89 (0.40, 1.38)
TASOAC−p180
0.69 (−0.18, 1.57)
TASOAC−TMIC
1.23 (0.78, 1.68)
MOST
1.55 (1.23, 1.88)
Meta
0.86 (0.41, 1.30)
Heterogeneity: I^2=78%; tau=0.26
Kynurenine
CODING
0.27 (−0.41, 0.95)
LEAP−OA
0.88 (0.39, 1.38)
Licofelone/naproxen clinical trial
0.95 (0.10, 1.81)
NFOAS
1.31 (0.84, 1.78)
TASOAC−p180
0.73 (−0.14, 1.59)
TASOAC−TMIC
0.99 (0.57, 1.41)
MOST
0.92 (0.60, 1.23)
Meta
0.93 (0.74, 1.12)
Heterogeneity: I^2=6%; tau=0.004
Phenylalanine
CODING
−0.13 (−0.82, 0.56)
LEAP−OA
0.54 (0.05, 1.04)
Licofelone/naproxen clinical trial
0.86 (−0.02, 1.74)
NFOAS
1.08 (0.61, 1.56)
TASOAC−p180
0.71 (−0.14, 1.56)
TASOAC−TMIC
1.23 (0.82, 1.64)
MOST
1.10 (0.79, 1.41)
Meta
0.83 (0.52, 1.15)
Heterogeneity: I^2=60%; tau=0.10
Serine
CODING
−0.33 (−1.02, 0.36)
LEAP−OA
−0.69 (−1.19, −0.20)
Licofelone/naproxen clinical trial
0.15 (−0.75, 1.05)
NFOAS
−0.68 (−1.15, −0.20)
TASOAC−p180
−0.68 (−1.56, 0.19)
TASOAC−TMIC
−0.61 (−1.03, −0.19)
MOST
−0.18 (−0.49, 0.14)
Meta
−0.44 (−0.66, −0.23)
Heterogeneity: I^2=18%; tau=0.02
Tyrosine
CODING
0.39 (−0.29, 1.07)
LEAP−OA
0.40 (−0.09, 0.90)
Licofelone/naproxen clinical trial
0.86 (0.00, 1.72)
NFOAS
0.99 (0.52, 1.47)
TASOAC−p180
0.91 (0.08, 1.74)
TASOAC−TMIC
1.07 (0.66, 1.48)
MOST
1.26 (0.96, 1.57)
Meta
0.89 (0.62, 1.17)
Heterogeneity: I^2=48%; tau=0.06
Valine
CODING
0.70 (0.03, 1.37)
LEAP−OA
0.31 (−0.20, 0.82)
Licofelone/naproxen clinical trial
1.02 (0.16, 1.89)
NFOAS
1.01 (0.52, 1.49)
TASOAC−p180
0.67 (−0.16, 1.51)
TASOAC−TMIC
1.39 (0.97, 1.81)
MOST
1.32 (1.01, 1.63)
Meta
0.96 (0.64, 1.29)
Heterogeneity: I^2=61%; tau=0.11
−1.5
−1
−0.5
0
0.5
1
1.5
2
Beta coefficient for BMI (95% CI)
per 1−SD increment in metabolite concentration

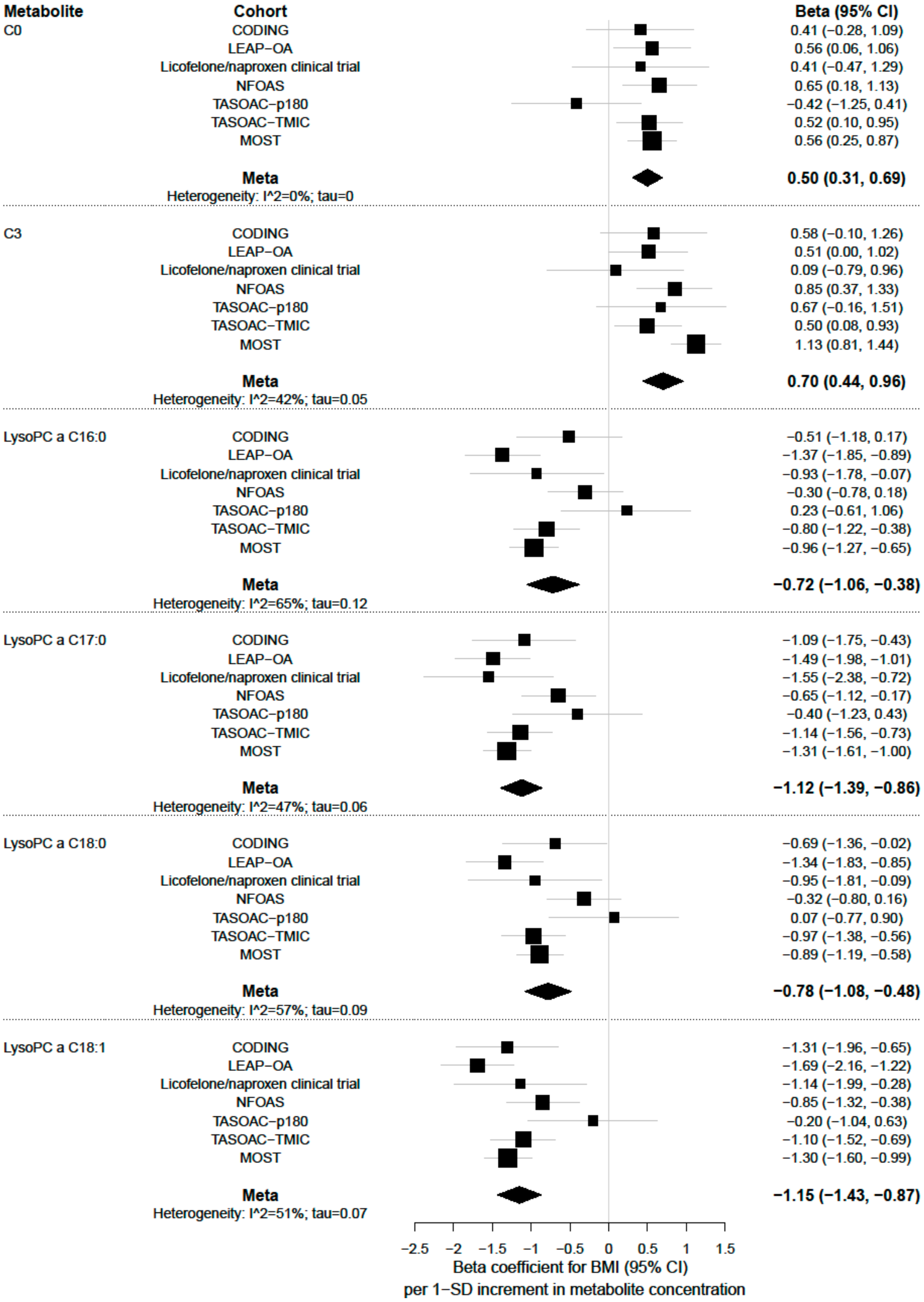
Metabolite
Cohort
Beta (95% CI)
C0
CODING
0.41 (−0.28, 1.09)
LEAP−OA
0.56 (0.06, 1.06)
Licofelone/naproxen clinical trial
0.41 (−0.47, 1.29)
NFOAS
0.65 (0.18, 1.13)
TASOAC−p180
−0.42 (−1.25, 0.41)
TASOAC−TMIC
0.52 (0.10, 0.95)
MOST
0.56 (0.25, 0.87)
Meta
0.50 (0.31, 0.69)
Heterogeneity: I^2=0%; tau=0
C3
CODING
0.58 (−0.10, 1.26)
LEAP−OA
0.51 (0.00, 1.02)
Licofelone/naproxen clinical trial
0.09 (−0.79, 0.96)
NFOAS
0.85 (0.37, 1.33)
TASOAC−p180
0.67 (−0.16, 1.51)
TASOAC−TMIC
0.50 (0.08, 0.93)
MOST
1.13 (0.81, 1.44)
Meta
0.70 (0.44, 0.96)
Heterogeneity: I^2=42%; tau=0.05
LysoPC a C16:0
CODING
−0.51 (−1.18, 0.17)
LEAP−OA
−1.37 (−1.85, −0.89)
Licofelone/naproxen clinical trial
−0.93 (−1.78, −0.07)
NFOAS
−0.30 (−0.78, 0.18)
TASOAC−p180
0.23 (−0.61, 1.06)
TASOAC−TMIC
−0.80 (−1.22, −0.38)
MOST
−0.96 (−1.27, −0.65)
Meta
−0.72 (−1.06, −0.38)
Heterogeneity: I^2=65%; tau=0.12
LysoPC a C17:0
CODING
−1.09 (−1.75, −0.43)
LEAP−OA
−1.49 (−1.98, −1.01)
Licofelone/naproxen clinical trial
−1.55 (−2.38, −0.72)
NFOAS
−0.65 (−1.12, −0.17)
TASOAC−p180
−0.40 (−1.23, 0.43)
TASOAC−TMIC
−1.14 (−1.56, −0.73)
MOST
−1.31 (−1.61, −1.00)
Meta
−1.12 (−1.39, −0.86)
Heterogeneity: I^2=47%; tau=0.06
LysoPC a C18:0
CODING
−0.69 (−1.36, −0.02)
LEAP−OA
−1.34 (−1.83, −0.85)
Licofelone/naproxen clinical trial
−0.95 (−1.81, −0.09)
NFOAS
−0.32 (−0.80, 0.16)
TASOAC−p180
0.07 (−0.77, 0.90)
TASOAC−TMIC
−0.97 (−1.38, −0.56)
MOST
−0.89 (−1.19, −0.58)
Meta
−0.78 (−1.08, −0.48)
Heterogeneity: I^2=57%; tau=0.09
LysoPC a C18:1
CODING
−1.31 (−1.96, −0.65)
LEAP−OA
−1.69 (−2.16, −1.22)
Licofelone/naproxen clinical trial
−1.14 (−1.99, −0.28)
NFOAS
−0.85 (−1.32, −0.38)
TASOAC−p180
−0.20 (−1.04, 0.63)
TASOAC−TMIC
−1.10 (−1.52, −0.69)
MOST
−1.30 (−1.60, −0.99)
Meta
−1.15 (−1.43, −0.87)
Heterogeneity: I^2=51%; tau=0.07
−2.5 −2 −1.5 −1 −0.5 0 0.5 1 1.5
Beta coefficient for BMI (95% CI)
per 1−SD increment in metabolite concentration

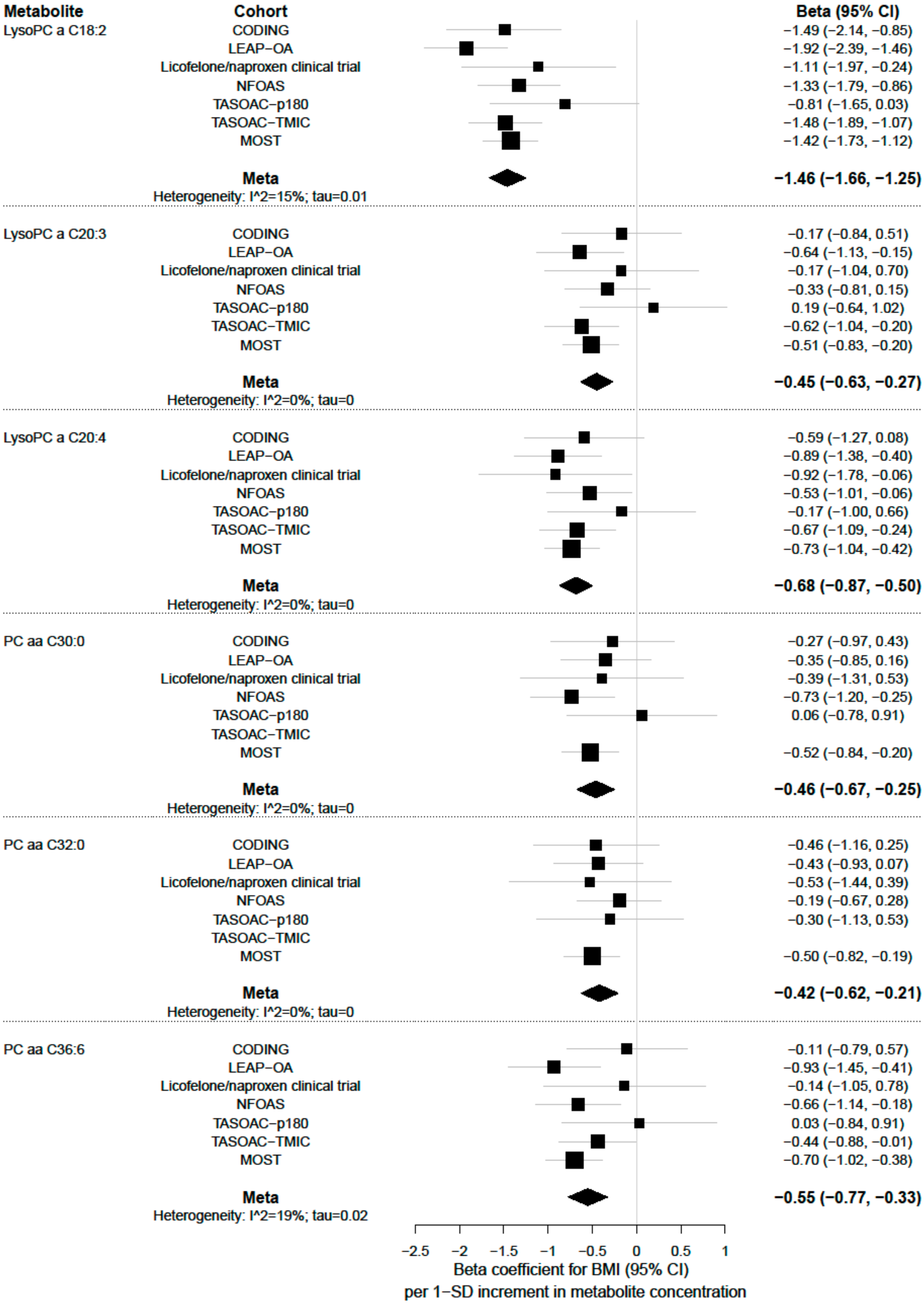
Metabolite
Cohort
Beta (95% CI)
LysoPC a C18:2
CODING −1.49 (−2.14, −0.85)
LEAP−OA −1.92 (−2.39, −1.46)
Licofelone/naproxen clinical trial −1.11 (−1.97, −0.24)
NFOAS −1.33 (−1.79, −0.86)
TASOAC−p180 −0.81 (−1.65, 0.03)
TASOAC−TMIC −1.48 (−1.89, −1.07)
MOST −1.42 (−1.73, −1.12)
Meta −1.46 (−1.66, −1.25)
Heterogeneity: I^2=15%; tau=0.01
LysoPC a C20:3
CODING −0.17 (−0.84, 0.51)
LEAP−OA −0.64 (−1.13, −0.15)
Licofelone/naproxen clinical trial −0.17 (−1.04, 0.70)
NFOAS −0.33 (−0.81, 0.15)
TASOAC−p180 0.19 (−0.64, 1.02)
TASOAC−TMIC −0.62 (−1.04, −0.20)
MOST −0.51 (−0.83, −0.20)
Meta −0.45 (−0.63, −0.27)
Heterogeneity: I^2=0%; tau=0
LysoPC a C20:4
CODING −0.59 (−1.27, 0.08)
LEAP−OA −0.89 (−1.38, −0.40)
Licofelone/naproxen clinical trial −0.92 (−1.78, −0.06)
NFOAS −0.53 (−1.01, −0.06)
TASOAC−p180 −0.17 (−1.00, 0.66)
TASOAC−TMIC −0.67 (−1.09, −0.24)
MOST −0.73 (−1.04, −0.42)
Meta −0.68 (−0.87, −0.50)
Heterogeneity: I^2=0%; tau=0
PC aa C30:0
CODING −0.27 (−0.97, 0.43)
LEAP−OA −0.35 (−0.85, 0.16)
Licofelone/naproxen clinical trial −0.39 (−1.31, 0.53)
NFOAS −0.73 (−1.20, −0.25)
TASOAC−p180 0.06 (−0.78, 0.91)
TASOAC−TMIC
MOST −0.52 (−0.84, −0.20)
Meta −0.46 (−0.67, −0.25)
Heterogeneity: I^2=0%; tau=0
PC aa C32:0
CODING −0.46 (−1.16, 0.25)
LEAP−OA −0.43 (−0.93, 0.07)
Licofelone/naproxen clinical trial −0.53 (−1.44, 0.39)
NFOAS −0.19 (−0.67, 0.28)
TASOAC−p180 −0.30 (−1.13, 0.53)
TASOAC−TMIC
MOST −0.50 (−0.82, −0.19)
Meta −0.42 (−0.62, −0.21)
Heterogeneity: I^2=0%; tau=0
PC aa C36:6
CODING −0.11 (−0.79, 0.57)
LEAP−OA −0.93 (−1.45, −0.41)
Licofelone/naproxen clinical trial −0.14 (−1.05, 0.78)
NFOAS −0.66 (−1.14, −0.18)
TASOAC−p180 0.03 (−0.84, 0.91)
TASOAC−TMIC −0.44 (−0.88, −0.01)
MOST −0.70 (−1.02, −0.38)
Meta −0.55 (−0.77, −0.33)
Heterogeneity: I^2=19%; tau=0.02
−2.5 −2 −1.5 −1 −0.5 0 0.5 1
Beta coefficient for BMI (95% CI)
per 1−SD increment in metabolite concentration

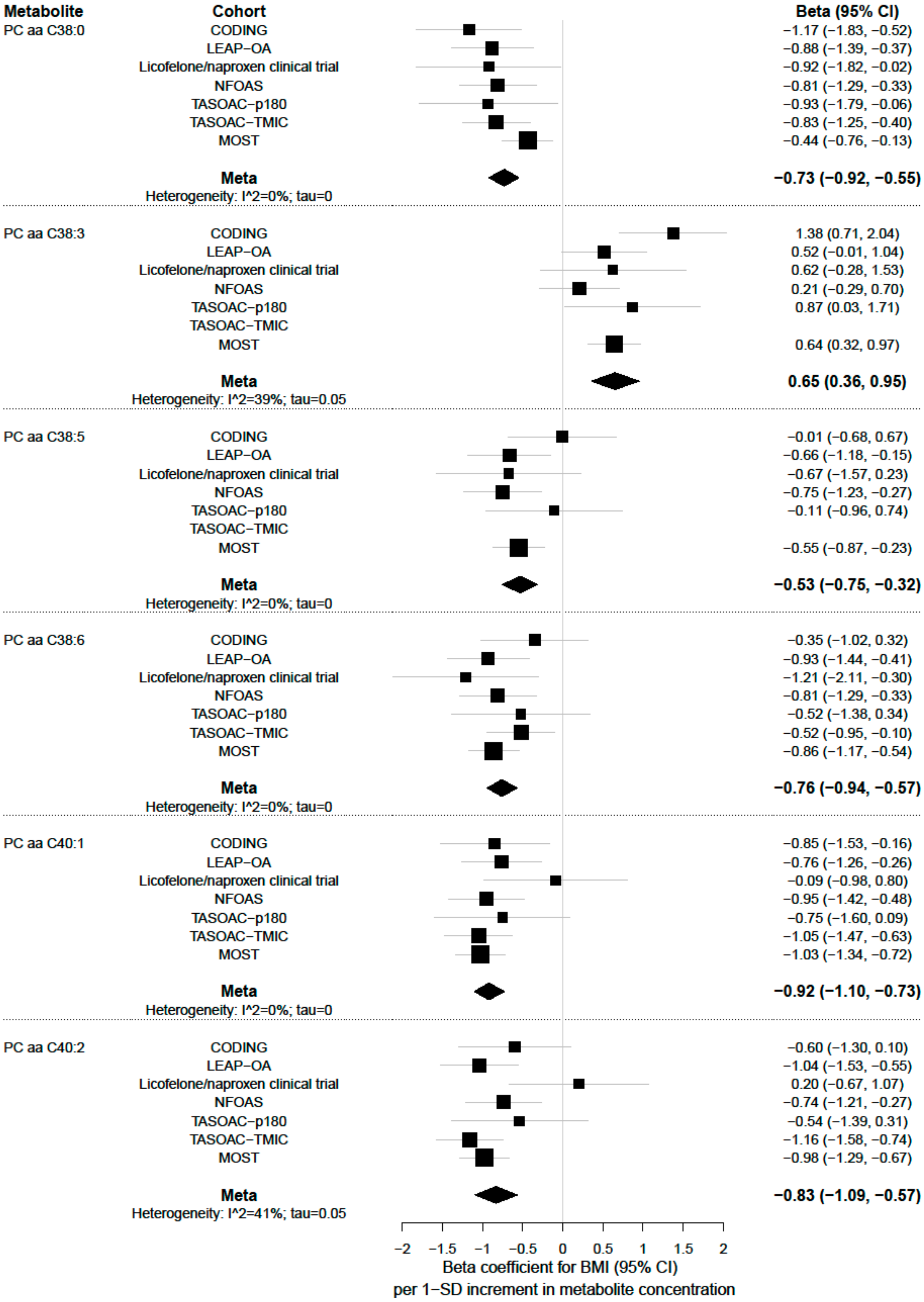
Metabolite
Cohort
Beta (95% CI)
PC aa C38:0
CODING −1.17 (−1.83, −0.52)
LEAP−OA −0.88 (−1.39, −0.37)
Licofelone/naproxen clinical trial −0.92 (−1.82, −0.02)
NFOAS −0.81 (−1.29, −0.33)
TASOAC−p180 −0.93 (−1.79, −0.06)
TASOAC−TMIC −0.83 (−1.25, −0.40)
MOST −0.44 (−0.76, −0.13)
Meta −0.73 (−0.92, −0.55)
Heterogeneity: I^2=0%; tau=0
PC aa C38:3
CODING 1.38 (0.71, 2.04)
LEAP−OA 0.52 (−0.01, 1.04)
Licofelone/naproxen clinical trial 0.62 (−0.28, 1.53)
NFOAS 0.21 (−0.29, 0.70)
TASOAC−p180 0.87 (0.03, 1.71)
TASOAC−TMIC
MOST 0.64 (0.32, 0.97)
Meta 0.65 (0.36, 0.95)
Heterogeneity: I^2=39%; tau=0.05
PC aa C38:5
CODING −0.01 (−0.68, 0.67)
LEAP−OA −0.66 (−1.18, −0.15)
Licofelone/naproxen clinical trial −0.67 (−1.57, 0.23)
NFOAS −0.75 (−1.23, −0.27)
TASOAC−p180 −0.11 (−0.96, 0.74)
TASOAC−TMIC
MOST −0.55 (−0.87, −0.23)
Meta −0.53 (−0.75, −0.32)
Heterogeneity: I^2=0%; tau=0
PC aa C38:6
CODING −0.35 (−1.02, 0.32)
LEAP−OA −0.93 (−1.44, −0.41)
Licofelone/naproxen clinical trial −1.21 (−2.11, −0.30)
NFOAS −0.81 (−1.29, −0.33)
TASOAC−p180 −0.52 (−1.38, 0.34)
TASOAC−TMIC −0.52 (−0.95, −0.10)
MOST −0.86 (−1.17, −0.54)
Meta −0.76 (−0.94, −0.57)
Heterogeneity: I^2=0%; tau=0
PC aa C40:1
CODING −0.85 (−1.53, −0.16)
LEAP−OA −0.76 (−1.26, −0.26)
Licofelone/naproxen clinical trial −0.09 (−0.98, 0.80)
NFOAS −0.95 (−1.42, −0.48)
TASOAC−p180 −0.75 (−1.60, 0.09)
TASOAC−TMIC −1.05 (−1.47, −0.63)
MOST −1.03 (−1.34, −0.72)
Meta −0.92 (−1.10, −0.73)
Heterogeneity: I^2=0%; tau=0
PC aa C40:2
CODING −0.60 (−1.30, 0.10)
LEAP−OA −1.04 (−1.53, −0.55)
Licofelone/naproxen clinical trial 0.20 (−0.67, 1.07)
NFOAS −0.74 (−1.21, −0.27)
TASOAC−p180 −0.54 (−1.39, 0.31)
TASOAC−TMIC −1.16 (−1.58, −0.74)
MOST −0.98 (−1.29, −0.67)
Meta −0.83 (−1.09, −0.57)
Heterogeneity: I^2=41%; tau=0.05
−2 −1.5 −1 −0.5 0 0.5 1 1.5 2
Beta coefficient for BMI (95% CI)
per 1−SD increment in metabolite concentration

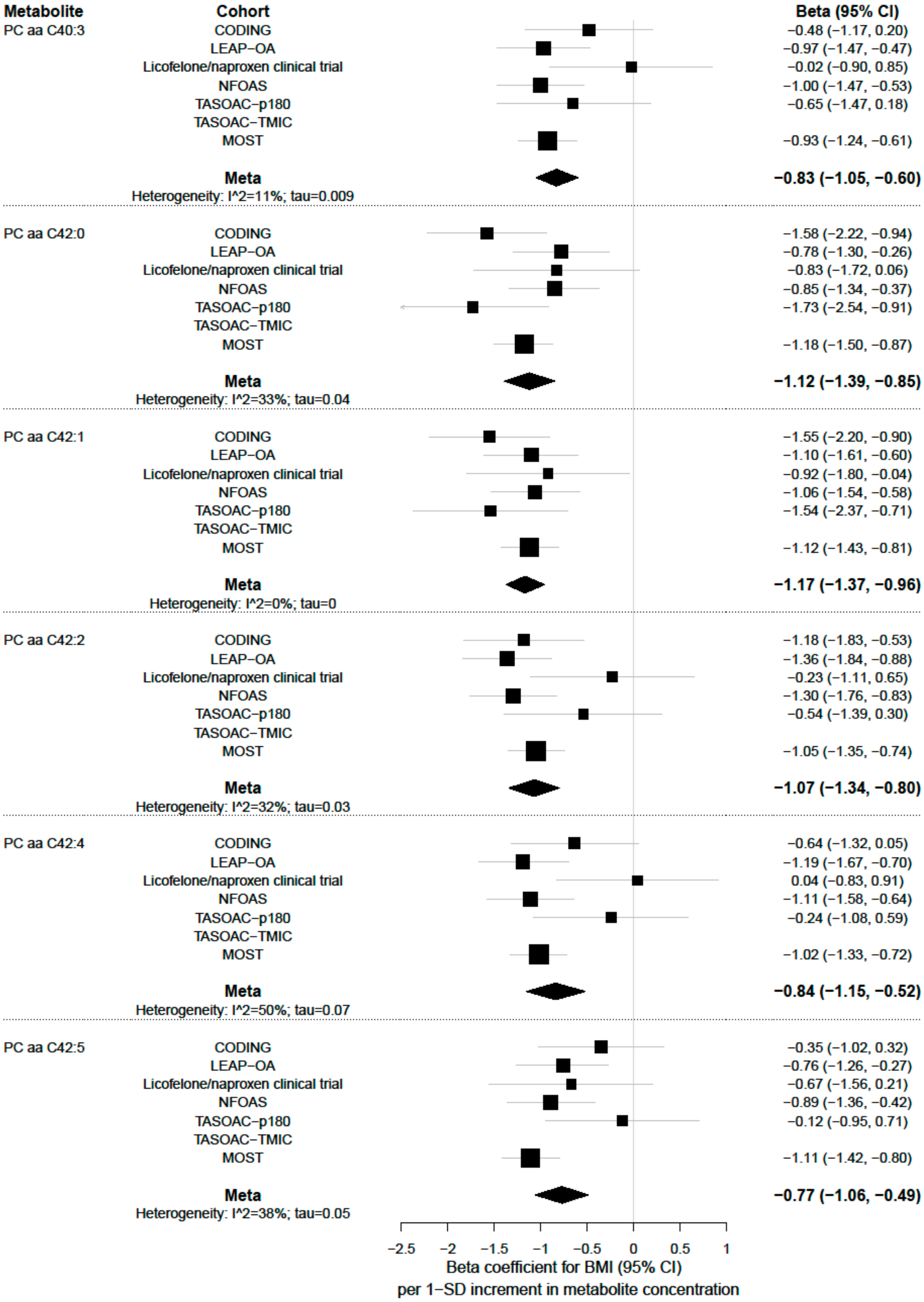
Metabolite
Cohort
Beta (95% CI)
PC aa C40:3
CODING
LEAP−OA
Licofelone/naproxen clinical trial
NFOAS
TASOAC−p180
TASOAC−TMIC
MOST
Meta
Heterogeneity: I^2=11%; tau=0.009
−0.48 (−1.17, 0.20)
−0.97 (−1.47, −0.47)
−0.02 (−0.90, 0.85)
−1.00 (−1.47, −0.53)
−0.65 (−1.47, 0.18)
−0.93 (−1.24, −0.61)
−0.83 (−1.05, −0.60)
PC aa C42:0
CODING
LEAP−OA
Licofelone/naproxen clinical trial
NFOAS
TASOAC−p180
TASOAC−TMIC
MOST
Meta
Heterogeneity: I^2=33%; tau=0.04
−1.58 (−2.22, −0.94)
−0.78 (−1.30, −0.26)
−0.83 (−1.72, 0.06)
−0.85 (−1.34, −0.37)
−1.73 (−2.54, −0.91)
−1.18 (−1.50, −0.87)
−1.12 (−1.39, −0.85)
PC aa C42:1
CODING
LEAP−OA
Licofelone/naproxen clinical trial
NFOAS
TASOAC−p180
TASOAC−TMIC
MOST
Meta
Heterogeneity: I^2=0%; tau=0
−1.55 (−2.20, −0.90)
−1.10 (−1.61, −0.60)
−0.92 (−1.80, −0.04)
−1.06 (−1.54, −0.58)
−1.54 (−2.37, −0.71)
−1.12 (−1.43, −0.81)
−1.17 (−1.37, −0.96)
PC aa C42:2
CODING
LEAP−OA
Licofelone/naproxen clinical trial
NFOAS
TASOAC−p180
TASOAC−TMIC
MOST
Meta
Heterogeneity: I^2=32%; tau=0.03
−1.18 (−1.83, −0.53)
−1.36 (−1.84, −0.88)
−0.23 (−1.11, 0.65)
−1.30 (−1.76, −0.83)
−0.54 (−1.39, 0.30)
−1.05 (−1.35, −0.74)
−1.07 (−1.34, −0.80)
PC aa C42:4
CODING
LEAP−OA
Licofelone/naproxen clinical trial
NFOAS
TASOAC−p180
TASOAC−TMIC
MOST
Meta
Heterogeneity: I^2=50%; tau=0.07
−0.64 (−1.32, 0.05)
−1.19 (−1.67, −0.70)
0.04 (−0.83, 0.91)
−1.11 (−1.58, −0.64)
−0.24 (−1.08, 0.59)
−1.02 (−1.33, −0.72)
−0.84 (−1.15, −0.52)
PC aa C42:5
CODING
LEAP−OA
Licofelone/naproxen clinical trial
NFOAS
TASOAC−p180
TASOAC−TMIC
MOST
Meta
Heterogeneity: I^2=38%; tau=0.05
−0.35 (−1.02, 0.32)
−0.76 (−1.26, −0.27)
−0.67 (−1.56, 0.21)
−0.89 (−1.36, −0.42)
−0.12 (−0.95, 0.71)
−1.11 (−1.42, −0.80)
−0.77 (−1.06, −0.49)
−2.5
−2
−1.5
−1
−0.5
0
0.5
1
Beta coefficient for BMI (95% CI)
per 1−SD increment in metabolite concentration

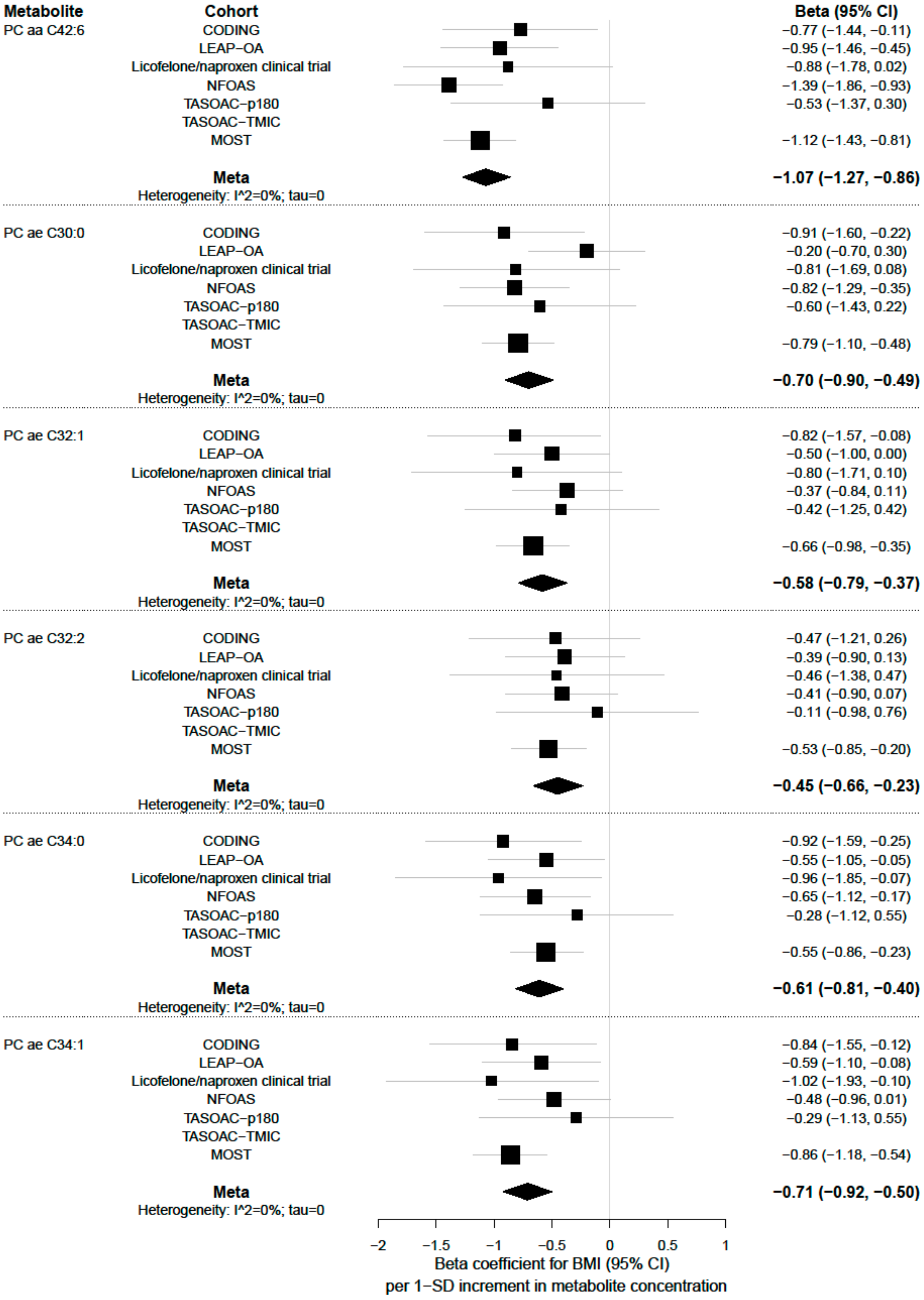
Metabolite
Cohort
Beta (95% CI)
PC aa C42:6
CODING −0.77 (−1.44, −0.11)
LEAP−OA −0.95 (−1.46, −0.45)
Licofelone/naproxen clinical trial −0.88 (−1.78, 0.02)
NFOAS −1.39 (−1.86, −0.93)
TASOAC−p180 −0.53 (−1.37, 0.30)
TASOAC−TMIC
MOST −1.12 (−1.43, −0.81)
Meta −1.07 (−1.27, −0.86)
Heterogeneity: I^2=0%; tau=0
PC ae C30:0
CODING −0.91 (−1.60, −0.22)
LEAP−OA −0.20 (−0.70, 0.30)
Licofelone/naproxen clinical trial −0.81 (−1.69, 0.08)
NFOAS −0.82 (−1.29, −0.35)
TASOAC−p180 −0.60 (−1.43, 0.22)
TASOAC−TMIC
MOST −0.79 (−1.10, −0.48)
Meta −0.70 (−0.90, −0.49)
Heterogeneity: I^2=0%; tau=0
PC ae C32:1
CODING −0.82 (−1.57, −0.08)
LEAP−OA −0.50 (−1.00, 0.00)
Licofelone/naproxen clinical trial −0.80 (−1.71, 0.10)
NFOAS −0.37 (−0.84, 0.11)
TASOAC−p180 −0.42 (−1.25, 0.42)
TASOAC−TMIC
MOST −0.66 (−0.98, −0.35)
Meta −0.58 (−0.79, −0.37)
Heterogeneity: I^2=0%; tau=0
PC ae C32:2
CODING −0.47 (−1.21, 0.26)
LEAP−OA −0.39 (−0.90, 0.13)
Licofelone/naproxen clinical trial −0.46 (−1.38, 0.47)
NFOAS −0.41 (−0.90, 0.07)
TASOAC−p180 −0.11 (−0.98, 0.76)
TASOAC−TMIC
MOST −0.53 (−0.85, −0.20)
Meta −0.45 (−0.66, −0.23)
Heterogeneity: I^2=0%; tau=0
PC ae C34:0
CODING −0.92 (−1.59, −0.25)
LEAP−OA −0.55 (−1.05, −0.05)
Licofelone/naproxen clinical trial −0.96 (−1.85, −0.07)
NFOAS −0.65 (−1.12, −0.17)
TASOAC−p180 −0.28 (−1.12, 0.55)
TASOAC−TMIC
MOST −0.55 (−0.86, −0.23)
Meta −0.61 (−0.81, −0.40)
Heterogeneity: I^2=0%; tau=0
PC ae C34:1
CODING −0.84 (−1.55, −0.12)
LEAP−OA −0.59 (−1.10, −0.08)
Licofelone/naproxen clinical trial −1.02 (−1.93, −0.10)
NFOAS −0.48 (−0.96, 0.01)
TASOAC−p180 −0.29 (−1.13, 0.55)
TASOAC−TMIC
MOST −0.86 (−1.18, −0.54)
Meta −0.71 (−0.92, −0.50)
Heterogeneity: I^2=0%; tau=0
−2 −1.5 −1 −0.5 0 0.5 1
Beta coefficient for BMI (95% CI)
per 1−SD increment in metabolite concentration

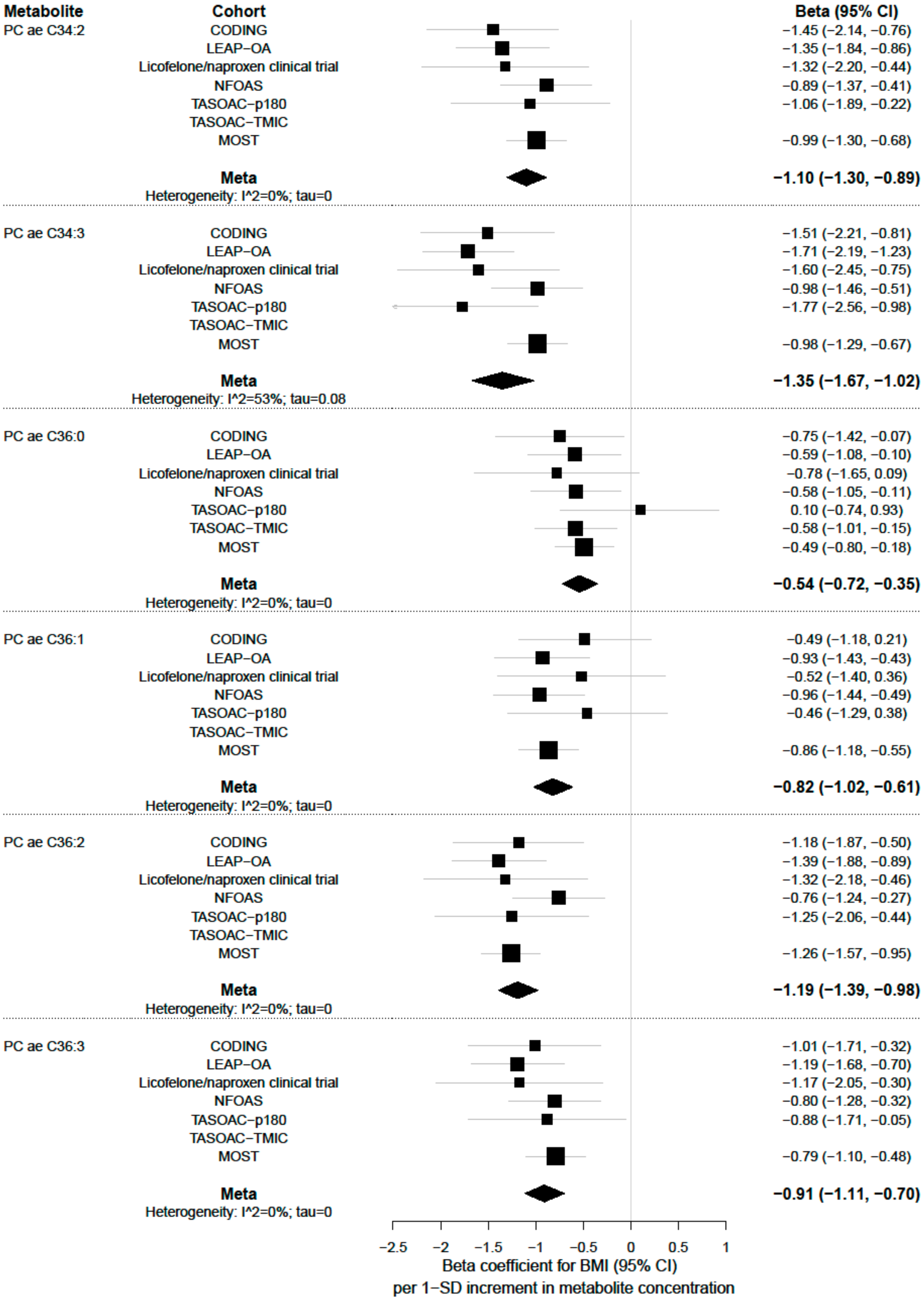
Metabolite
Cohort
Beta (95% CI)
PC ae C34:2
CODING −1.45 (−2.14, −0.76)
LEAP−OA −1.35 (−1.84, −0.86)
Licofelone/naproxen clinical trial −1.32 (−2.20, −0.44)
NFOAS −0.89 (−1.37, −0.41)
TASOAC−p180 −1.06 (−1.89, −0.22)
TASOAC−TMIC
MOST −0.99 (−1.30, −0.68)
Meta −1.10 (−1.30, −0.89)
Heterogeneity: I^2=0%; tau=0
PC ae C34:3
CODING −1.51 (−2.21, −0.81)
LEAP−OA −1.71 (−2.19, −1.23)
Licofelone/naproxen clinical trial −1.60 (−2.45, −0.75)
NFOAS −0.98 (−1.46, −0.51)
TASOAC−p180 −1.77 (−2.56, −0.98)
TASOAC−TMIC
MOST −0.98 (−1.29, −0.67)
Meta −1.35 (−1.67, −1.02)
Heterogeneity: I^2=53%; tau=0.08
PC ae C36:0
CODING −0.75 (−1.42, −0.07)
LEAP−OA −0.59 (−1.08, −0.10)
Licofelone/naproxen clinical trial −0.78 (−1.65, 0.09)
NFOAS −0.58 (−1.05, −0.11)
TASOAC−p180 0.10 (−0.74, 0.93)
TASOAC−TMIC −0.58 (−1.01, −0.15)
MOST −0.49 (−0.80, −0.18)
Meta −0.54 (−0.72, −0.35)
Heterogeneity: I^2=0%; tau=0
PC ae C36:1
CODING −0.49 (−1.18, 0.21)
LEAP−OA −0.93 (−1.43, −0.43)
Licofelone/naproxen clinical trial −0.52 (−1.40, 0.36)
NFOAS −0.96 (−1.44, −0.49)
TASOAC−p180 −0.46 (−1.29, 0.38)
TASOAC−TMIC
MOST −0.86 (−1.18, −0.55)
Meta −0.82 (−1.02, −0.61)
Heterogeneity: I^2=0%; tau=0
PC ae C36:2
CODING −1.18 (−1.87, −0.50)
LEAP−OA −1.39 (−1.88, −0.89)
Licofelone/naproxen clinical trial −1.32 (−2.18, −0.46)
NFOAS −0.76 (−1.24, −0.27)
TASOAC−p180 −1.25 (−2.06, −0.44)
TASOAC−TMIC
MOST −1.26 (−1.57, −0.95)
Meta −1.19 (−1.39, −0.98)
Heterogeneity: I^2=0%; tau=0
PC ae C36:3
CODING −1.01 (−1.71, −0.32)
LEAP−OA −1.19 (−1.68, −0.70)
Licofelone/naproxen clinical trial −1.17 (−2.05, −0.30)
NFOAS −0.80 (−1.28, −0.32)
TASOAC−p180 −0.88 (−1.71, −0.05)
TASOAC−TMIC
MOST −0.79 (−1.10, −0.48)
Meta −0.91 (−1.11, −0.70)
Heterogeneity: I^2=0%; tau=0
−2.5 −2 −1.5 −1 −0.5 0 0.5 1
Beta coefficient for BMI (95% CI)
per 1−SD increment in metabolite concentration

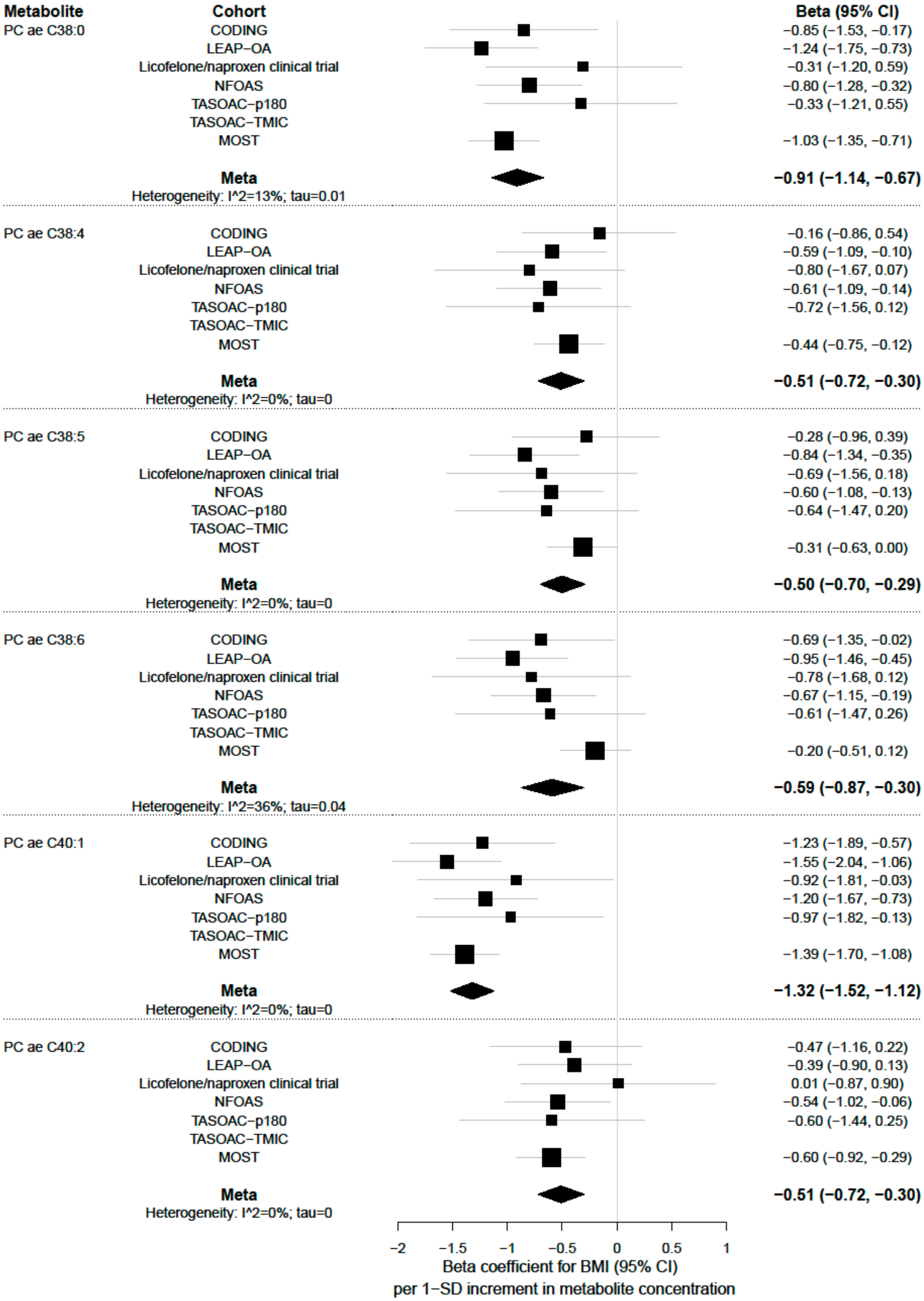
Metabolite
Cohort
Beta (95% CI)
PC ae C38:0
CODING
LEAP−OA
Licofelone/naproxen clinical trial
NFOAS
TASOAC−p180
TASOAC−TMIC
MOST
Meta
Heterogeneity: I^2=13%; tau=0.01
−0.85 (−1.53, −0.17)
−1.24 (−1.75, −0.73)
−0.31 (−1.20, 0.59)
−0.80 (−1.28, −0.32)
−0.33 (−1.21, 0.55)
−1.03 (−1.35, −0.71)
−0.91 (−1.14, −0.67)
PC ae C38:4
CODING
LEAP−OA
Licofelone/naproxen clinical trial
NFOAS
TASOAC−p180
TASOAC−TMIC
MOST
Meta
Heterogeneity: I^2=0%; tau=0
−0.16 (−0.86, 0.54)
−0.59 (−1.09, −0.10)
−0.80 (−1.67, 0.07)
−0.61 (−1.09, −0.14)
−0.72 (−1.56, 0.12)
−0.44 (−0.75, −0.12)
−0.51 (−0.72, −0.30)
PC ae C38:5
CODING
LEAP−OA
Licofelone/naproxen clinical trial
NFOAS
TASOAC−p180
TASOAC−TMIC
MOST
Meta
Heterogeneity: I^2=0%; tau=0
−0.28 (−0.96, 0.39)
−0.84 (−1.34, −0.35)
−0.69 (−1.56, 0.18)
−0.60 (−1.08, −0.13)
−0.64 (−1.47, 0.20)
−0.31 (−0.63, 0.00)
−0.50 (−0.70, −0.29)
PC ae C38:6
CODING
LEAP−OA
Licofelone/naproxen clinical trial
NFOAS
TASOAC−p180
TASOAC−TMIC
MOST
Meta
Heterogeneity: I^2=36%; tau=0.04
−0.69 (−1.35, −0.02)
−0.95 (−1.46, −0.45)
−0.78 (−1.68, 0.12)
−0.67 (−1.15, −0.19)
−0.61 (−1.47, 0.26)
−0.20 (−0.51, 0.12)
−0.59 (−0.87, −0.30)
PC ae C40:1
CODING
LEAP−OA
Licofelone/naproxen clinical trial
NFOAS
TASOAC−p180
TASOAC−TMIC
MOST
Meta
Heterogeneity: I^2=0%; tau=0
−1.23 (−1.89, −0.57)
−1.55 (−2.04, −1.06)
−0.92 (−1.81, −0.03)
−1.20 (−1.67, −0.73)
−0.97 (−1.82, −0.13)
−1.39 (−1.70, −1.08)
−1.32 (−1.52, −1.12)
PC ae C40:2
CODING
LEAP−OA
Licofelone/naproxen clinical trial
NFOAS
TASOAC−p180
TASOAC−TMIC
MOST
Meta
Heterogeneity: I^2=0%; tau=0
−0.47 (−1.16, 0.22)
−0.39 (−0.90, 0.13)
0.01 (−0.87, 0.90)
−0.54 (−1.02, −0.06)
−0.60 (−1.44, 0.25)
−0.60 (−0.92, −0.29)
−0.51 (−0.72, −0.30)
−2
−1.5
−1
−0.5
0
0.5
1
Beta coefficient for BMI (95% CI)
per 1−SD increment in metabolite concentration

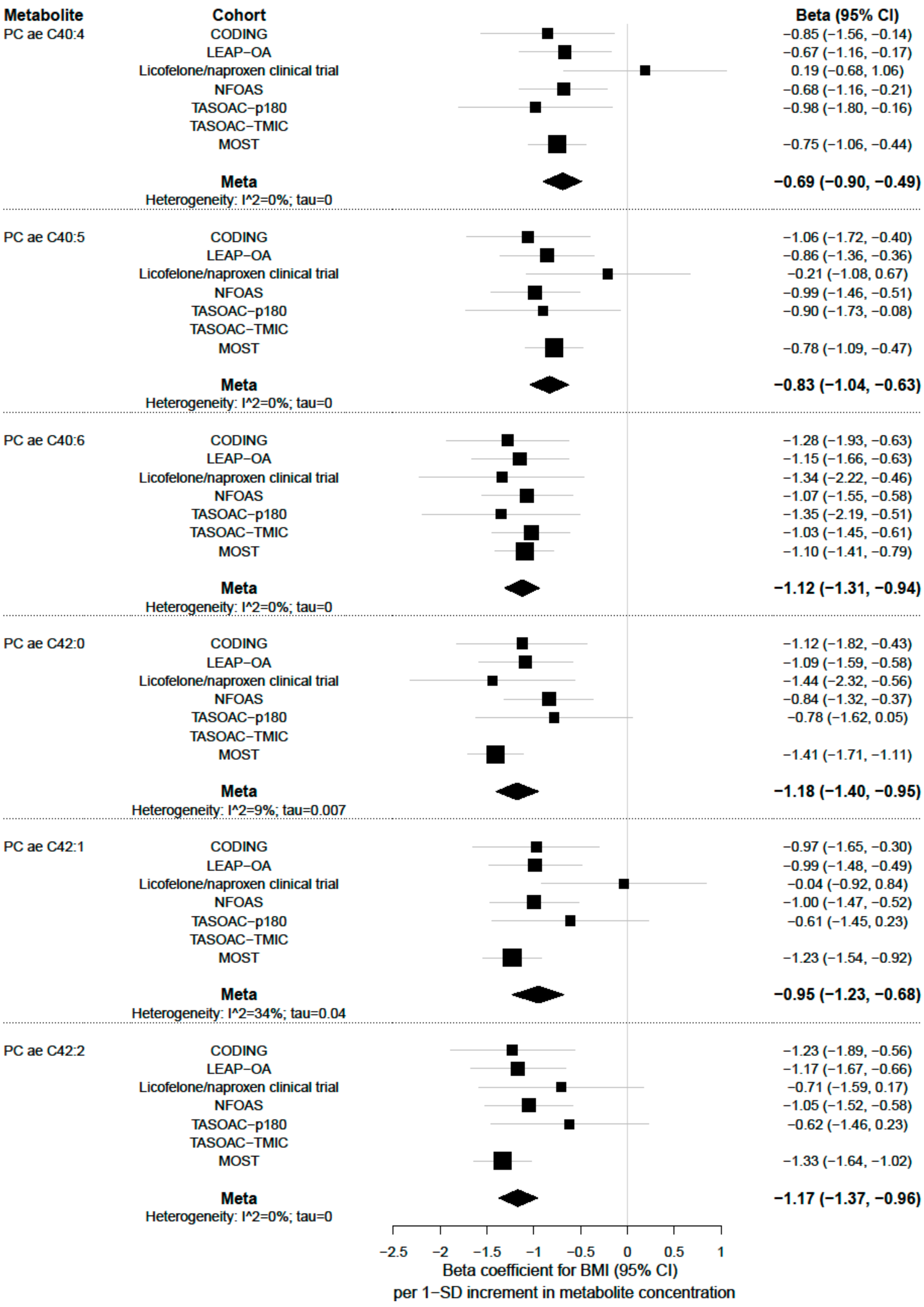
Metabolite
Cohort
Beta (95% CI)
PC ae C40:4
CODING −0.85 (−1.56, −0.14)
LEAP−OA −0.67 (−1.16, −0.17)
Licofelone/naproxen clinical trial 0.19 (−0.68, 1.06)
NFOAS −0.68 (−1.16, −0.21)
TASOAC−p180 −0.98 (−1.80, −0.16)
TASOAC−TMIC
MOST −0.75 (−1.06, −0.44)
Meta −0.69 (−0.90, −0.49)
Heterogeneity: I^2=0%; tau=0
PC ae C40:5
CODING −1.06 (−1.72, −0.40)
LEAP−OA −0.86 (−1.36, −0.36)
Licofelone/naproxen clinical trial −0.21 (−1.08, 0.67)
NFOAS −0.99 (−1.46, −0.51)
TASOAC−p180 −0.90 (−1.73, −0.08)
TASOAC−TMIC
MOST −0.78 (−1.09, −0.47)
Meta −0.83 (−1.04, −0.63)
Heterogeneity: I^2=0%; tau=0
PC ae C40:6
CODING −1.28 (−1.93, −0.63)
LEAP−OA −1.15 (−1.66, −0.63)
Licofelone/naproxen clinical trial −1.34 (−2.22, −0.46)
NFOAS −1.07 (−1.55, −0.58)
TASOAC−p180 −1.35 (−2.19, −0.51)
TASOAC−TMIC −1.03 (−1.45, −0.61)
MOST −1.10 (−1.41, −0.79)
Meta −1.12 (−1.31, −0.94)
Heterogeneity: I^2=0%; tau=0
PC ae C42:0
CODING −1.12 (−1.82, −0.43)
LEAP−OA −1.09 (−1.59, −0.58)
Licofelone/naproxen clinical trial −1.44 (−2.32, −0.56)
NFOAS −0.84 (−1.32, −0.37)
TASOAC−p180 −0.78 (−1.62, 0.05)
TASOAC−TMIC
MOST −1.41 (−1.71, −1.11)
Meta −1.18 (−1.40, −0.95)
Heterogeneity: I^2=9%; tau=0.007
PC ae C42:1
CODING −0.97 (−1.65, −0.30)
LEAP−OA −0.99 (−1.48, −0.49)
Licofelone/naproxen clinical trial −0.04 (−0.92, 0.84)
NFOAS −1.00 (−1.47, −0.52)
TASOAC−p180 −0.61 (−1.45, 0.23)
TASOAC−TMIC
MOST −1.23 (−1.54, −0.92)
Meta −0.95 (−1.23, −0.68)
Heterogeneity: I^2=34%; tau=0.04
PC ae C42:2
CODING −1.23 (−1.89, −0.56)
LEAP−OA −1.17 (−1.67, −0.66)
Licofelone/naproxen clinical trial −0.71 (−1.59, 0.17)
NFOAS −1.05 (−1.52, −0.58)
TASOAC−p180 −0.62 (−1.46, 0.23)
TASOAC−TMIC
MOST −1.33 (−1.64, −1.02)
Meta −1.17 (−1.37, −0.96)
Heterogeneity: I^2=0%; tau=0
−2.5 −2 −1.5 −1 −0.5 0 0.5 1
Beta coefficient for BMI (95% CI)
per 1−SD increment in metabolite concentration

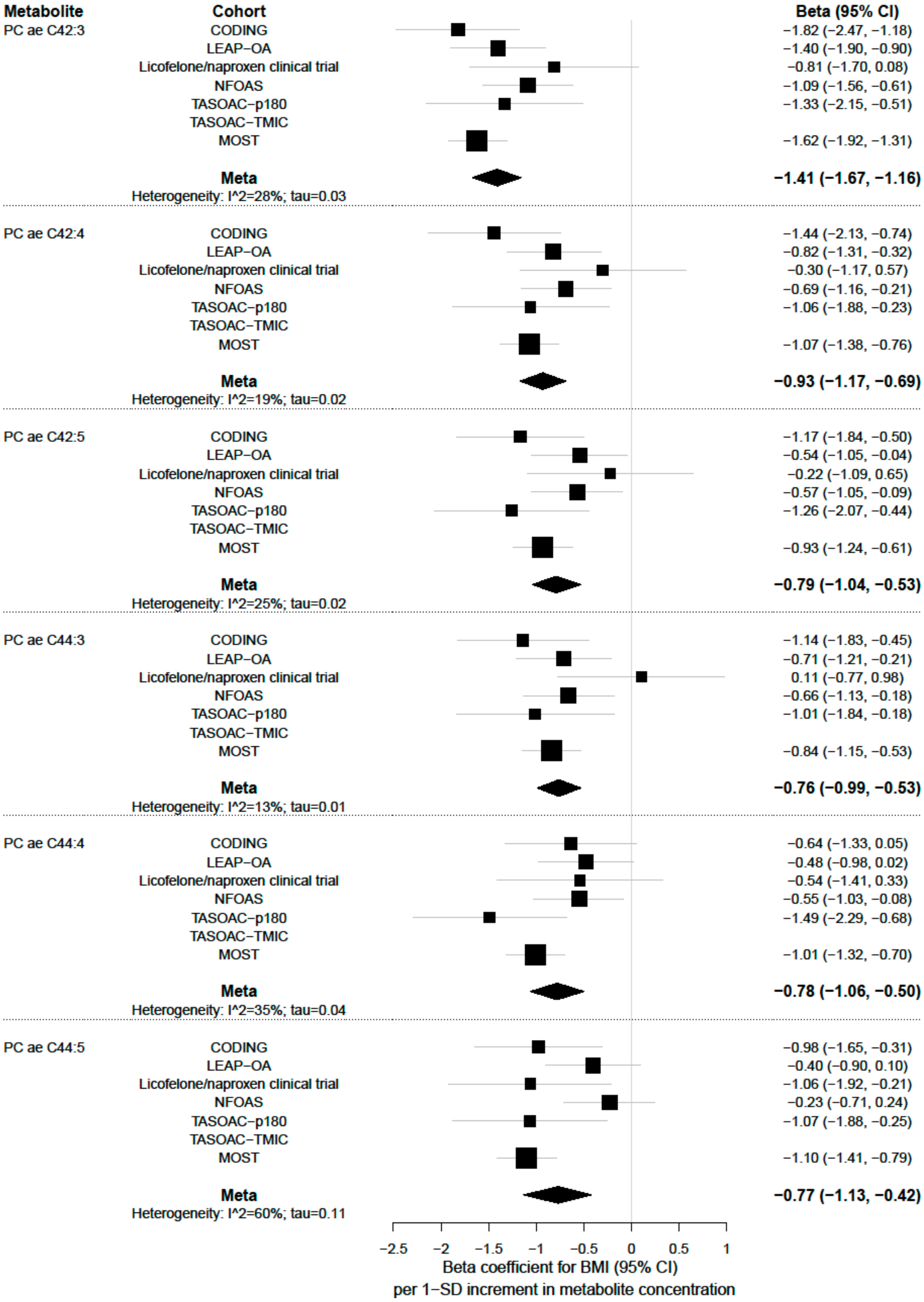
Metabolite
Cohort
Beta (95% CI)
PC ae C42:3
CODING −1.82 (−2.47, −1.18)
LEAP−OA −1.40 (−1.90, −0.90)
Licofelone/naproxen clinical trial −0.81 (−1.70, 0.08)
NFOAS −1.09 (−1.56, −0.61)
TASOAC−p180 −1.33 (−2.15, −0.51)
TASOAC−TMIC
MOST −1.62 (−1.92, −1.31)
Meta −1.41 (−1.67, −1.16)
Heterogeneity: I^2=28%; tau=0.03
PC ae C42:4
CODING −1.44 (−2.13, −0.74)
LEAP−OA −0.82 (−1.31, −0.32)
Licofelone/naproxen clinical trial −0.30 (−1.17, 0.57)
NFOAS −0.69 (−1.16, −0.21)
TASOAC−p180 −1.06 (−1.88, −0.23)
TASOAC−TMIC
MOST −1.07 (−1.38, −0.76)
Meta −0.93 (−1.17, −0.69)
Heterogeneity: I^2=19%; tau=0.02
PC ae C42:5
CODING −1.17 (−1.84, −0.50)
LEAP−OA −0.54 (−1.05, −0.04)
Licofelone/naproxen clinical trial −0.22 (−1.09, 0.65)
NFOAS −0.57 (−1.05, −0.09)
TASOAC−p180 −1.26 (−2.07, −0.44)
TASOAC−TMIC
MOST −0.93 (−1.24, −0.61)
Meta −0.79 (−1.04, −0.53)
Heterogeneity: I^2=25%; tau=0.02
PC ae C44:3
CODING −1.14 (−1.83, −0.45)
LEAP−OA −0.71 (−1.21, −0.21)
Licofelone/naproxen clinical trial 0.11 (−0.77, 0.98)
NFOAS −0.66 (−1.13, −0.18)
TASOAC−p180 −1.01 (−1.84, −0.18)
TASOAC−TMIC
MOST −0.84 (−1.15, −0.53)
Meta −0.76 (−0.99, −0.53)
Heterogeneity: I^2=13%; tau=0.01
PC ae C44:4
CODING −0.64 (−1.33, 0.05)
LEAP−OA −0.48 (−0.98, 0.02)
Licofelone/naproxen clinical trial −0.54 (−1.41, 0.33)
NFOAS −0.55 (−1.03, −0.08)
TASOAC−p180 −1.49 (−2.29, −0.68)
TASOAC−TMIC
MOST −1.01 (−1.32, −0.70)
Meta −0.78 (−1.06, −0.50)
Heterogeneity: I^2=35%; tau=0.04
PC ae C44:5
CODING −0.98 (−1.65, −0.31)
LEAP−OA −0.40 (−0.90, 0.10)
Licofelone/naproxen clinical trial −1.06 (−1.92, −0.21)
NFOAS −0.23 (−0.71, 0.24)
TASOAC−p180 −1.07 (−1.88, −0.25)
TASOAC−TMIC
MOST −1.10 (−1.41, −0.79)
Meta −0.77 (−1.13, −0.42)
Heterogeneity: I^2=60%; tau=0.11
−2.5 −2 −1.5 −1 −0.5 0 0.5 1
Beta coefficient for BMI (95% CI)
per 1−SD increment in metabolite concentration

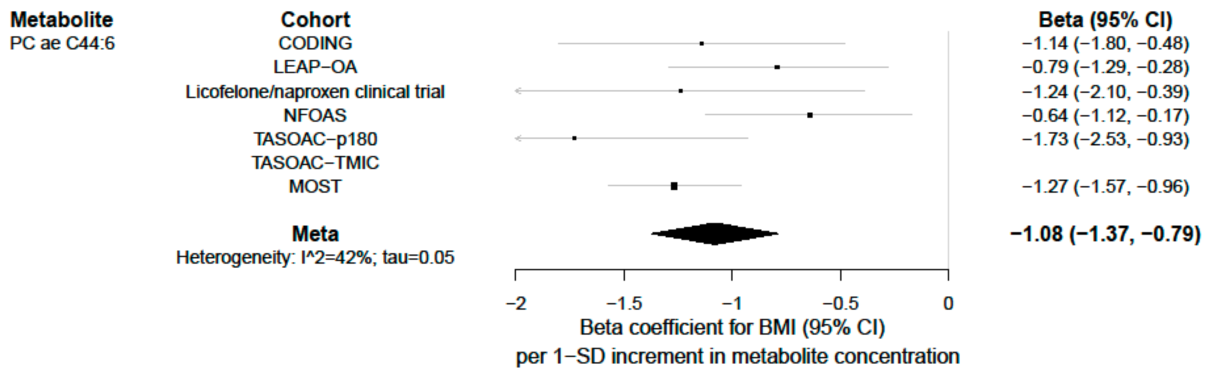

CODING: Complex Diseases in the Newfoundland population: Environment and Genetics; LEAP-OA: Longitudinal Evaluation in the Arthritis Program, Osteoarthritis Study; MOST: Multicenter Osteoarthritis Study; NFOAS: Newfoundland Osteoarthritis Study; TASOAC-p180: Tasmanian Older Adult Cohort profiled with Biocrates AbsoluteIDQ® p180 kit; TASOAC-TMIC: Tasmanian Older Adult Cohort profiled with the TMIC Prime Metabolomics Profiling Assay; a: acyl; aa: acyl-acyl; ae: acyl-alkyl; C0: carnitine; C3: propionylcarnitine; Cx:y: where x is the number of carbons in the fatty acid side chain; y is the number of double bonds in the fatty acid side chain; PC: phophatidylcholine. BMI: body mass index; CI: confidence interval; SD: standard error.

Beta coefficient and CI for individual cohorts were obtained by multivariable linear regression adjusting for age, sex, and osteoarthritis status. Those for meta-analysis were obtained by random effects meta-analysis with inverse variance as weights on the summary statistics from each cohort.

Figure S2. Forest plots for metabolites associated with obesity

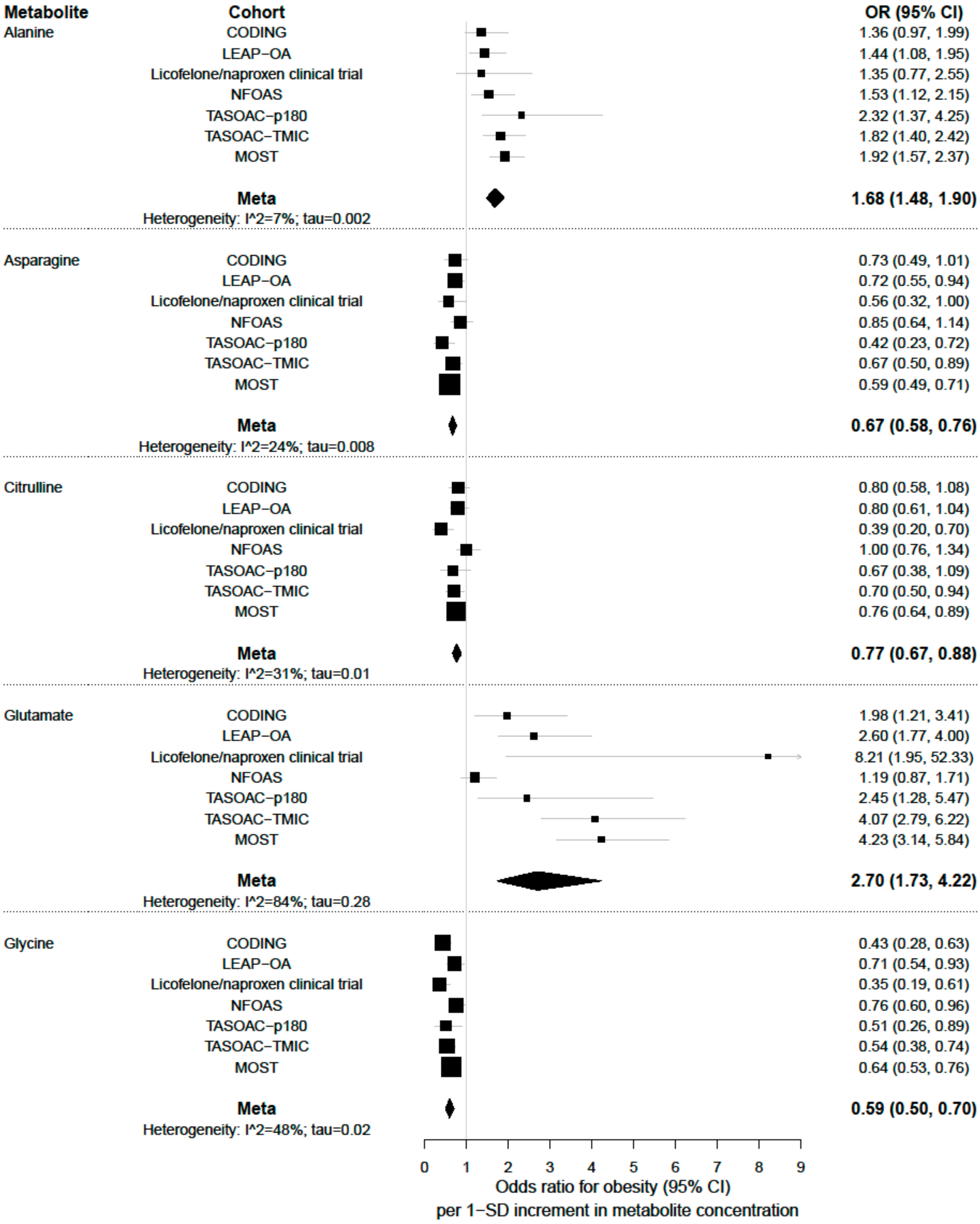

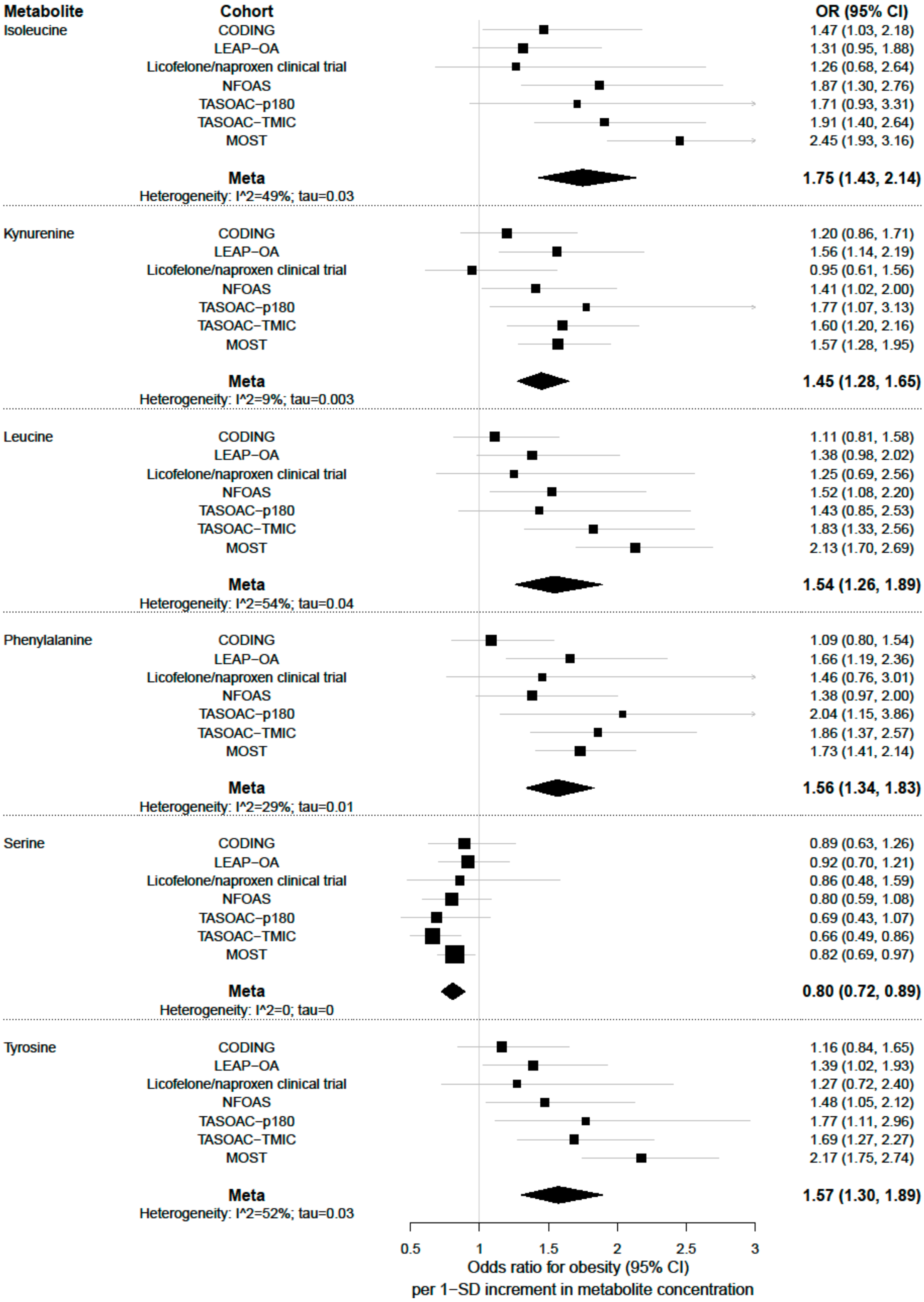
Metabolite
Cohort
OR (95% CI)
Isoleucine
CODING 1.47 (1.03, 2.18)
LEAP−OA 1.31 (0.95, 1.88)
Licofelone/naproxen clinical trial 1.26 (0.68, 2.64)
NFOAS 1.87 (1.30, 2.76)
TASOAC−p180 1.71 (0.93, 3.31)
TASOAC−TMIC 1.91 (1.40, 2.64)
MOST 2.45 (1.93, 3.16)
Meta 1.75 (1.43, 2.14)
Heterogeneity: I^2=49%; tau=0.03
Kynurenine
CODING 1.20 (0.86, 1.71)
LEAP−OA 1.56 (1.14, 2.19)
Licofelone/naproxen clinical trial 0.95 (0.61, 1.56)
NFOAS 1.41 (1.02, 2.00)
TASOAC−p180 1.77 (1.07, 3.13)
TASOAC−TMIC 1.60 (1.20, 2.16)
MOST 1.57 (1.28, 1.95)
Meta 1.45 (1.28, 1.65)
Heterogeneity: I^2=9%; tau=0.003
Leucine
CODING 1.11 (0.81, 1.58)
LEAP−OA 1.38 (0.98, 2.02)
Licofelone/naproxen clinical trial 1.25 (0.69, 2.56)
NFOAS 1.52 (1.08, 2.20)
TASOAC−p180 1.43 (0.85, 2.53)
TASOAC−TMIC 1.83 (1.33, 2.56)
MOST 2.13 (1.70, 2.69)
Meta 1.54 (1.26, 1.89)
Heterogeneity: I^2=54%; tau=0.04
Phenylalanine
CODING 1.09 (0.80, 1.54)
LEAP−OA 1.66 (1.19, 2.36)
Licofelone/naproxen clinical trial 1.46 (0.76, 3.01)
NFOAS 1.38 (0.97, 2.00)
TASOAC−p180 2.04 (1.15, 3.86)
TASOAC−TMIC 1.86 (1.37, 2.57)
MOST 1.73 (1.41, 2.14)
Meta 1.56 (1.34, 1.83)
Heterogeneity: I^2=29%; tau=0.01
Serine
CODING 0.89 (0.63, 1.26)
LEAP−OA 0.92 (0.70, 1.21)
Licofelone/naproxen clinical trial 0.86 (0.48, 1.59)
NFOAS 0.80 (0.59, 1.08)
TASOAC−p180 0.69 (0.43, 1.07)
TASOAC−TMIC 0.66 (0.49, 0.86)
MOST 0.82 (0.69, 0.97)
Meta 0.80 (0.72, 0.89)
Heterogeneity: I^2=0; tau=0
Tyrosine
CODING 1.16 (0.84, 1.65)
LEAP−OA 1.39 (1.02, 1.93)
Licofelone/naproxen clinical trial 1.27 (0.72, 2.40)
NFOAS 1.48 (1.05, 2.12)
TASOAC−p180 1.77 (1.11, 2.96)
TASOAC−TMIC 1.69 (1.27, 2.27)
MOST 2.17 (1.75, 2.74)
Meta 1.57 (1.30, 1.89)
Heterogeneity: I^2=52%; tau=0.03
0.5
1
1.5
2
2.5
3
Odds ratio for obesity (95% CI)
per 1−SD increment in metabolite concentration

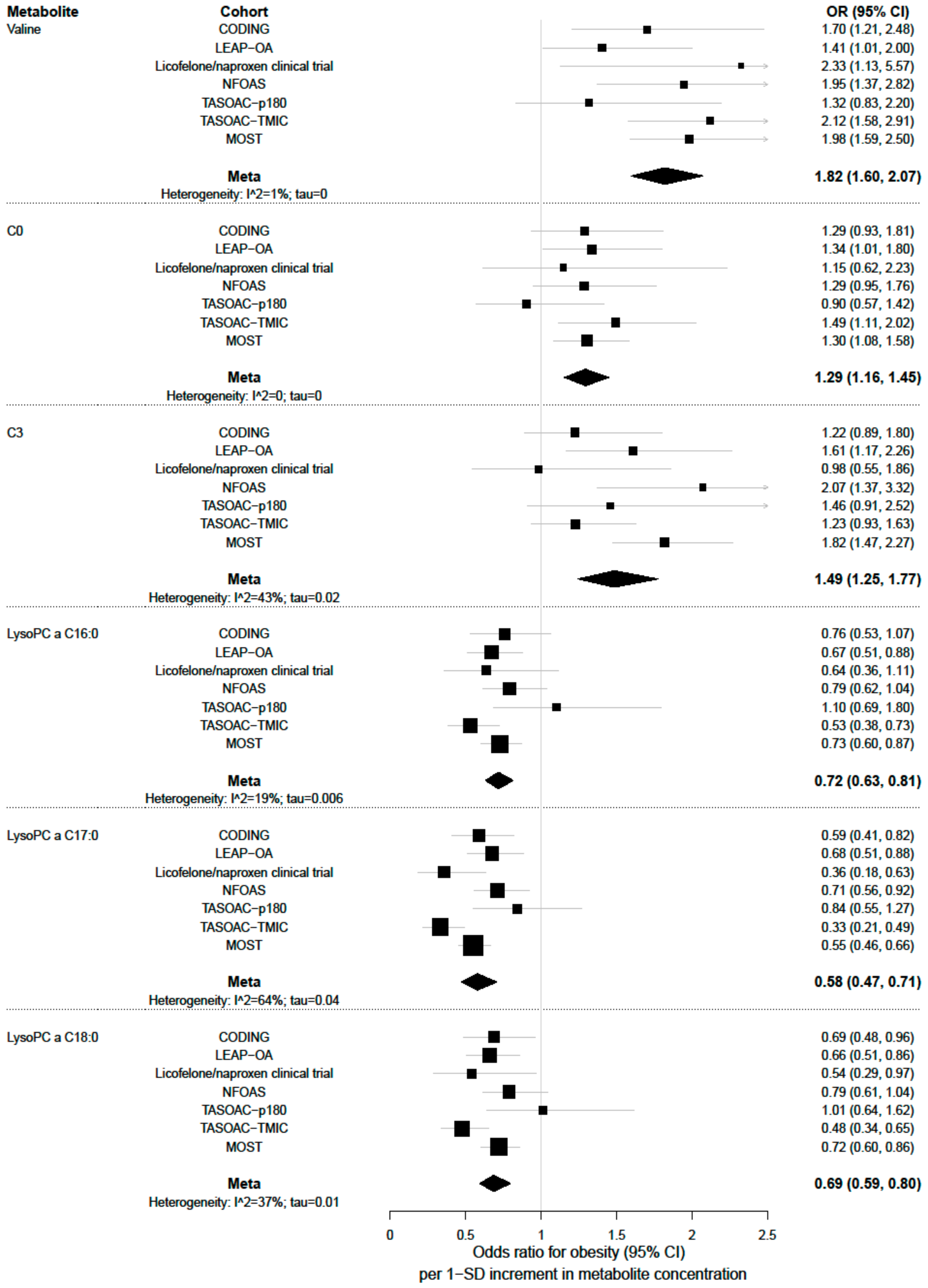
Metabolite
Cohort
OR (95% CI)
Valine
CODING
1.70 (1.21, 2.48)
LEAP−OA
1.41 (1.01, 2.00)
Licofelone/naproxen clinical trial
2.33 (1.13, 5.57)
NFOAS
1.95 (1.37, 2.82)
TASOAC−p180
1.32 (0.83, 2.20)
TASOAC−TMIC
2.12 (1.58, 2.91)
MOST
1.98 (1.59, 2.50)
Meta
1.82 (1.60, 2.07)
Heterogeneity: I^2=1%; tau=0
C0
CODING
1.29 (0.93, 1.81)
LEAP−OA
1.34 (1.01, 1.80)
Licofelone/naproxen clinical trial
1.15 (0.62, 2.23)
NFOAS
1.29 (0.95, 1.76)
TASOAC−p180
0.90 (0.57, 1.42)
TASOAC−TMIC
1.49 (1.11, 2.02)
MOST
1.30 (1.08, 1.58)
Meta
1.29 (1.16, 1.45)
Heterogeneity: I^2=0; tau=0
C3
CODING
1.22 (0.89, 1.80)
LEAP−OA
1.61 (1.17, 2.26)
Licofelone/naproxen clinical trial
0.98 (0.55, 1.86)
NFOAS
2.07 (1.37, 3.32)
TASOAC−p180
1.46 (0.91, 2.52)
TASOAC−TMIC
1.23 (0.93, 1.63)
MOST
1.82 (1.47, 2.27)
Meta
1.49 (1.25, 1.77)
Heterogeneity: I^2=43%; tau=0.02
LysoPC a C16:0
CODING
0.76 (0.53, 1.07)
LEAP−OA
0.67 (0.51, 0.88)
Licofelone/naproxen clinical trial
0.64 (0.36, 1.11)
NFOAS
0.79 (0.62, 1.04)
TASOAC−p180
1.10 (0.69, 1.80)
TASOAC−TMIC
0.53 (0.38, 0.73)
MOST
0.73 (0.60, 0.87)
Meta
0.72 (0.63, 0.81)
Heterogeneity: I^2=19%; tau=0.006
LysoPC a C17:0
CODING
0.59 (0.41, 0.82)
LEAP−OA
0.68 (0.51, 0.88)
Licofelone/naproxen clinical trial
0.36 (0.18, 0.63)
NFOAS
0.71 (0.56, 0.92)
TASOAC−p180
0.84 (0.55, 1.27)
TASOAC−TMIC
0.33 (0.21, 0.49)
MOST
0.55 (0.46, 0.66)
Meta
0.58 (0.47, 0.71)
Heterogeneity: I^2=64%; tau=0.04
LysoPC a C18:0
CODING
0.69 (0.48, 0.96)
LEAP−OA
0.66 (0.51, 0.86)
Licofelone/naproxen clinical trial
0.54 (0.29, 0.97)
NFOAS
0.79 (0.61, 1.04)
TASOAC−p180
1.01 (0.64, 1.62)
TASOAC−TMIC
0.48 (0.34, 0.65)
MOST
0.72 (0.60, 0.86)
Meta
0.69 (0.59, 0.80)
Heterogeneity: I^2=37%; tau=0.01
0
0.5
1
1.5
2
2.5
Odds ratio for obesity (95% CI)
per 1−SD increment in metabolite concentration

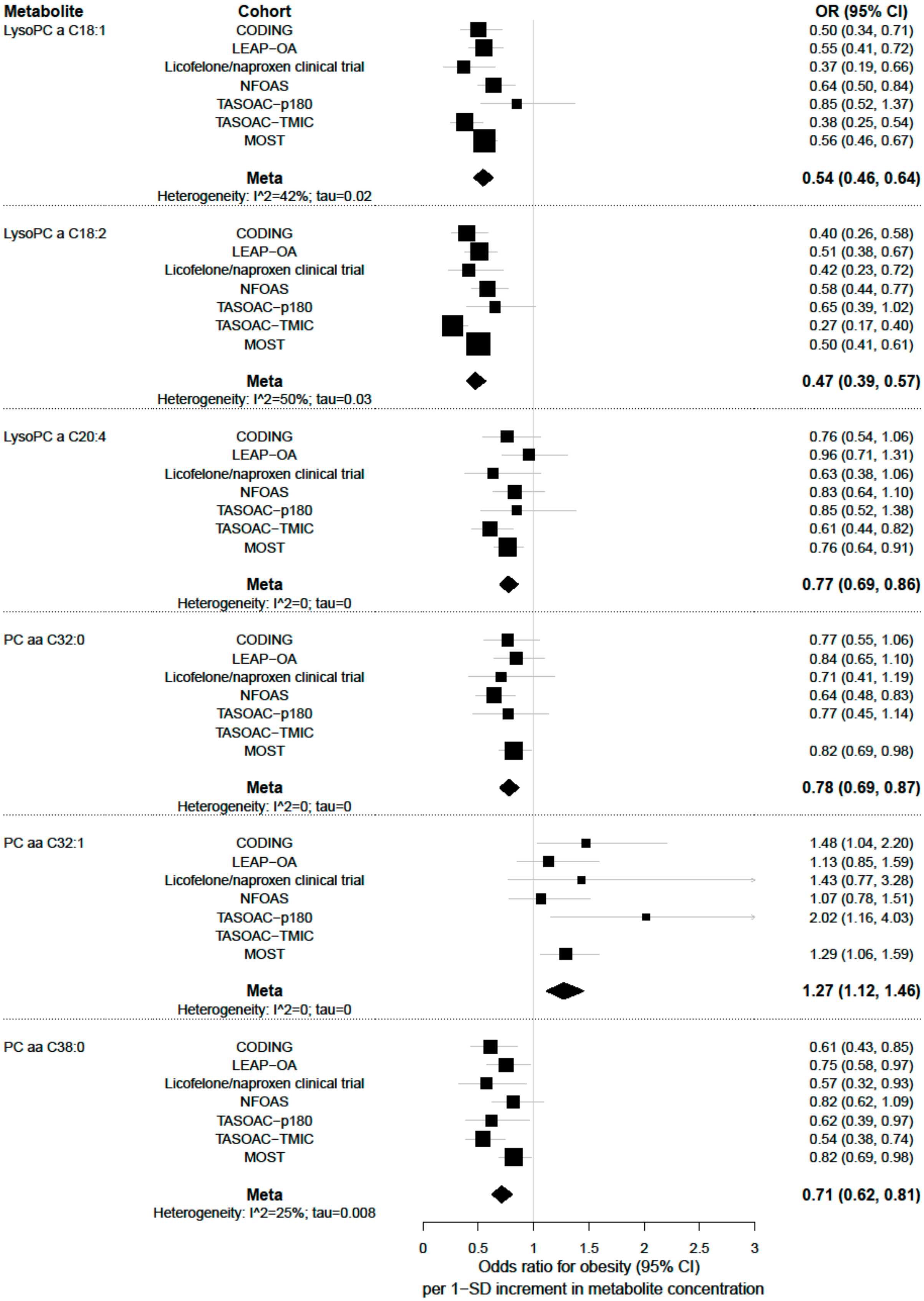
Metabolite
Cohort
OR (95% CI)
LysoPC a C18:1
CODING 0.50 (0.34, 0.71)
LEAP−OA 0.55 (0.41, 0.72)
Licofelone/naproxen clinical trial 0.37 (0.19, 0.66)
NFOAS 0.64 (0.50, 0.84)
TASOAC−p180 0.85 (0.52, 1.37)
TASOAC−TMIC 0.38 (0.25, 0.54)
MOST 0.56 (0.46, 0.67)
Meta 0.54 (0.46, 0.64)
Heterogeneity: I^2=42%; tau=0.02
LysoPC a C18:2
CODING 0.40 (0.26, 0.58)
LEAP−OA 0.51 (0.38, 0.67)
Licofelone/naproxen clinical trial 0.42 (0.23, 0.72)
NFOAS 0.58 (0.44, 0.77)
TASOAC−p180 0.65 (0.39, 1.02)
TASOAC−TMIC 0.27 (0.17, 0.40)
MOST 0.50 (0.41, 0.61)
Meta 0.47 (0.39, 0.57)
Heterogeneity: I^2=50%; tau=0.03
LysoPC a C20:4
CODING 0.76 (0.54, 1.06)
LEAP−OA 0.96 (0.71, 1.31)
Licofelone/naproxen clinical trial 0.63 (0.38, 1.06)
NFOAS 0.83 (0.64, 1.10)
TASOAC−p180 0.85 (0.52, 1.38)
TASOAC−TMIC 0.61 (0.44, 0.82)
MOST 0.76 (0.64, 0.91)
Meta 0.77 (0.69, 0.86)
Heterogeneity: I^2=0; tau=0
PC aa C32:0
CODING 0.77 (0.55, 1.06)
LEAP−OA 0.84 (0.65, 1.10)
Licofelone/naproxen clinical trial 0.71 (0.41, 1.19)
NFOAS 0.64 (0.48, 0.83)
TASOAC−p180 0.77 (0.45, 1.14)
TASOAC−TMIC
MOST 0.82 (0.69, 0.98)
Meta 0.78 (0.69, 0.87)
Heterogeneity: I^2=0; tau=0
PC aa C32:1
CODING 1.48 (1.04, 2.20)
LEAP−OA 1.13 (0.85, 1.59)
Licofelone/naproxen clinical trial 1.43 (0.77, 3.28)
NFOAS 1.07 (0.78, 1.51)
TASOAC−p180 2.02 (1.16, 4.03)
TASOAC−TMIC
MOST 1.29 (1.06, 1.59)
Meta 1.27 (1.12, 1.46)
Heterogeneity: I^2=0; tau=0
PC aa C38:0
CODING 0.61 (0.43, 0.85)
LEAP−OA 0.75 (0.58, 0.97)
Licofelone/naproxen clinical trial 0.57 (0.32, 0.93)
NFOAS 0.82 (0.62, 1.09)
TASOAC−p180 0.62 (0.39, 0.97)
TASOAC−TMIC 0.54 (0.38, 0.74)
MOST 0.82 (0.69, 0.98)
Meta 0.71 (0.62, 0.81)
Heterogeneity: I^2=25%; tau=0.008
0 0.5 1 1.5 2 2.5 3
Odds ratio for obesity (95% CI)
per 1−SD increment in metabolite concentration

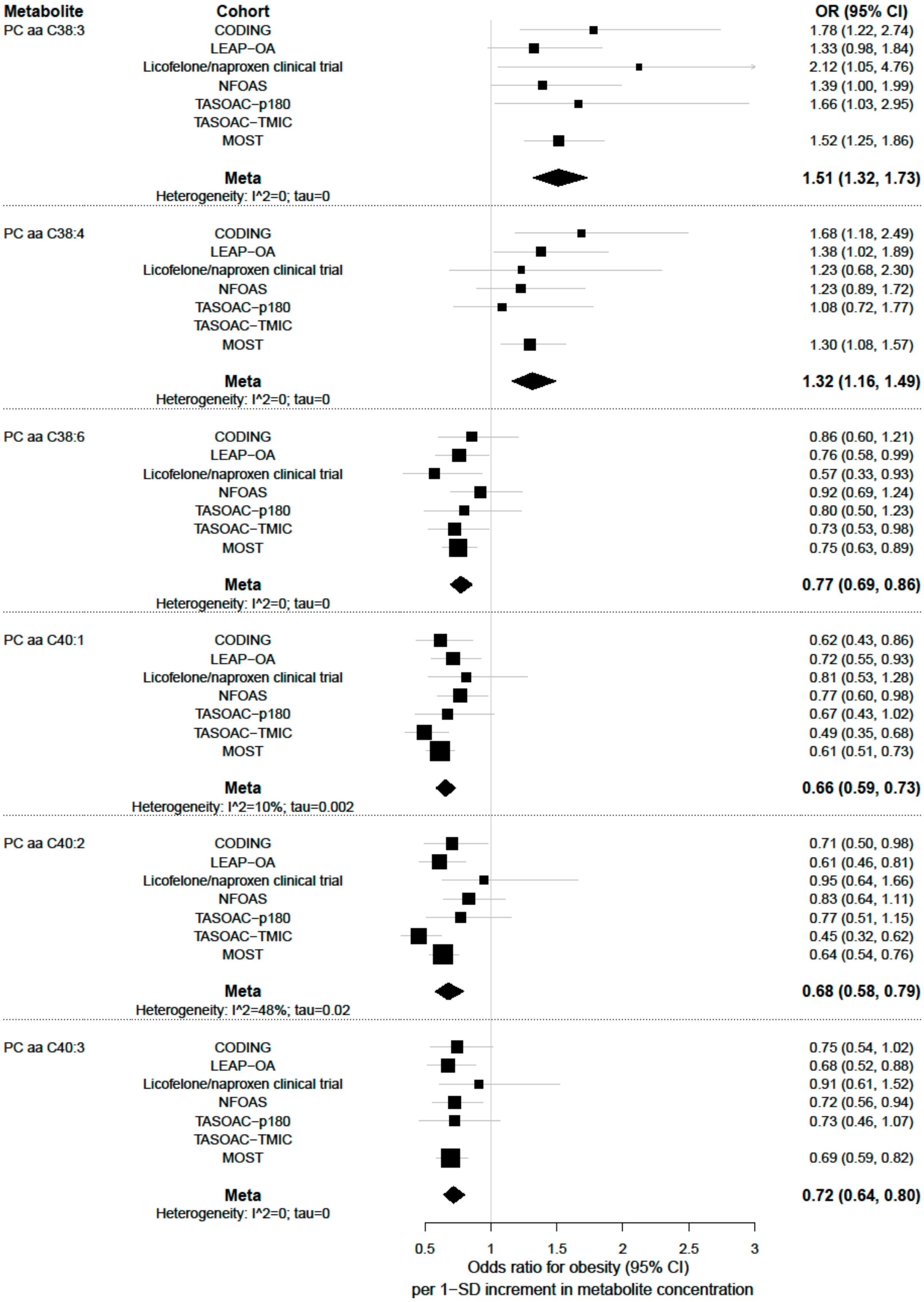
Metabolite
Cohort
OR (95% CI)
PC aa C38:3
CODING 1.78 (1.22, 2.74)
LEAP−OA 1.33 (0.98, 1.84)
Licofelone/naproxen clinical trial 2.12 (1.05, 4.76)
NFOAS 1.39 (1.00, 1.99)
TASOAC−p180 1.66 (1.03, 2.95)
TASOAC−TMIC
MOST 1.52 (1.25, 1.86)
Meta 1.51 (1.32, 1.73)
Heterogeneity: I^2=0; tau=0
PC aa C38:4
CODING 1.68 (1.18, 2.49)
LEAP−OA 1.38 (1.02, 1.89)
Licofelone/naproxen clinical trial 1.23 (0.68, 2.30)
NFOAS 1.23 (0.89, 1.72)
TASOAC−p180 1.08 (0.72, 1.77)
TASOAC−TMIC
MOST 1.30 (1.08, 1.57)
Meta 1.32 (1.16, 1.49)
Heterogeneity: I^2=0; tau=0
PC aa C38:6
CODING 0.86 (0.60, 1.21)
LEAP−OA 0.76 (0.58, 0.99)
Licofelone/naproxen clinical trial 0.57 (0.33, 0.93)
NFOAS 0.92 (0.69, 1.24)
TASOAC−p180 0.80 (0.50, 1.23)
TASOAC−TMIC 0.73 (0.53, 0.98)
MOST 0.75 (0.63, 0.89)
Meta 0.77 (0.69, 0.86)
Heterogeneity: I^2=0; tau=0
PC aa C40:1
CODING 0.62 (0.43, 0.86)
LEAP−OA 0.72 (0.55, 0.93)
Licofelone/naproxen clinical trial 0.81 (0.53, 1.28)
NFOAS 0.77 (0.60, 0.98)
TASOAC−p180 0.67 (0.43, 1.02)
TASOAC−TMIC 0.49 (0.35, 0.68)
MOST 0.61 (0.51, 0.73)
Meta 0.66 (0.59, 0.73)
Heterogeneity: I^2=10%; tau=0.002
PC aa C40:2
CODING 0.71 (0.50, 0.98)
LEAP−OA 0.61 (0.46, 0.81)
Licofelone/naproxen clinical trial 0.95 (0.64, 1.66)
NFOAS 0.83 (0.64, 1.11)
TASOAC−p180 0.77 (0.51, 1.15)
TASOAC−TMIC 0.45 (0.32, 0.62)
MOST 0.64 (0.54, 0.76)
Meta 0.68 (0.58, 0.79)
Heterogeneity: I^2=48%; tau=0.02
PC aa C40:3
CODING 0.75 (0.54, 1.02)
LEAP−OA 0.68 (0.52, 0.88)
Licofelone/naproxen clinical trial 0.91 (0.61, 1.52)
NFOAS 0.72 (0.56, 0.94)
TASOAC−p180 0.73 (0.46, 1.07)
TASOAC−TMIC
MOST 0.69 (0.59, 0.82)
Meta 0.72 (0.64, 0.80)
Heterogeneity: I^2=0; tau=0
0.5
1
1.5
2
2.5
3
Odds ratio for obesity (95% CI)
per 1−SD increment in metabolite concentration

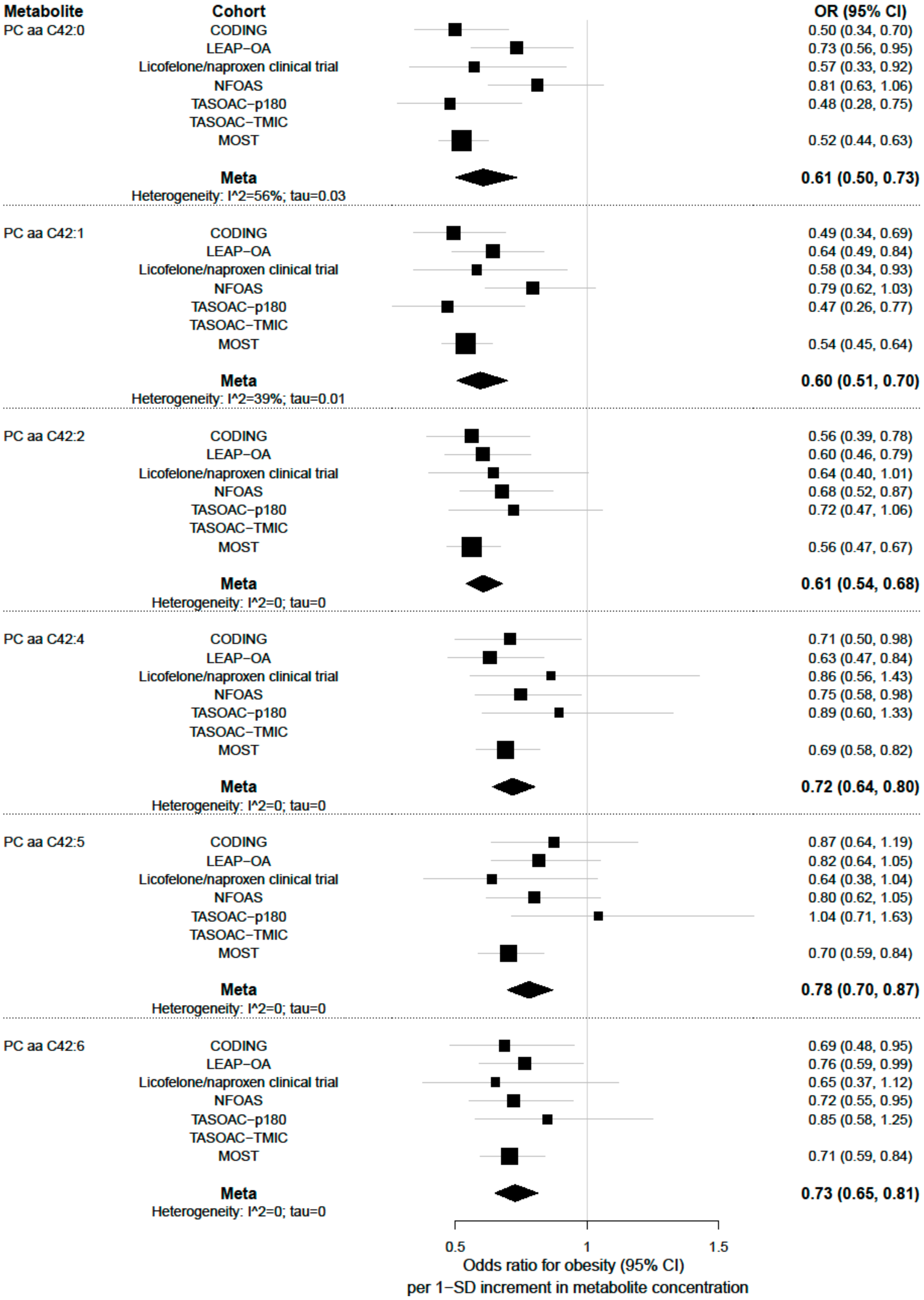
Metabolite
Cohort
OR (95% CI)
PC aa C42:0
CODING 0.50 (0.34, 0.70)
LEAP−OA 0.73 (0.56, 0.95)
Licofelone/naproxen clinical trial 0.57 (0.33, 0.92)
NFOAS 0.81 (0.63, 1.06)
TASOAC−p180 0.48 (0.28, 0.75)
TASOAC−TMIC
MOST 0.52 (0.44, 0.63)
Meta 0.61 (0.50, 0.73)
Heterogeneity: I^2=56%; tau=0.03
PC aa C42:1
CODING 0.49 (0.34, 0.69)
LEAP−OA 0.64 (0.49, 0.84)
Licofelone/naproxen clinical trial 0.58 (0.34, 0.93)
NFOAS 0.79 (0.62, 1.03)
TASOAC−p180 0.47 (0.26, 0.77)
TASOAC−TMIC
MOST 0.54 (0.45, 0.64)
Meta 0.60 (0.51, 0.70)
Heterogeneity: I^2=39%; tau=0.01
PC aa C42:2
CODING 0.56 (0.39, 0.78)
LEAP−OA 0.60 (0.46, 0.79)
Licofelone/naproxen clinical trial 0.64 (0.40, 1.01)
NFOAS 0.68 (0.52, 0.87)
TASOAC−p180 0.72 (0.47, 1.06)
TASOAC−TMIC
MOST 0.56 (0.47, 0.67)
Meta 0.61 (0.54, 0.68)
Heterogeneity: I^2=0; tau=0
PC aa C42:4
CODING 0.71 (0.50, 0.98)
LEAP−OA 0.63 (0.47, 0.84)
Licofelone/naproxen clinical trial 0.86 (0.56, 1.43)
NFOAS 0.75 (0.58, 0.98)
TASOAC−p180 0.89 (0.60, 1.33)
TASOAC−TMIC
MOST 0.69 (0.58, 0.82)
Meta 0.72 (0.64, 0.80)
Heterogeneity: I^2=0; tau=0
PC aa C42:5
CODING 0.87 (0.64, 1.19)
LEAP−OA 0.82 (0.64, 1.05)
Licofelone/naproxen clinical trial 0.64 (0.38, 1.04)
NFOAS 0.80 (0.62, 1.05)
TASOAC−p180 1.04 (0.71, 1.63)
TASOAC−TMIC
MOST 0.70 (0.59, 0.84)
Meta 0.78 (0.70, 0.87)
Heterogeneity: I^2=0; tau=0
PC aa C42:6
CODING 0.69 (0.48, 0.95)
LEAP−OA 0.76 (0.59, 0.99)
Licofelone/naproxen clinical trial 0.65 (0.37, 1.12)
NFOAS 0.72 (0.55, 0.95)
TASOAC−p180 0.85 (0.58, 1.25)
TASOAC−TMIC
MOST 0.71 (0.59, 0.84)
Meta 0.73 (0.65, 0.81)
Heterogeneity: I^2=0; tau=0
0.5
1
1.5
Odds ratio for obesity (95% CI)
per 1−SD increment in metabolite concentration

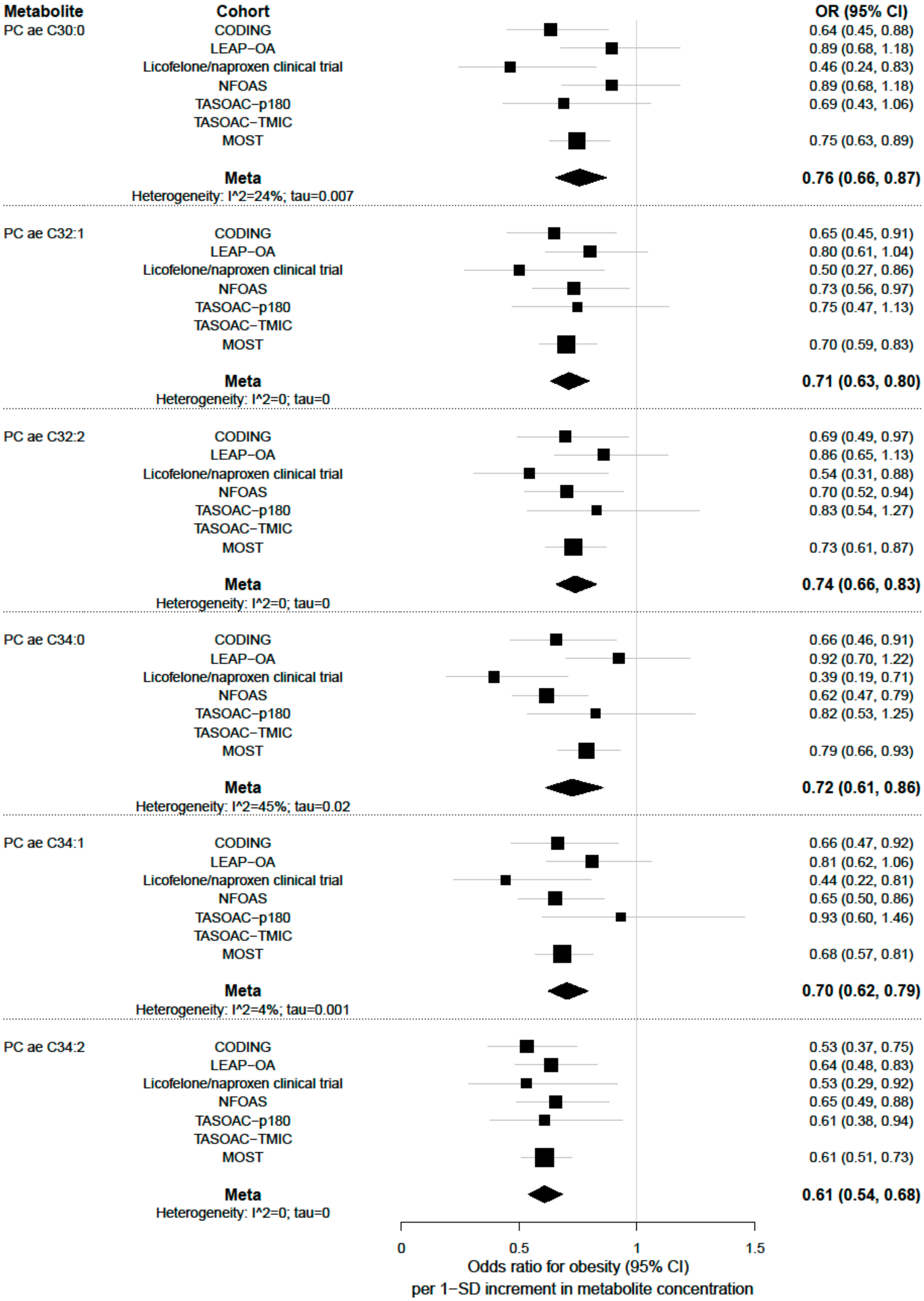
Metabolite
Cohort
OR (95% CI)
PC ae C30:0
CODING 0.64 (0.45, 0.88)
LEAP−OA 0.89 (0.68, 1.18)
Licofelone/naproxen clinical trial 0.46 (0.24, 0.83)
NFOAS 0.89 (0.68, 1.18)
TASOAC−p180 0.69 (0.43, 1.06)
TASOAC−TMIC
MOST 0.75 (0.63, 0.89)
Meta 0.76 (0.66, 0.87)
Heterogeneity: I^2=24%; tau=0.007
PC ae C32:1
CODING 0.65 (0.45, 0.91)
LEAP−OA 0.80 (0.61, 1.04)
Licofelone/naproxen clinical trial 0.50 (0.27, 0.86)
NFOAS 0.73 (0.56, 0.97)
TASOAC−p180 0.75 (0.47, 1.13)
TASOAC−TMIC
MOST 0.70 (0.59, 0.83)
Meta 0.71 (0.63, 0.80)
Heterogeneity: I^2=0; tau=0
PC ae C32:2
CODING 0.69 (0.49, 0.97)
LEAP−OA 0.86 (0.65, 1.13)
Licofelone/naproxen clinical trial 0.54 (0.31, 0.88)
NFOAS 0.70 (0.52, 0.94)
TASOAC−p180 0.83 (0.54, 1.27)
TASOAC−TMIC
MOST 0.73 (0.61, 0.87)
Meta 0.74 (0.66, 0.83)
Heterogeneity: I^2=0; tau=0
PC ae C34:0
CODING 0.66 (0.46, 0.91)
LEAP−OA 0.92 (0.70, 1.22)
Licofelone/naproxen clinical trial 0.39 (0.19, 0.71)
NFOAS 0.62 (0.47, 0.79)
TASOAC−p180 0.82 (0.53, 1.25)
TASOAC−TMIC
MOST 0.79 (0.66, 0.93)
Meta 0.72 (0.61, 0.86)
Heterogeneity: I^2=45%; tau=0.02
PC ae C34:1
CODING 0.66 (0.47, 0.92)
LEAP−OA 0.81 (0.62, 1.06)
Licofelone/naproxen clinical trial 0.44 (0.22, 0.81)
NFOAS 0.65 (0.50, 0.86)
TASOAC−p180 0.93 (0.60, 1.46)
TASOAC−TMIC
MOST 0.68 (0.57, 0.81)
Meta 0.70 (0.62, 0.79)
Heterogeneity: I^2=4%; tau=0.001
PC ae C34:2
CODING 0.53 (0.37, 0.75)
LEAP−OA 0.64 (0.48, 0.83)
Licofelone/naproxen clinical trial 0.53 (0.29, 0.92)
NFOAS 0.65 (0.49, 0.88)
TASOAC−p180 0.61 (0.38, 0.94)
TASOAC−TMIC
MOST 0.61 (0.51, 0.73)
Meta 0.61 (0.54, 0.68)
Heterogeneity: I^2=0; tau=0
0
0.5
1
1.5
Odds ratio for obesity (95% CI)
per 1−SD increment in metabolite concentration

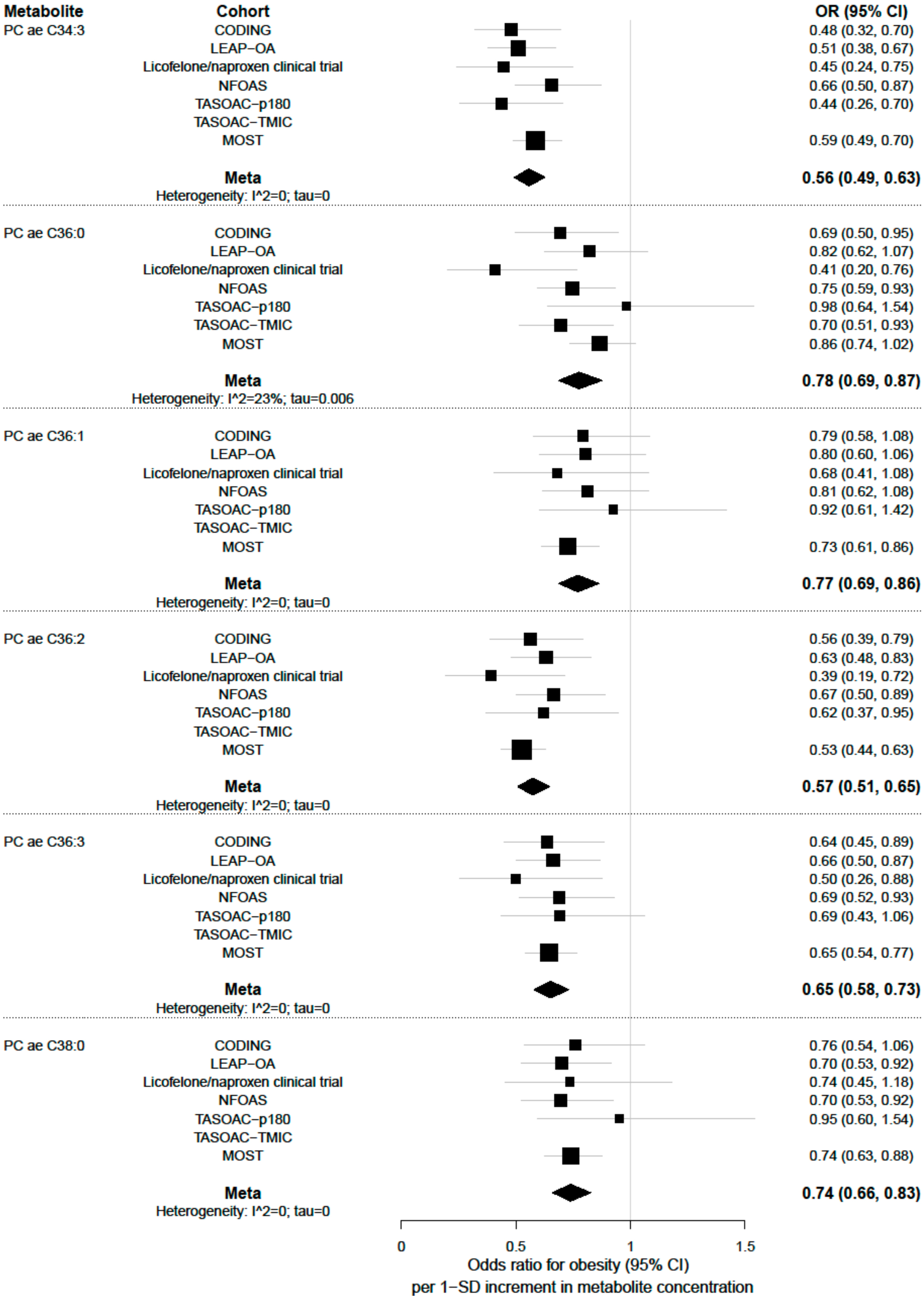
Metabolite
Cohort
OR (95% CI)
PC ae C34:3
CODING 0.48 (0.32, 0.70)
LEAP−OA 0.51 (0.38, 0.67)
Licofelone/naproxen clinical trial 0.45 (0.24, 0.75)
NFOAS 0.66 (0.50, 0.87)
TASOAC−p180 0.44 (0.26, 0.70)
TASOAC−TMIC
MOST 0.59 (0.49, 0.70)
Meta 0.56 (0.49, 0.63)
Heterogeneity: I^2=0; tau=0
PC ae C36:0
CODING 0.69 (0.50, 0.95)
LEAP−OA 0.82 (0.62, 1.07)
Licofelone/naproxen clinical trial 0.41 (0.20, 0.76)
NFOAS 0.75 (0.59, 0.93)
TASOAC−p180 0.98 (0.64, 1.54)
TASOAC−TMIC 0.70 (0.51, 0.93)
MOST 0.86 (0.74, 1.02)
Meta 0.78 (0.69, 0.87)
Heterogeneity: I^2=23%; tau=0.006
PC ae C36:1
CODING 0.79 (0.58, 1.08)
LEAP−OA 0.80 (0.60, 1.06)
Licofelone/naproxen clinical trial 0.68 (0.41, 1.08)
NFOAS 0.81 (0.62, 1.08)
TASOAC−p180 0.92 (0.61, 1.42)
TASOAC−TMIC
MOST 0.73 (0.61, 0.86)
Meta 0.77 (0.69, 0.86)
Heterogeneity: I^2=0; tau=0
PC ae C36:2
CODING 0.56 (0.39, 0.79)
LEAP−OA 0.63 (0.48, 0.83)
Licofelone/naproxen clinical trial 0.39 (0.19, 0.72)
NFOAS 0.67 (0.50, 0.89)
TASOAC−p180 0.62 (0.37, 0.95)
TASOAC−TMIC
MOST 0.53 (0.44, 0.63)
Meta 0.57 (0.51, 0.65)
Heterogeneity: I^2=0; tau=0
PC ae C36:3
CODING 0.64 (0.45, 0.89)
LEAP−OA 0.66 (0.50, 0.87)
Licofelone/naproxen clinical trial 0.50 (0.26, 0.88)
NFOAS 0.69 (0.52, 0.93)
TASOAC−p180 0.69 (0.43, 1.06)
TASOAC−TMIC
MOST 0.65 (0.54, 0.77)
Meta 0.65 (0.58, 0.73)
Heterogeneity: I^2=0; tau=0
PC ae C38:0
CODING 0.76 (0.54, 1.06)
LEAP−OA 0.70 (0.53, 0.92)
Licofelone/naproxen clinical trial 0.74 (0.45, 1.18)
NFOAS 0.70 (0.53, 0.92)
TASOAC−p180 0.95 (0.60, 1.54)
TASOAC−TMIC
MOST 0.74 (0.63, 0.88)
Meta 0.74 (0.66, 0.83)
Heterogeneity: I^2=0; tau=0
0
0.5
1
1.5
Odds ratio for obesity (95% CI)
per 1−SD increment in metabolite concentration

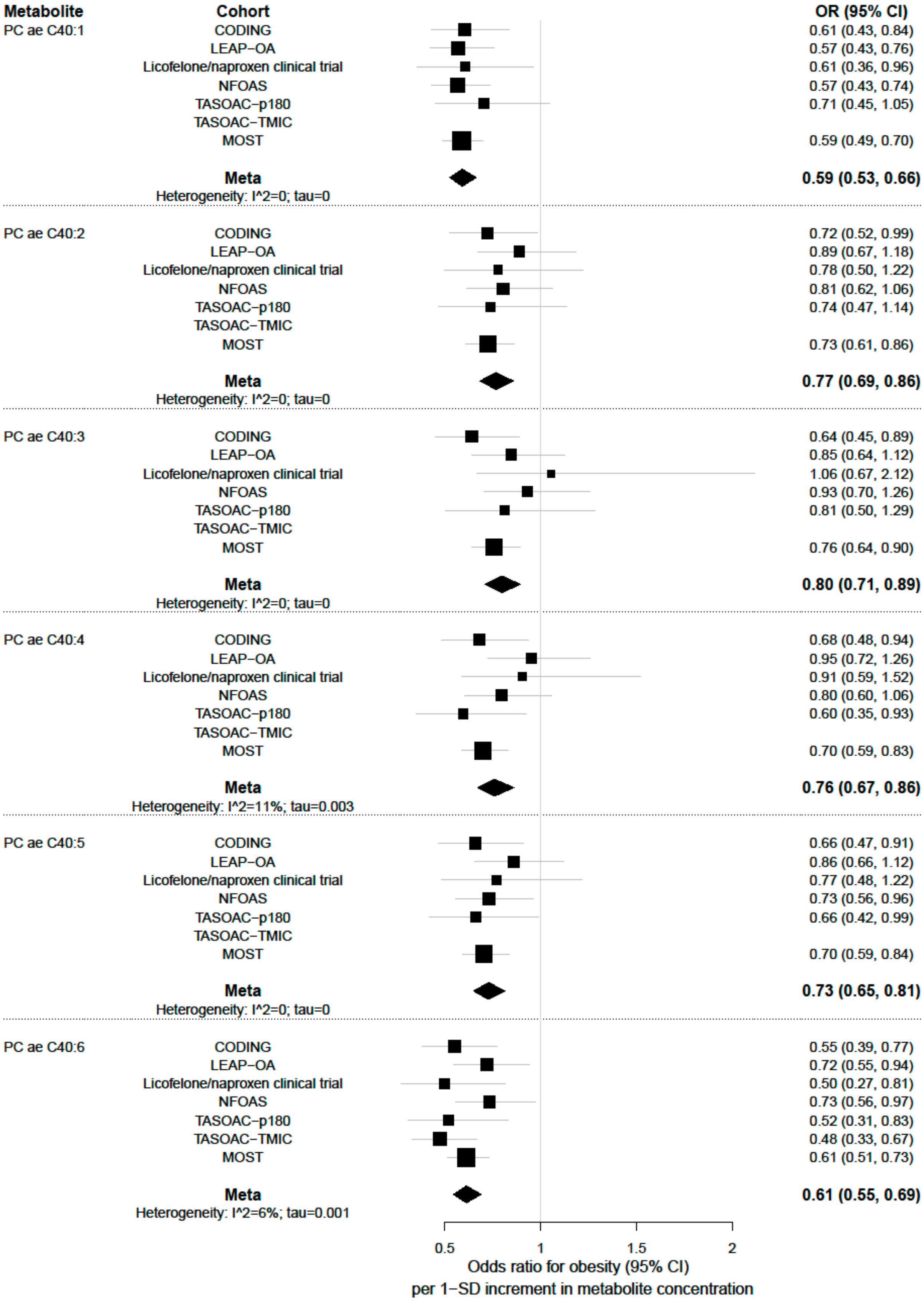
Metabolite
Cohort
OR (95% CI)
PC ae C40:1
CODING 0.61 (0.43, 0.84)
LEAP−OA 0.57 (0.43, 0.76)
Licofelone/naproxen clinical trial 0.61 (0.36, 0.96)
NFOAS 0.57 (0.43, 0.74)
TASOAC−p180 0.71 (0.45, 1.05)
TASOAC−TMIC
MOST 0.59 (0.49, 0.70)
Meta 0.59 (0.53, 0.66)
Heterogeneity: I^2=0; tau=0
PC ae C40:2
CODING 0.72 (0.52, 0.99)
LEAP−OA 0.89 (0.67, 1.18)
Licofelone/naproxen clinical trial 0.78 (0.50, 1.22)
NFOAS 0.81 (0.62, 1.06)
TASOAC−p180 0.74 (0.47, 1.14)
TASOAC−TMIC
MOST 0.73 (0.61, 0.86)
Meta 0.77 (0.69, 0.86)
Heterogeneity: I^2=0; tau=0
PC ae C40:3
CODING 0.64 (0.45, 0.89)
LEAP−OA 0.85 (0.64, 1.12)
Licofelone/naproxen clinical trial 1.06 (0.67, 2.12)
NFOAS 0.93 (0.70, 1.26)
TASOAC−p180 0.81 (0.50, 1.29)
TASOAC−TMIC
MOST 0.76 (0.64, 0.90)
Meta 0.80 (0.71, 0.89)
Heterogeneity: I^2=0; tau=0
PC ae C40:4
CODING 0.68 (0.48, 0.94)
LEAP−OA 0.95 (0.72, 1.26)
Licofelone/naproxen clinical trial 0.91 (0.59, 1.52)
NFOAS 0.80 (0.60, 1.06)
TASOAC−p180 0.60 (0.35, 0.93)
TASOAC−TMIC
MOST 0.70 (0.59, 0.83)
Meta 0.76 (0.67, 0.86)
Heterogeneity: I^2=11%; tau=0.003
PC ae C40:5
CODING 0.66 (0.47, 0.91)
LEAP−OA 0.86 (0.66, 1.12)
Licofelone/naproxen clinical trial 0.77 (0.48, 1.22)
NFOAS 0.73 (0.56, 0.96)
TASOAC−p180 0.66 (0.42, 0.99)
TASOAC−TMIC
MOST 0.70 (0.59, 0.84)
Meta 0.73 (0.65, 0.81)
Heterogeneity: I^2=0; tau=0
PC ae C40:6
CODING 0.55 (0.39, 0.77)
LEAP−OA 0.72 (0.55, 0.94)
Licofelone/naproxen clinical trial 0.50 (0.27, 0.81)
NFOAS 0.73 (0.56, 0.97)
TASOAC−p180 0.52 (0.31, 0.83)
TASOAC−TMIC 0.48 (0.33, 0.67)
MOST 0.61 (0.51, 0.73)
Meta 0.61 (0.55, 0.69)
Heterogeneity: I^2=6%; tau=0.001
0.5
1
1.5
2
Odds ratio for obesity (95% CI)
per 1−SD increment in metabolite concentration

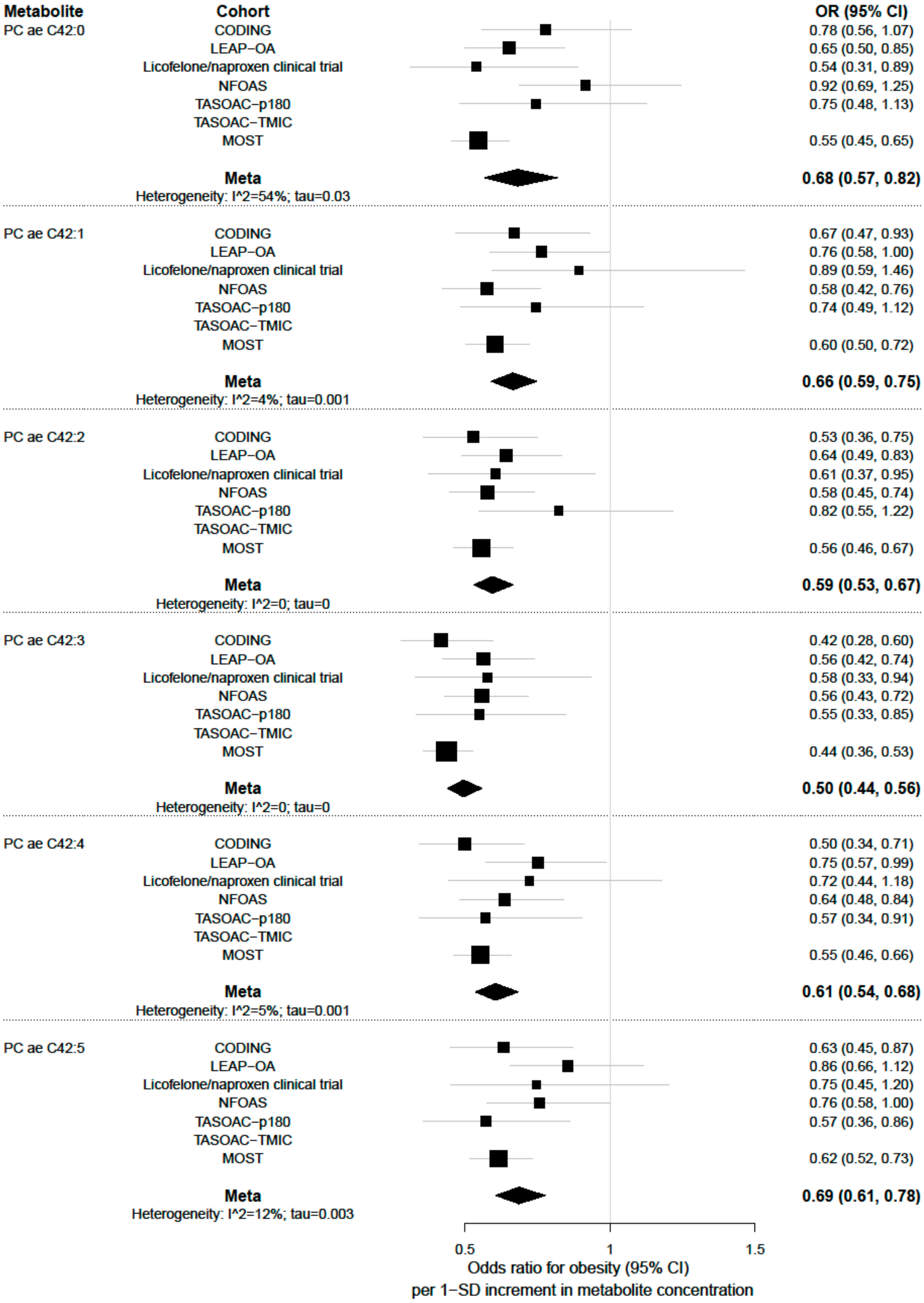
Metabolite
Cohort
OR (95% CI)
PC ae C42:0
CODING 0.78 (0.56, 1.07)
LEAP−OA 0.65 (0.50, 0.85)
Licofelone/naproxen clinical trial 0.54 (0.31, 0.89)
NFOAS 0.92 (0.69, 1.25)
TASOAC−p180 0.75 (0.48, 1.13)
TASOAC−TMIC
MOST 0.55 (0.45, 0.65)
Meta 0.68 (0.57, 0.82)
Heterogeneity: I^2=54%; tau=0.03
PC ae C42:1
CODING 0.67 (0.47, 0.93)
LEAP−OA 0.76 (0.58, 1.00)
Licofelone/naproxen clinical trial 0.89 (0.59, 1.46)
NFOAS 0.58 (0.42, 0.76)
TASOAC−p180 0.74 (0.49, 1.12)
TASOAC−TMIC
MOST 0.60 (0.50, 0.72)
Meta 0.66 (0.59, 0.75)
Heterogeneity: I^2=4%; tau=0.001
PC ae C42:2
CODING 0.53 (0.36, 0.75)
LEAP−OA 0.64 (0.49, 0.83)
Licofelone/naproxen clinical trial 0.61 (0.37, 0.95)
NFOAS 0.58 (0.45, 0.74)
TASOAC−p180 0.82 (0.55, 1.22)
TASOAC−TMIC
MOST 0.56 (0.46, 0.67)
Meta 0.59 (0.53, 0.67)
Heterogeneity: I^2=0; tau=0
PC ae C42:3
CODING 0.42 (0.28, 0.60)
LEAP−OA 0.56 (0.42, 0.74)
Licofelone/naproxen clinical trial 0.58 (0.33, 0.94)
NFOAS 0.56 (0.43, 0.72)
TASOAC−p180 0.55 (0.33, 0.85)
TASOAC−TMIC
MOST 0.44 (0.36, 0.53)
Meta 0.50 (0.44, 0.56)
Heterogeneity: I^2=0; tau=0
PC ae C42:4
CODING 0.50 (0.34, 0.71)
LEAP−OA 0.75 (0.57, 0.99)
Licofelone/naproxen clinical trial 0.72 (0.44, 1.18)
NFOAS 0.64 (0.48, 0.84)
TASOAC−p180 0.57 (0.34, 0.91)
TASOAC−TMIC
MOST 0.55 (0.46, 0.66)
Meta 0.61 (0.54, 0.68)
Heterogeneity: I^2=5%; tau=0.001
PC ae C42:5
CODING 0.63 (0.45, 0.87)
LEAP−OA 0.86 (0.66, 1.12)
Licofelone/naproxen clinical trial 0.75 (0.45, 1.20)
NFOAS 0.76 (0.58, 1.00)
TASOAC−p180 0.57 (0.36, 0.86)
TASOAC−TMIC
MOST 0.62 (0.52, 0.73)
Meta 0.69 (0.61, 0.78)
Heterogeneity: I^2=12%; tau=0.003
0.5
1
1.5
Odds ratio for obesity (95% CI)
per 1−SD increment in metabolite concentration

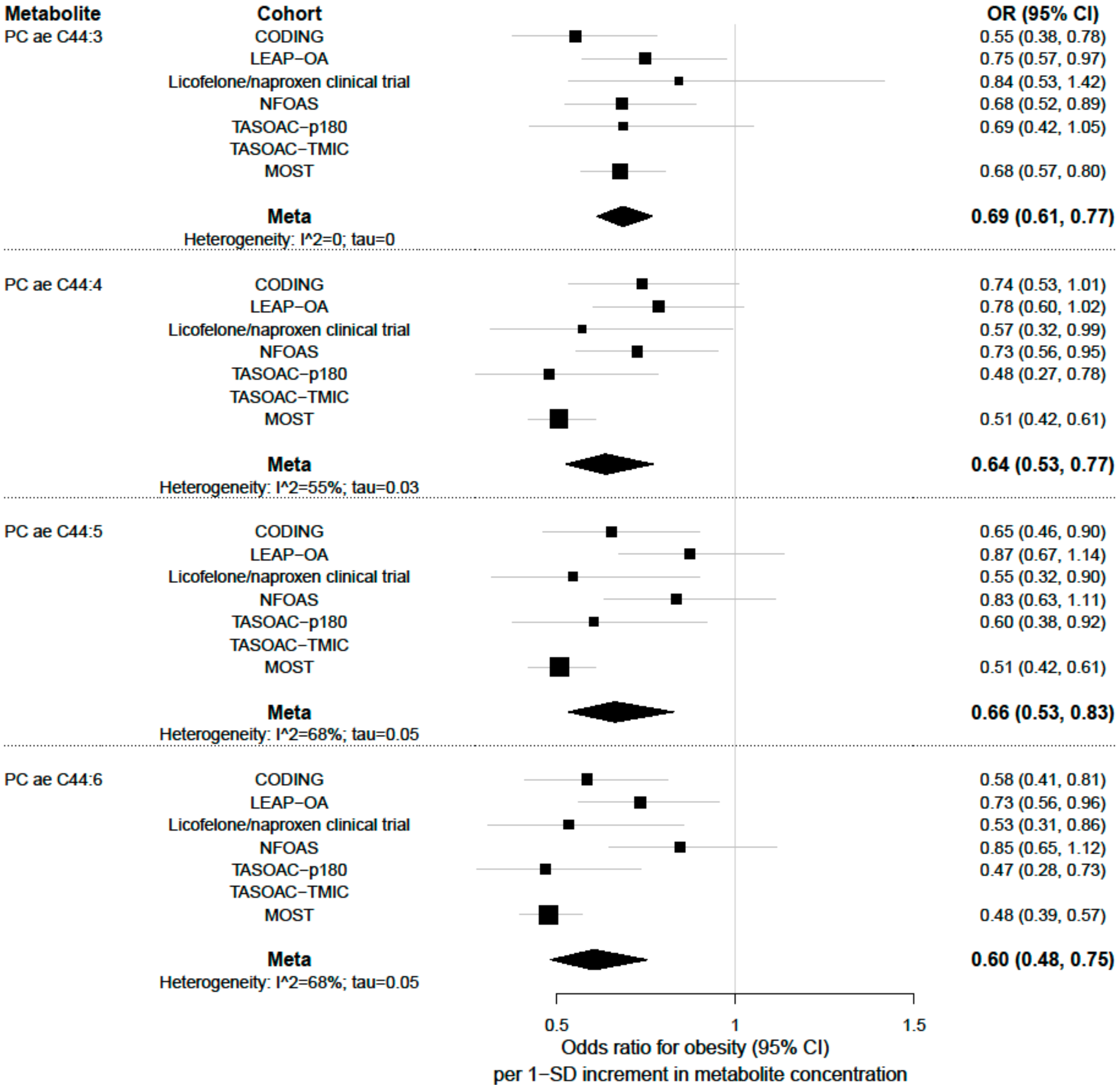

CODING: Complex Diseases in the Newfoundland population: Environment and Genetics; LEAP-OA: Longitudinal Evaluation in the Arthritis Program, Osteoarthritis Study; MOST: Multicenter Osteoarthritis Study; NFOAS: Newfoundland Osteoarthritis Study; TASOAC-p180: Tasmanian Older Adult Cohort profiled with Biocrates AbsoluteIDQ® p180 kit; TASOAC-TMIC: Tasmanian Older Adult Cohort profiled with the TMIC Prime Metabolomics Profiling Assay; a: acyl; aa: acyl-acyl; ae: acyl-alkyl; C0: carnitine; C3: propionylcarnitine; Cx:y: where x is the number of carbons in the fatty acid side chain; y is the number of double bonds in the fatty acid side chain; PC: phophatidylcholine; OR: odds ratio; CI: confidence interval; SD: standard error.

OR and CI for individual cohorts were obtained by multivariable logistic regression adjusting for age, sex, and osteoarthritis status. Those for meta-analysis were obtained by random effects meta-analysis with inverse variance as weights on the summary statistics from each cohort.

Figure S3. Phospholipid metabolic pathways associated with altered blood lipid profiles in obesity

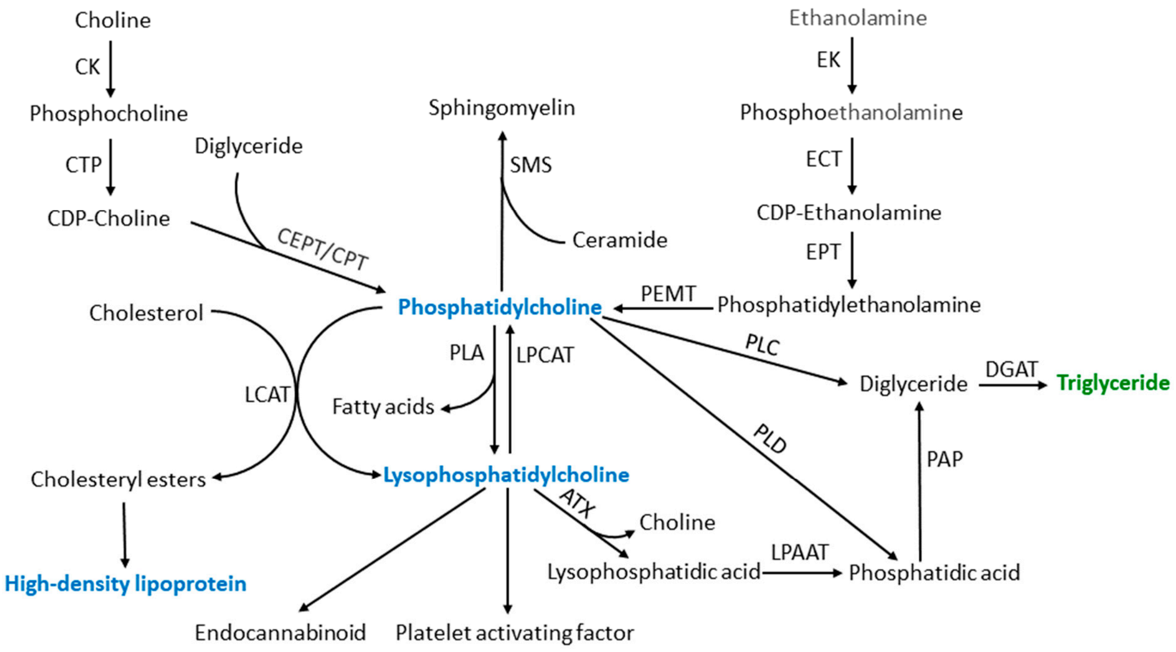

ATX: autotaxin; CDP-Choline: cytidine diphosphate-choline; CDP-Ethanolamine: cytidine diphosphate ethanolamine; CEPT: choline/ethanolamine phosphotransferase; CK: choline kinase; CTP: phosphocholine cytidylyltransferase; CPT: choline Phosphotransferase; DGAT: diacylglycerol-O-acyltransferase; ECT: phosphoethanolamine cytidylyltransferase; EK: ethanolamine kinase; EPT: ethanolamine phosphotransferase; LCAT: lecithin:cholesterol acyltransferase; LPAAT: lysophosphatidic acid acyltransferase; LPCAT: lysophosphatidylcholine acyltransferase; PAP: phosphatidic acid phosphatase; PEMT: phosphatidylethanolamine N-methyltransferase; PLA: phospholipase A; PLC: phospholipase C; PLD: phospholipase D; SMS: sphingomyelin synthase.

Bold font indicates compounds significantly associated with obesity. Blue indicates compounds negatively associated with obesity; green indicates compounds positively associated with obesity.

Figure S4. Single whole body projection scan and lean volume of mice

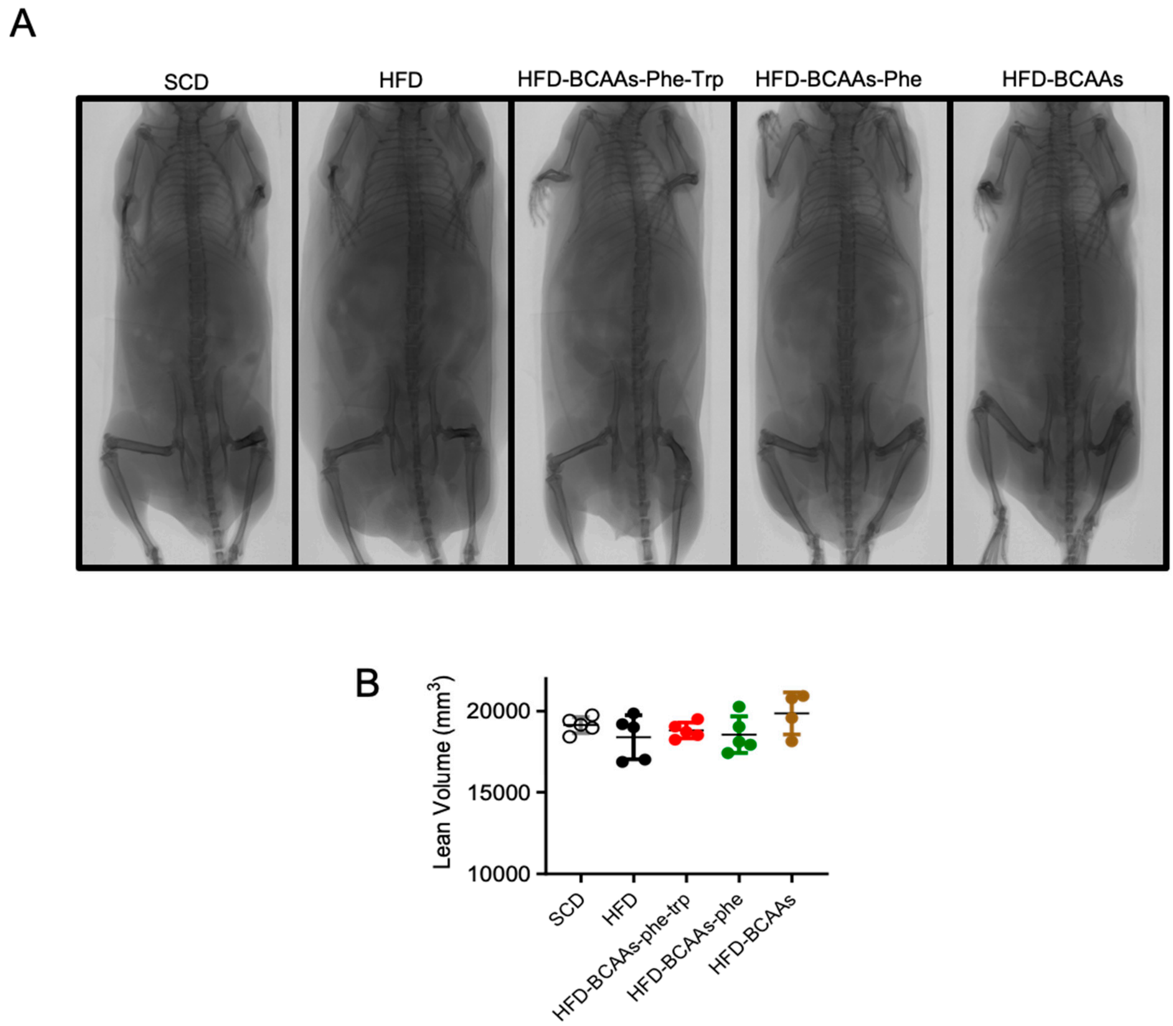

The single whole body projection scan (A) and lean volume (B) were collected and quantified in mice fed with HFD with or without restrictions of BCAAs, phenylalanine and tryptophan for 16 weeks.

BCAA: branched-chain amino acids; SCD: standard chow diet; HFD: high fat diet; HFD-BCAAs: high fat diet with 2/3 reduction of branched-chain amino acids; HFD-BCAAs-Phe: high fat diet with 2/3 reduction of branched-chain amino acids and phenylalanine; HFD-BCAAs-Phe-Trp: high fat diet with 2/3 reduction of branched-chain amino acids, phenylalanine, and tryptophan.

Figure S5. Serum levels of free fatty acid and triglycerides and lipids in liver in mice

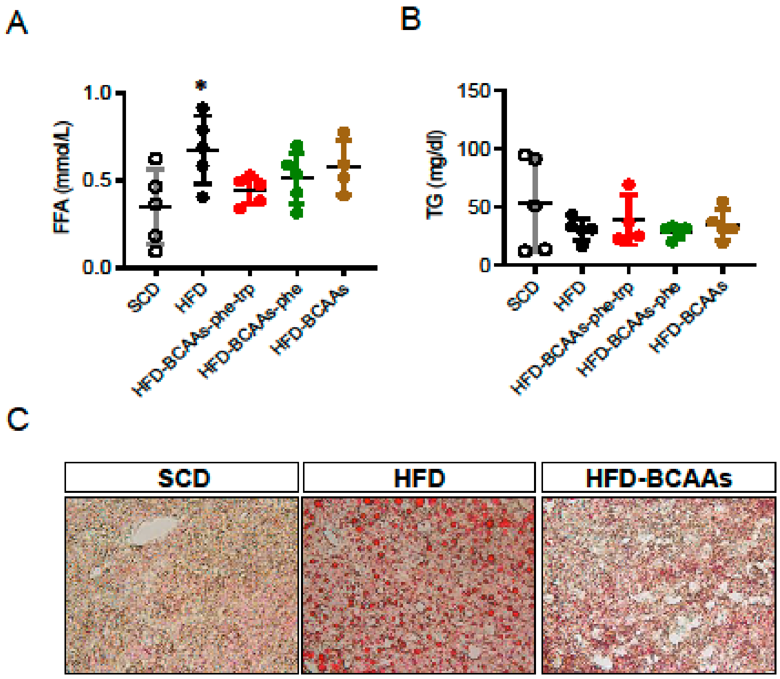

The serum levels of free fatty acid (A) and triglycerides (B) were quantified in mice fed high fat diet combining with or without restrictions in BCAAs, phenylalanine and tryptophan for 16 weeks. Lipids content in liver (C) of mice in SCD, HFD and HFD-BCAA groups was detected with Oil red O staining.

BCAA: branched-chain amino acids; SCD: standard chow diet; HFD: high fat diet; HFD-BCAAs: high fat diet with 2/3 reduction of branched-chain amino acids; HFD-BCAAs-Phe: high fat diet with 2/3 reduction of branched-chain amino acids and phenylalanine; HFD-BCAAs-Phe-Trp: high fat diet with 2/3 reduction of branched-chain amino acids, phenylalanine, and tryptophan. FFA: free fatty acids; TG: triglycerides.

Figure S6. FACS gating strategy for M1 and M2 macrophages in mice adipose tissue

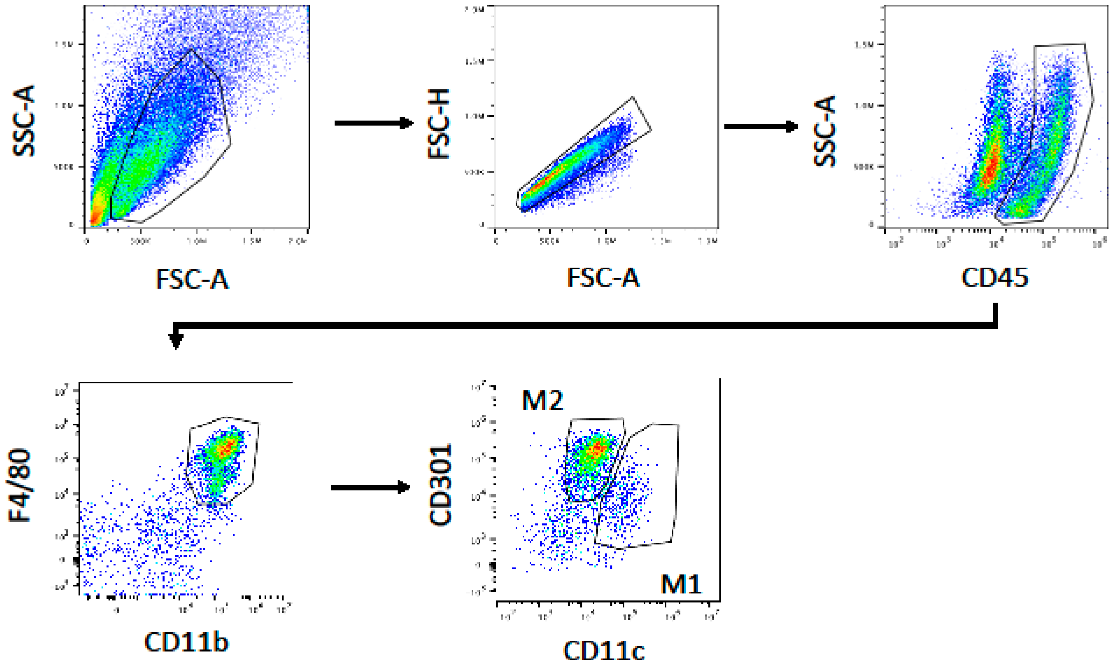

FACS: Fluorescence-activated cell sorting.

Figure S7. Traffic light plot for risk of bias assessment for studies included in the meta-analysis

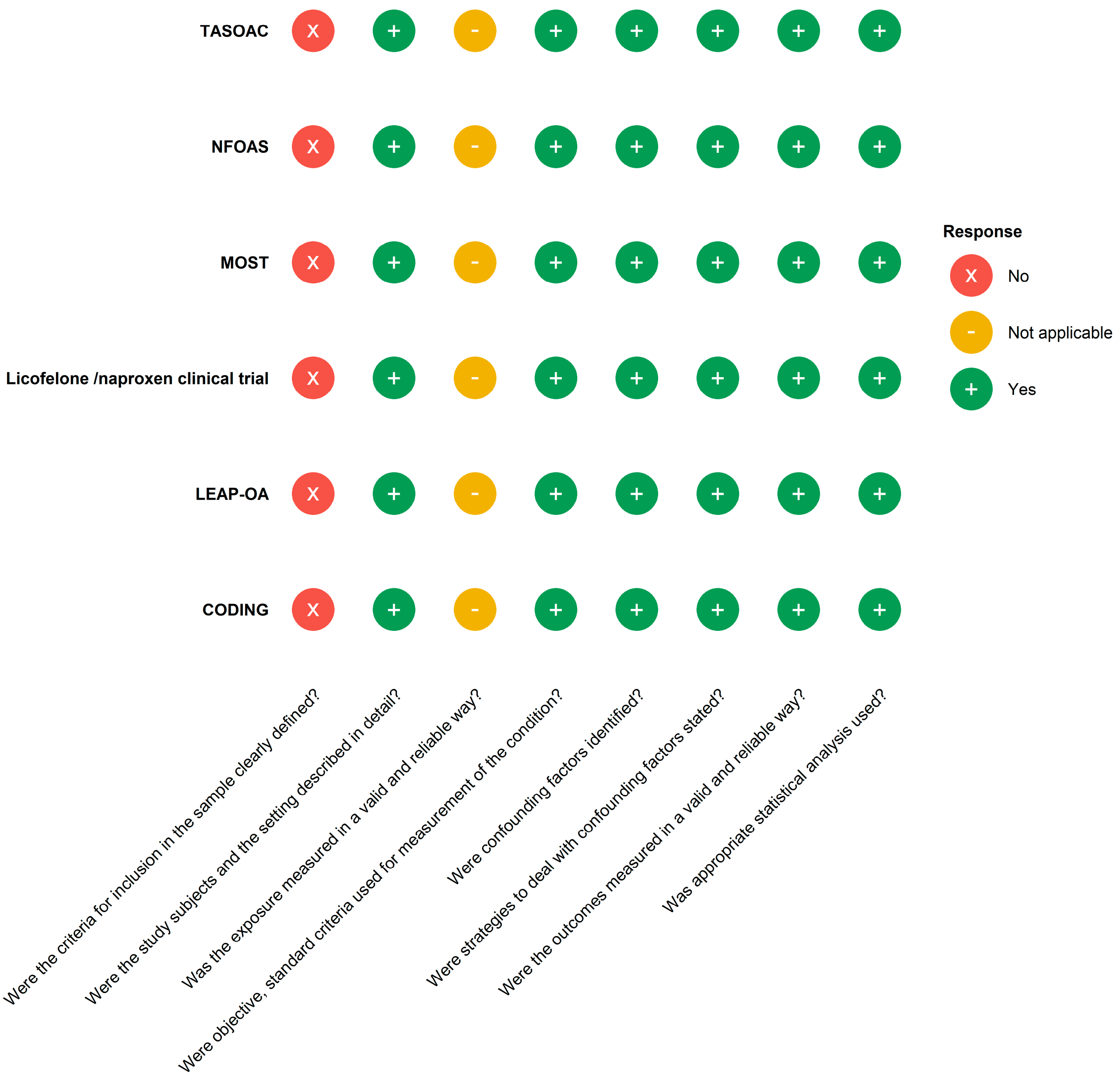

Figure S8. Body weights and biochemical parameters in mice in SCD groups

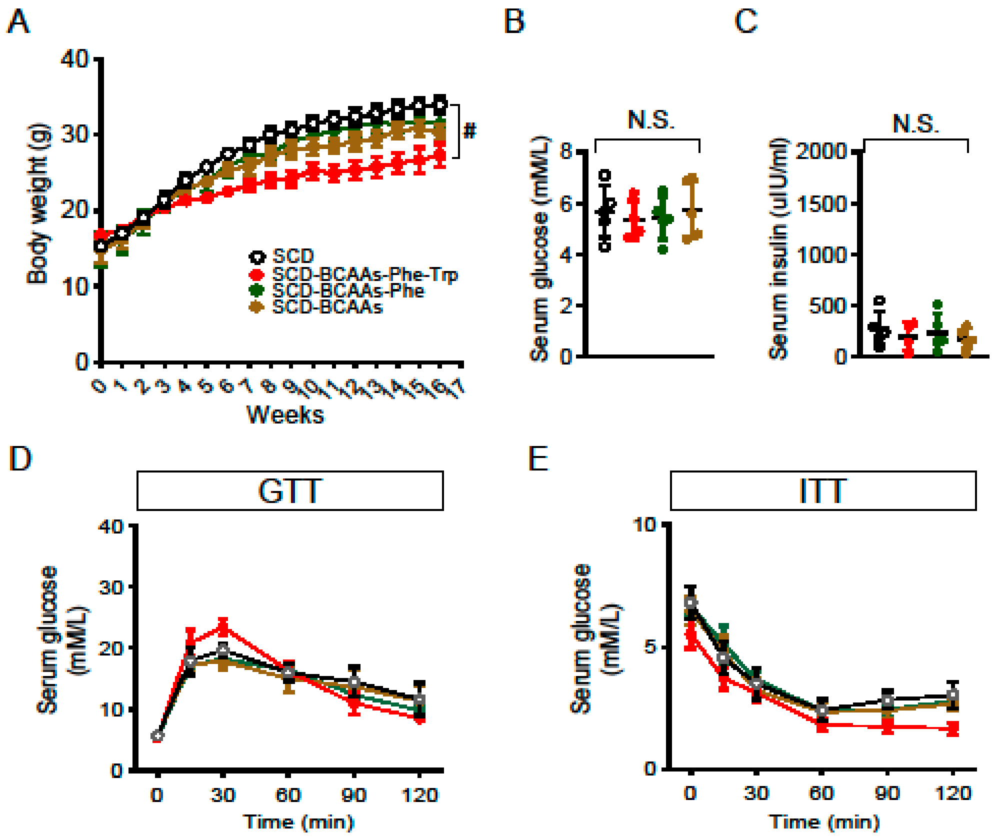

Body weights (A) were monitored in C57BL/6J mice fed with normal chow in the presence or absence of restrictions in BCAAs, phenylalanine and tryptophan for 16 weeks. Following feeding, serum glucose (B) and insulin (C), and GTT (D) and ITT (E) were quantified subsequently.

BCAA: branched-chain amino acids; SCD: standard chow diet; SCD-BCAAs: standard chow diet with 2/3 reduction of branched-chain amino acids; SCD-BCAAs-Phe: standard chow diet with 2/3 reduction of branched-chain amino acids and phenylalanine; SCD-BCAAs-Phe-Trp: standard chow diet with 2/3 reduction of branched-chain amino acids, phenylalanine, and tryptophan; GTT: glucose tolerance test; ITT: insulin tolerance test; N.S.: not significant.

References

[1] Gao X, Zhang W, Wang Y, Pedram P, Cahill F, Zhai G, et al. Serum metabolic biomarkers distinguish metabolically healthy peripherally obese from unhealthy centrally obese individuals. Nutr Metab (Lond). 2016;13:33.10.1186/s12986-016-0095-9

[2] Zhang W, Sun G, Aitken D, Likhodii S, Liu M, Martin G, et al. Lysophosphatidylcholines to phosphatidylcholines ratio predicts advanced knee osteoarthritis. Rheumatology (Oxford). 2016;55:1566-74.10.1093/rheumatology/kew207

[3] Power JD, Perruccio AV, Gandhi R, Veillette C, Davey JR, Syed K, et al. Neuropathic pain in end-stage hip and knee osteoarthritis: differential associations with patient-reported pain at rest and pain on activity. Osteoarthritis Cartilage. 2018;26:363-9.10.1016/j.joca.2018.01.002

[4] Raynauld JP, Martel-Pelletier J, Bias P, Laufer S, Haraoui B, Choquette D, et al. Protective effects of licofelone, a 5-lipoxygenase and cyclo-oxygenase inhibitor, versus naproxen on cartilage loss in knee osteoarthritis: a first multicentre clinical trial using quantitative MRI. Ann Rheum Dis. 2009;68:938-47.10.1136/ard.2008.088732

[5] Zhai G, Pelletier JP, Liu M, Aitken D, Randell E, Rahman P, et al. Activation of The Phosphatidylcholine to Lysophosphatidylcholine Pathway Is Associated with Osteoarthritis Knee Cartilage Volume Loss Over Time. Sci Rep. 2019;9:9648.10.1038/s41598-019-46185-w

[6] Zhai G, Pelletier JP, Liu M, Randell EW, Rahman P, Martel-Pelletier J. Serum lysophosphatidylcholines to phosphatidylcholines ratio is associated with symptomatic responders to symptomatic drugs in knee osteoarthritis patients. Arthritis Res Ther. 2019;21:224.10.1186/s13075-019-2006-8

[7] Segal NA, Nevitt MC, Gross KD, Hietpas J, Glass NA, Lewis CE, et al. The Multicenter Osteoarthritis Study: opportunities for rehabilitation research. PM R. 2013;5:647-54.10.1016/j.pmrj.2013.04.014

[8] Zhai G, Sun X, Randell EW, Liu M, Wang N, Tolstykh I, et al. Phenylalanine Is a Novel Marker for Radiographic Knee Osteoarthritis Progression: The MOST Study. J Rheumatol. 2021;48:123-8.10.3899/jrheum.200054

[9] Costello CA, Hu T, Liu M, Zhang W, Furey A, Fan Z, et al. Metabolomics Signature for Non-Responders to Total Joint Replacement Surgery in Primary Osteoarthritis Patients: The Newfoundland Osteoarthritis Study. J Orthop Res. 2020;38:793-802.10.1002/jor.24529

[10] Liu M, Xie Z, Costello CA, Zhang W, Chen L, Qi D, et al. Metabolomic analysis coupled with extreme phenotype sampling identified that lysophosphatidylcholines are associated with multisite musculoskeletal pain. Pain. 2021;162:600-8.10.1097/j.pain.0000000000002052

[11] Pan F, Liu M, Randell EW, Rahman P, Jones G, Zhai G. Sphingomyelin is involved in multisite musculoskeletal pain: evidence from metabolomic analysis in two independent cohorts. Pain. 2020.10.1097/j.pain.0000000000002163

[12] Gao X, Randell E, Tian Y, Zhou H, Sun G. Low serum choline and high serum betaine levels are associated with favorable components of metabolic syndrome in Newfoundland population. J Diabetes Complications. 2019;33:107398.10.1016/j.jdiacomp.2019.06.003

[13] Gao X, Tian Y, Randell E, Zhou H, Sun G. Unfavorable Associations Between Serum Trimethylamine N-Oxide and L-Carnitine Levels With Components of Metabolic Syndrome in the Newfoundland Population. Front Endocrinol (Lausanne). 2019;10:168.10.3389/fendo.2019.00168

[14] Zhai G, Wang-Sattler R, Hart DJ, Arden NK, Hakim AJ, Illig T, et al. Serum branched-chain amino acid to histidine ratio: a novel metabolomic biomarker of knee osteoarthritis. Ann Rheum Dis. 2010;69:1227-31.ard.2009.120857 [pii]10.1136/ard.2009.120857

[15] Foroutan A, Fitzsimmons C, Mandal R, Piri-Moghadam H, Zheng J, Guo A, et al. The Bovine Metabolome. Metabolites. 2020;10.10.3390/metabo10060233

[16] Yu D, Richardson NE, Green CL, Spicer AB, Murphy ME, Flores V, et al. The adverse metabolic effects of branched-chain amino acids are mediated by isoleucine and valine. Cell Metab. 2021;33:905-22 e6.10.1016/j.cmet.2021.03.025

[17] Fontana L, Cummings NE, Arriola Apelo SI, Neuman JC, Kasza I, Schmidt BA, et al. Decreased Consumption of Branched-Chain Amino Acids Improves Metabolic Health. Cell Rep. 2016;16:520-30.10.1016/j.celrep.2016.05.092
[18] Ma L-L, Wang Y-Y, Yang Z-H, Huang D, Weng H, Zeng X-T. Methodological quality (risk of bias) assessment tools for primary and secondary medical studies: what are they and which is better? Military Medical Research. 2020;7:7.10.1186/s40779-020-00238-8
[19] Liu T, Nie X, Wu Z, Zhang Y, Feng G, Cai S, et al. Can statistic adjustment of OR minimize the potential confounding bias for meta-analysis of case-control study? A secondary data analysis. BMC Medical Research Methodology. 2017;17:179.10.1186/s12874-017-0454-x
